# Coded Taxonomy Applied to Old and New Descriptions of *Mucrosomia* (Collembola: Isotomidae): A Bibliographic Revision and New Species of the Genus

**DOI:** 10.1007/s13744-026-01397-4

**Published:** 2026-05-28

**Authors:** Estevam Cipriano Araujo de Lima, Aila Soares Ferreira, Misael Augusto de Oliveira-Neto, Bruna Carolline Honório Lopes, Nathan Paiva Brito, Roniere Andrade de Brito, Douglas Zeppelini

**Affiliations:** 1 Lab de Sistemática de Collembola E Conservação, Instituto de Biologia Do Solo, Univ Estadual da Paraíba, João Pessoa, PB Brazil; 2 Programa de Pós-Graduação em Ciências Biológicas – Zoologia, Univ Federal da Paraíba, João Pessoa, PB Brazil; 3 Lab de Estudos Subterrâneos, Depto de Ecologia E Biologia Evolutiva, Univ Federal de São Carlos, São Carlos, SP Brazil; 4 Programa de Pós-Graduação Em Ecologia E Recursos Naturais, Univ Federal de São Carlos, São Carlos, SP Brazil

**Keywords:** Arthropoda, Hexapoda, Morphological coding, Taxonomic standardization

## Abstract

Collembola are minute, wingless hexapods that occur in nearly all terrestrial ecosystems and playing a fundamental role in soil functioning and global carbon cycling. The taxonomy within the family Isotomidae is unclear due to the strong morphological similarity among species, low interspecific variability, and overlapping chaetotaxic patterns that often obscure species boundaries. These challenges highlight the need for standardized approaches that minimize subjectivity in descriptions and enable comparability across taxa. The genus *Mucrosomia* is a small but significant lineage within Isotomidae, comprising four recognized species distributed across distinct habitats worldwide. In this study, we provide the first comprehensive revision of *Mucrosomia* using Coded Taxonomy, a framework that translates morphological characters into coded matrices, enabling reproducibility and integration with digital platforms. We describe two new species, *Mucrosomia janssensi* sp. nov. and *Mucrosomia potapovi* sp. nov., and attempt to code the previously described species under the new standardized system. Our results establish a robust comparative basis for the genus and demonstrate the potential of coded approaches to advance Collembola taxonomy.

**Supplementary Information:**

The online version contains supplementary material available at 10.1007/s13744-026-01397-4.

## Introduction

The Collembola class Lubbock 1870 (Lubbock 1870) comprises minute, wingless, basal hexapods with entognathous mouthparts, occurring in virtually all terrestrial habitats, including soil, leaf litter, tree canopies, and caves (Bellinger et al. 1996; Hopkin 1997). Their global soil biomass has been estimated at 27.5 megatons of carbon, three times greater than that of wild terrestrial vertebrates, with peak densities reaching 2 million individuals per square meter in tundra ecosystems (Potapov et al. 2023), reflecting their ecological relevance in soil food webs and global carbon cycling (Rusek 1998; Bardgett and Van Der Putten 2014; Filser et al. 2016; Potapov et al. 2023). Approximately 9000 species have been described, although true diversity remains largely underestimated (Bellinger et al 1996; Hopkin 1997).

The family Isotomidae (Schäffer 1896), within the order Entomobryomorpha, represents a diverse and widely distributed lineage of Collembola (Sánchez-García and Engel 2016). Although relatively well studied, its taxonomy remains challenging due to the small body size and strong morphological similarities among species, which require detailed microscopic examination for accurate identification (Christiansen et al. 2009).

The identification of Isotomidae species is made difficult by extensive morphological similarity and the conservative nature of many diagnostic traits. Interspecific morphological uniformity and long-term stasis, as observed in fossil lineages such as *Proisotoma communis* (Sánchez-García and Engel 2016), highlight the persistence of conserved features across evolutionary time (Sánchez-García & Engel 2016). Among the most problematic traits is chaetotaxy, which, although widely used, shows significant overlap between species. Within *Isotomurus*, for example, reliance on chaetal patterns often results in ambiguous diagnoses (Carapelli et al. 2001), while in *Cryptopygus* and related genera, overlapping chaetotaxic arrangements demand highly detailed analysis for accurate classification (Potapov et al. 2020).

To address limitations in traditional morphological descriptions, the newly proposed taxonomic notation method, Coded Taxonomy (CT) (Zeppelini et al. 2024), attempts to replace subjective and often redundant texts with coded character matrices, enhancing comparability and accelerating species descriptions while enabling broader data integration, from interactive keys to machine learning applications.

In this study, we provide a comprehensive revision of the genus *Mucrosomia* (Bagnall 1949), based on published descriptions, currently comprising five recognized species: *M. alticola* (Mendonça and Queiroz 2013), *M. bipartita* (Rusek 1946), *M. caeca* (Wahlgren 1906), *M. garretti* (Bagnall 1939), and *M. novaezealandiae* (Salmon 1943). Using the framework of CT (Zeppelini et al. 2024), we describe two new species, *M. janssensi* sp. nov. and *M. potapovi* sp. nov., and reanalyze all previously known taxa through standardized coded characters, establishing a consistent and comparable basis for the genus.

## Material and methods

### Coded description

For the revisions and descriptions, we used the method of CT (Zeppelini et al. 2024); the method consists of presenting the general morphology in a coded matrix, based on a list of characters with all observed condition for each character in the list. The morphological features are presented in a pictorial matrix, side by side at the same scale. The images in the matrix are obtained mainly by SEM, or the best available resolution. The description is presented as a matrix column, containing codes of 0,1,2, to each and every character described, sided by the corresponding morphologic illustration. The illustration can be either photographic images or schematic line drawings, depending on the feature. The presentation results in a descriptive template.

As an example of the readability of the character description in the character list (Table 1), we can take one character in the list below:

- (Structure) Labral chaetotaxy
- (Character) Labral formula (a+m+p+pl):
- (Observed condition) 1–4+5+5+4 9(1)9(4)
- (Observed condition) 2 – different combination

**Table 1:** Character list for the genus *Mucrosomia*, including structure, character, and observed condition

Pigments				**1** – absent. **2** – present
Body color				**1** – white. **2** – pale yellow
Head	Segment I	Eye	Eye number (EN)	**1** – 0 + 0. **2** – different combination
		PAO	Post-antennal organ form (PAO)	**1** – absent. **2** – present entire. **3** – present with constriction in the middle
		Clipeal	Clypeal area I (CI)	**1** – 1 + 1 2(1). **2** – different combination
			Clypeal area II (CII)	**1** – 1 + 1 2(1). **2** – different combination
			Clypeal area III (CIII)	**1** – 1 1(1). **2** – different combination
			Clypeal area IV (CIV)	**1** – 1 + 1 + 1 3(1). **2** – different combination
		Labral	Labral formula (a + m + p + pl)	**1** – 4 + 5 + 5 + 3 8(1)9(2). **2** – 4 + 5 + 5 + 4 7(1)11(2)
	Segment II	Antenal area	Antennal area chaetotaxy (ANTC)	**1** – 17 + 2 + 17 36(1). **2** – 24 + 2 + 24 50(1)
	Segmento IV	Mandibular	Mandibular area chaetotaxy (MD)	**1** – 15 + 3 + 15 33(1). **2** – 19 + 2 + 19 40(1)
	Segment V	Maxillary area	Maxillary area chaetotaxy dorsal (MX D)	**1** – 29 + 2 + 29 60(1). **2** – 39 + 2 + 39 80(1)
			Maxillary area chaetotaxy dorsal lateral and ventral (MX L + V)	**1** – 52 + 52 104(1). **2** – 68 + 68 136(1)
		Maxillary lobe	Lobe Maxillary distal area (LMX D)	**1** – 6 + 6 2(1)8(6)2(7). **2** – 7 + 7 2(1)8(6)2(7)2(11). **3** – 7 + 7 2(1)10(6)2(7)
			Lobe Maxillary basal area (LMX B)	**1** – 1 + 1 1(1). **2** – different combination
	Segment VI	Labial	Labial area chaetotaxy dorsal and ventral (LB D + V)	**1** – 20 + 20 40(1). **2** – 17 + 17 34(1)
			Postlabial (PostL)	**1** – 5 + 5 10(1). **2** – 4 + 4 8(1)
			Labial triangle total chaetae (LBT)	**1** – 9 + 9 18(1). **2** – 10 + 10 20(1)
			Labial proximal chaetae and papillae chaetotaxy count (LBP)	**1** – 26 + 26 8(1)8(3)2(4)2(5)32(6). **2** – different combination
Body	Body	Thorax I	Thorax I (ThI)	**1** – 0 + 0. **2** – different combination
		Thorax II	Thorax II (ThII)	**1** – 6 + 6 2(8)8(9)2(10). **2** – different combination
		Thorax III	Thorax III (ThIII)	**1** – 4 + 4 2(8)6(9). **2** – different combination
			Thorax III area ventralmedial (VM)	**1** – 3 + 3 4(1)2(8). **2** – 0 + 0
	Abdome	Abdomen I	Abdomen I (AbdI)	**1** – 6 + 6 6(8)4(9)2(10). **2** – 4 + 4 2(8)4(9)2(10)
		Abdomen II	Abdomen II (AbdII)	**1** – 5 + 5 6(8)4(9). **2** – different combination
		Abdomen III	Abdomen III (AbdIII)	**1** – 5 + 5 6(8)4(9). **2** – different combination
		Abdomen IV	Abdomen IV (AbdIV)	**1** – 7 + 7 8(8)6(9). **2** – different combination
			Abdomen Parafurcal region (AbdPF)	**1** – 19 + 19 36(1)2(8). **2** – 18 + 18 34(1)2(8). **3** – 17 + 17 32(1)2(8)
		Abdomen V + VI	Abdomen V and VI sensillar count (AbdV + VI)	**1** – 4 + 4 8(9). **2** – 5 + 5 10(9)
Head apendagges	Antenna	Antenna I	Antenna I whorl A total chaetae (ANTI A)	**1** – 15 12(1)2(12). **2** – 12 10(1)2(12)**3** – 13 10(1)3(12)
			Antenna I whorl B total chaetae (ANTI B)	**1** – 2 2(11). **2** – different combination
		Antenna II	Antenna II whorl A total chaetae (ANTII A)	**1** – 8 7(1)1(12). **2** – different combination
			Antenna II whorls −I 0 and +I total chaetae (ANTII −0+)	**1** – 12 12(1). **2** – 15 15(1). **3** – 10 10(1)
			Antenna II whorl B total chaetae (ANTII B)	**1** – 5 3(1)2(11). **2** – 5 4(1)1(11)
		Antenna III	Antenna III whorl A total chaetae (ANTIII A)	**1** – 9 4(1)2(12)2(13)1(14). **2** – different combination
			Antenna III whorls −I 0 and +I total chaetae (ANTIII −0+)	**1** – 12 12(1). **2** – 18 18(1). **3** – 16 16(1)
			Antenna III whorl B total chaetae (ANTIII B)	**1** – 2 1(1)1(11). **2** – 1 1(11)
		Antenna IV	Antenna IV A total chaetae (ANTIV A)	**1** – 65 44(1)5(11)10(9)5(12)1(15). **2** – 82 52(1)14(11)8(9)7(12)1(15)
			Antenna IV B total chaetae (ANTIV B)	**1** – 8 8(1). **2** – different combination
Body apendagges	Legs	Subcoxa	Subcoxa I area I total chaetae (SCXI I)	**1** – 0. **2** – different combination
			Subcoxa I area II total chaetae (SCXI II)	**1** – 1 1(1). **2** – different combination
			Subcoxa II area I total chaetae (SCXII I)	**1** – 1 1(1). **2** – different combination
			Subcoxa II area II total chaetae (SCXII II)	**1** – 6 6(1). **2** – different combination
			Subcoxa III area I total chaetae (SCXIII I)	**1** – 5 5(1). **2** – different combination
			Subcoxa III area II total chaetae (SCXIII II)	**1** – 8 8(1). **2** – 7 7(1)
		Coxa I	Coxa I total chaetae (CXI)	**1** – 3 3(1). **2** – 4 4(1)
			Coxa II total chaetae (CXII)	**1** – 12 12(1). **2** – different combination
			Coxa III total chaetae (CXIII)	**1** – 11 11(1). **2** – different combination
		Trochanter	Trochanter I total chaetae (TRI)	**1** – 9 9(1). **2** – different combination
			Trochanter II total chaetae (TRII)	**1** – 9 9(1). **2** – different combination
			Trochanter III total chaetae (TRIII)	**1** – 10 10(1). **2** – 8 8(1)
		Femur	Femur I total chaetae (FEI)	**1** – 17 17(1). **2** – different combination
			Femur II total chaetae (FEII)	**1** – 18 18(1). **2** – different combination
			Femur III total chaetae (FEIII)	**1** – 21 21(1). **2** – different combination
		Tibiotarsi	Tibiotarsus I whorl I total chaetae (TibI I)	**1** – 7 7(1). **2** – different combination
			Tibiotarsus I whorl II total chaetae (TibI II)	**1** – 7 7(1). **2** – different combination
			Tibiotarsus I whorl III total chaetae (TibI III)	**1** – 7 7(1). **2** – different combination
			Tibiotarsus I whorl IV (TibI IV)	**1** – absent. **2** – present
			Tibiotarsus I whorl V (TibI V)	**1** – absent. **2** – present
			Tibiotarsus II whorl I total chaetae (TibII I)	**1** – 7 7(1). **2** – different combination
			Tibiotarsus II whorl II total chaetae (TibII II)	**1** – 7 7(1). **2** – different combination
			Tibiotarsus II whorl III total chaetae (TibII III)	**1** – 7 7(1). **2** – different combination
			Tibiotarsus II whorl IV (TibII IV)	**1** – absent. **2** – present
			Tibiotarsus II whorl V (TibII V)	**1** – absent. **2** – present
			Tibiotarsus III whorl I total chaetae (TibIII I)	**1** – 12 12(1). **2** – different combination
			Tibiotarsus III whorl II total chaetae (TibIII II)	**1** – 7 7(1). **2** – different combination
			Tibiotarsus III whorl III total chaetae (TibIII III)	**1** – 7 7(1). **2** – different combination
			Tibiotarsus III whorl IV (TibIII IV)	**1** – absent. **2** – present
			Tibiotarsus III whorl V (TibIII V)	**1** – absent. **2** – present
		Pretarsusi	Pretarsus I pretarsal chaetae (PTI)	**1** – 2 2(11). **2** – different combination
			Pretarsus II pretarsal chaetae (PTII)	**1** – 2 2(11). **2** – different combination
			Pretarsus III pretarsal chaetae (PTIII)	**1** – 2 2(11). **2** – different combination
		Unguis	Unguis I inner tooth (UIit)	**1** – present. **2** – absent
			Unguis I tunica (UIt)	**1** – absent. **2** – present
			Unguis II inner tooth (UIIit)	**1** – present. **2** – absent
			Unguis II tunica (UIIt)	**1** – absent. **2** – present
			Unguis III inner tooth (UIIIit)	**1** – present. **2** – absent
			Unguis III tunica (UIIIt)	**1** – absent. **2** – present
		Unguiculus	Unguiculus I inner tooth (UNIit)	**1** – absent. **2** – present
			Unguiculus I apical filament (UNIapf)	**1** – absent. **2** – present
			Unguiculus II inner tooth (UNIIit)	**1** – absent. **2** – present
			Unguiculus II apical filament (UNIIapf)	**1** – absent. **2** – present
			Unguiculus III inner tooth (UNIIIit)	**1** – absent. **2** – present
			Unguiculus III apical filament (UNIIIapf)	**1** – absent. **2** – present
	Collophore	Anterior side	Anterior side total chaetae (AS)	**1** – 0. **2** – different combination
		Lateral flap	Lateral flap total chaetae (LF)	**1** – 6 + 6 12(1). **2** – 5 + 5 10(1). **3** – 4–7 + 4–7 8–14(1). **4** – 7 + 7 14(1). **5** – 5–6 + 5–6 10–12(1)
		Posterior side	Posterior side total chaetae (PS)	**1** – 5 + 5 10(1). **2** – 4 + 4 8(1). **3** – 3 + 3 6(1)
	Tenaculum	Copus tenacular	Tenaculum rami (TR)	**1** – 4 + 4 teeth. **2** – different combination
			Tenaculum corpus (a + p) (TC a + p)	**1** – 1 1(1). **2** – 2 2(1)
	Furcula	Manubrium	Anterior manubrium total chaetae (MNA)	**1** – 1 + 1 2(16). **2** – different combination
			Posterior manubrium total chaetae (NMP)	**1** – 9 + 9 18(1). **2** – 10 + 10 20(1)
		Dens	Dental whorl I total chaetae (DNI)	**1** – 3 3(17). **2** – different combination
			Dental whorl II total chaetae (DNII)	**1** – 3 3(17). **2** – different combination
			Dental whorl III total chaetae (DNIII)	**1** – 4 2(1)2(17). **2** – different combination
			Dental whorl IV total chaetae (DNIV)	**1** – 4 2(1)2(17). **2** – different combination
			Dental whorl V total chaetae (DNV)	**1** – 1 1(17). **2** – different combination
			Dental anterior total chaetae (DenA)	**1** – 10 + 10 20(17). **2** – different combination
			Dental posterior total chaetae (DenP)	**1** – 5 + 5 8(1)2(17). **2** – different combination
		Mucro	Mucro lamellae total teeth (MU)	**1** – 5. **2** – different combination

In the description template, there will be a code (1 or 2). The meaning of the description 4+5+5+4 9(1)9(4) is that there is a total of 18 chaetae, distributed in the rows a (4),m (5),p (5),pl (4), where nine chaetae are the type (1) in the pictorial matrix, and nine chaetae are type (4) in the matrix. This avoids the necessity to use adjectives to describe some morphologic features (as different types of chaetae), reducing the ambiguity.

The codification of the head was modified from Zeppelini et al. (2024) and De Oliveira and Zeppelini (2024), incorporating additional morphological features based on the studies of Altner and Thies (1976) and Tomizuka and Machida (2015). In this approach, the head is divided into the following segments:

- Segment I: Eye, post-antennal, and clypeus/labrum areas
- Segment II: Antennal area
- Segment III: Intercalary (absent)
- Segment IV: Mandibular area
- Segment V: Maxillary area, including the maxillary lobe
- Segment VI: Labial area, including labial chaetae and the anterior third of the ventral groove

All descriptive diagnoses were automatically generated using the FreeDELTA Editor through Descriptions → Natural Language (CONFOR directives). The set of characters retained corresponds exclusively to those traditionally employed in species-level diagnoses within the genus and follows the most recent generic framework established in the description of *Mucrosomia aticola* by Mendonça and Queiroz (2013). Characters (2, 5–8, 10–13, 16, 40–54, 70–72, 74, 76, 78–84, and 92–96) were excluded because they are not diagnostic at the species level within the genus.

Supplementary Material contains the complete FreeDELTA Editor dataset (Mucrosomia Database.dtz). From this database, we provide the full descriptions of all Mucrosomia species and a comparative table of species with their respective character states, both automatically generated by FreeDELTA. It also includes the coded descriptions of *M. alticola*, *M. bipartita*, *M. caeca*, *M. garretti*, and* M. novaezealandiae.* In addition, S1 includes an interactive map showing the global distribution of the genus, with exact coordinates for the newly described species and approximate localities for previously described species based on the available literature.

### Terminology

The homology of the characters was accessed in accordance with the ontogenetic and morphologic studies for the group, as follows: antennal sens and chaetal disposition followed Deharveng 1983; Nayrolles 1991; Potapov 2001; Deharveng et al. 2020; labial chaetotaxy followed Gisin 1967; Zhang and Pan 2020; labial papillae, maxillary palp, and basolateral and basomedial labial fields as in Fjellberg 1999; postlabial chaetotaxy followed Chen and Christiansen 1993, with modifications of Cipola et al. 2018; clypeal chaetotaxy as Yoshii and Suhardjono 1992; labral chaetotaxy followed Cipola et al. 2014; thoracic and abdominal dorsal chaetotaxy as defined in Potapov 2001; legs followed Lawrence 1977; Deharveng 1983; Nayrolles 1998; and Potapov 2001; dens chaetotaxy according to Nayrolles 1990.

### Material analyzed

The specimens were preserved in 70% ethanol and mounted on slides following Jordana et al. (1997), after clearing with Nesbitt’s solution. Line drawings and morphological studies were conducted using a phase-contrast microscope equipped with a digital camera and a camera lucida. The type material is deposited in the Reference Collection of Soil Fauna (CRFS-UEPB). The revision and coding of the description of all known *Mucrosomia* species were based on the available literature.

### Electronic microscopy and macrophotography

For the study under scanning electron microscope (SEM), the specimens were dehydrated by graded series of ethanol, dried in a critical point dryer model Autosamdri-931 by Tousimis, metallized with system for electron microscopes, HP Desk V model by Denton Vacuum, and observed in SEM by Tescan Vega III model. Previously, the samples were hydrated for cleaning and rinsing, by a graded ethanol treatment with immersion for 20 min in 90% ethanol, then transferred to 60% ethanol, to 30% ethanol, and distilled water. After this initial process, the specimens were gradually dehydrated using 30%, 60%, and 90% ethanol, with 20 min in each concentration. Finally, the samples were placed in 99.8% ethanol and transferred to the critical point dryer. Figure and illustrations were generated and assembled using CorelDRAW Graphics Suite 2020 for Windows (https://www.coreldraw.com).

## Results

### Taxonomy

Entomobryomorpha Börner 1913

Familia Isotomidae Schäffer 1896

Subfamilia Anurophorinae Börner 1901

Genus *Mucrosomia* Bagnall 1949

zoobank.org:act:32B1BA8A-A0E8-4D43-8D77-D4F93C4E7FFE

**Fig. 1:**
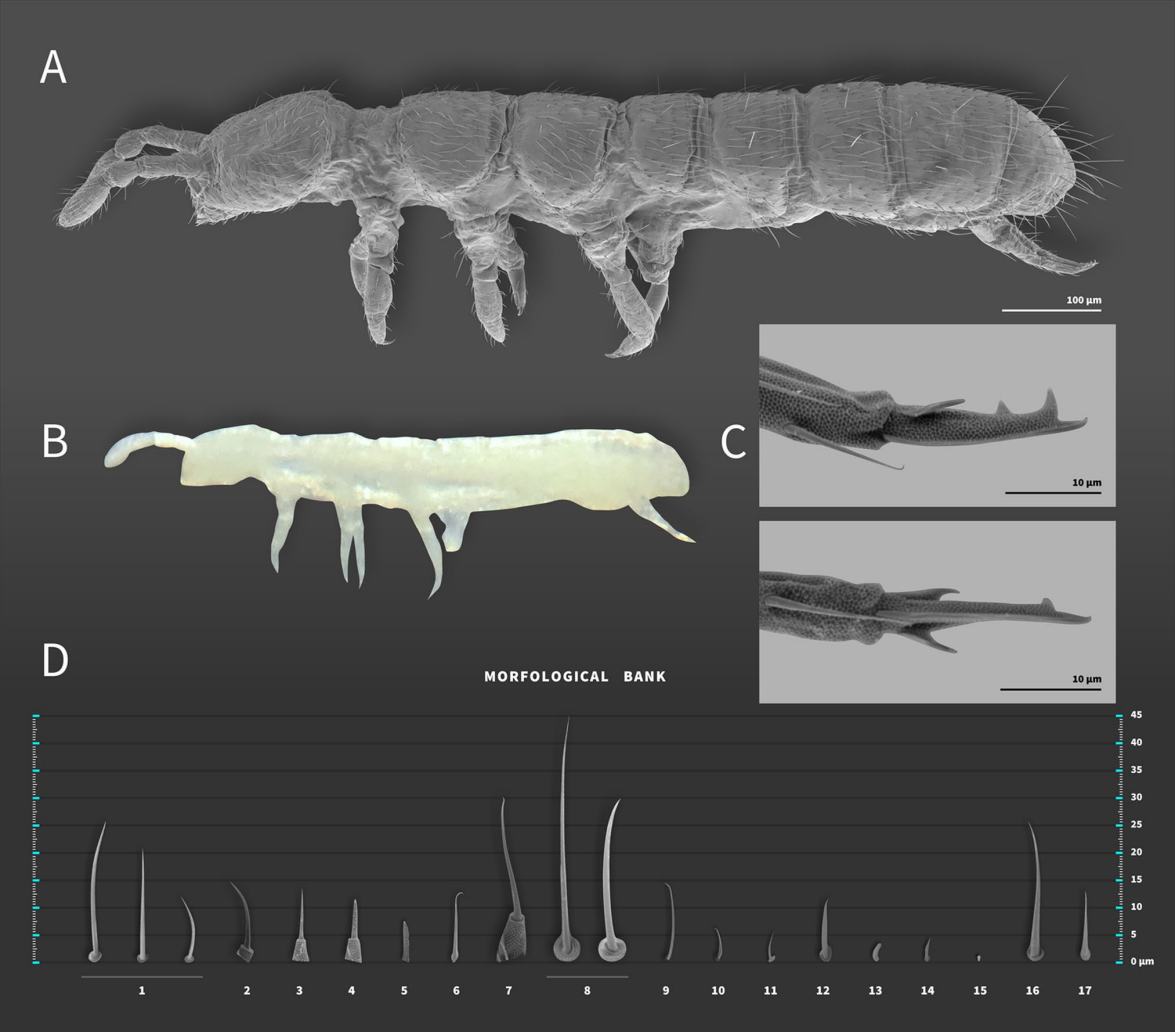
*Mucrosomia janssensi* **sp. n.** Genus diagnosis. **A** Habitus of SEM. **B** Habitus in alcohol. **C** Mucro. **D** Morphological Bank—a collection of different chaetae found along the body. The chaetae are represented in their shapes and sizes; therefore, a given shape and size represented in the bank may occur in different parts of the body: 1–Smooth common chaeta (usually all body); 2–Spiniform chaeta with papillae (e.g., labral **a** and **m**); 3–Mesosensillum with papillae (e.g., mesosensilum of the labial palp–**C**); 4–Mesosensillum with rod-shaped papillae (e.g., mesosensilum of the labial palp–**A**); 5–Rod-shaped mesosensillum (e.g., labial palp, finger shape lateral process (l.p.)); 6–curved Mesosensillum inserted in a triangular socket (e.g., mesosensilum labial palp–**ai**); 7–Macrosensillum with papilla; 8–Smooth macrochaeta/mesochaeta; 9–Smooth macrosensillum; 10–Smooth Microsensillum; 11–Microchaetae; 12–Thick macrosensillum (present on the antenna); 13–Thick and blunt microsensillum (occurs in pairs on the Ant. III apical organ); 14–Flat and acuminate microsensillum (present on Ant. III short dorso-external); 15–Subapical organ in Ant. IV; 16–Smooth and lanceolate chaeta; 17–Spine-like chaeta

**Diagnosis**. *Mucrosomia* Bagnall 1949 modified from Potapov (2001) and Fjellberg (2007) (Fig. 1). A small group of blind species close to *Cryptopygus*, which are characterized by the remarkable quinquedentate mucro. Colorless and blind species. Post-antennal organ present. Furca complete, manubrium with 1+1 anterior chaetae, long dens, and a distinctive quinquedentate mucro (5 teeth: 3 apical and 2 basal). Posterior dens with 5+5 spine-like chaetae. Distal part of the tibiotarsus subdivided; clavate tibiotarsal chaetae absent. Abd V and VI fused. Microsensillar body pattern 1,0/1,0,0,0 or 1,0/0,0,0,0. Species of *Mucrosomia* can be distinguished by the labral formula, the chaetotaxy of the postlabial (PostL) and labial triangle (LBT), the number of chaetae on the posterior furcal subcoxa, and the chaetotaxy of the collophore (lateral flaps and posterior side).

**Fig. 2:**
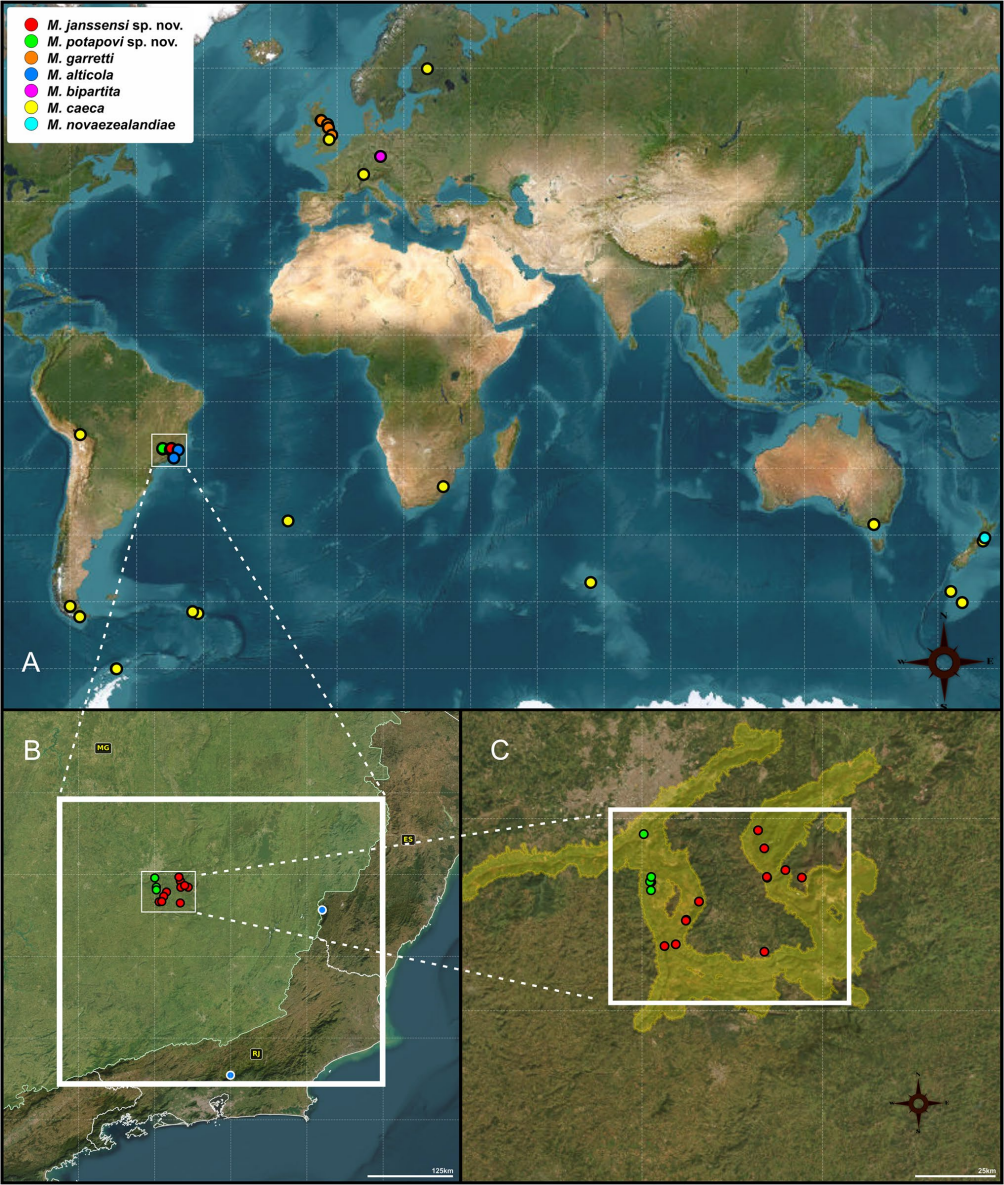
Distribution map of the genus *Mucrosomia*. (A) Global distribution; (B) distribution in Brazil; (C) distribution in the Quadrilátero Ferrífero, Minas Gerais, Brazil. See interactive version in Supplementary Material Interactive Map.html

All species of *Mucrosomia* were codified according to their original descriptions. Species distribution of the genus *Mucrosomia* as shown in Fig. 2.

**Habitat and distribution**. The genus is recorded from a wide range of environments, including montane forests at elevations above 2750 m (Mendonça and Queiroz 2013), estuarine mudflats with decayed bark and trunks encrusted in mud (Bagnall 1939), and groundwater from deep interstitial spaces exceeding 8 m in depth (Rusek 1946). It is also found in coastal zones of temperate and sub-Antarctic regions, occurring in leaf litter, under stones, and in areas associated with penguin colonies. Additional records come from insular environments, where individuals have been collected from leaf mold beneath Dracophyllum vegetation and other organic-rich substrates (Wahlgren 1906).

*Mucrosomia janssensi* **sp. nov.** Lima & Ferreira

zoobank.org:act:9AB7C47C-B9CF-4FE9-A1E9-590DF28E06AB

**Type material**. Holotype in slide (#24543/Coleção de Referência de Fauna do Solo, Universidade Estadual da Paraíba– CRFS/UEPB): Brazil, Minas Gerais state, Santa Barbara municipality, Parque Nacional Serra do Gandarela, AP14, 12 m deep, 20°01′49.6″S 43°40′41.9″W, 14.II-09.V.2012, Andrade coll. Paratypes metallized in stubs for SEM (#M04/CRFS/UEPB): 3, same data as holotype.

**Additional materials**. 10.15468/pbxmgz

**Distinctive characters**. Head with PAO not constricted; postlabial (PostL) with 5+5 chaetae; prelabral and labral formula 3/554; labial palp with 5 papillae (**A**–**E**) and hypostomal group with **H** and **h1**–**h2** very difficult to distinguish. Appendages with tibiotarsi I-III with 21, 21, 25 chaetae, respectively; collophore with lateral flaps (LF) 6+6 and posterior side (PS) with 5+5 chaetae. Posterior furcal subcoxa with 4+4 chaetae.

**Fig. 3:**
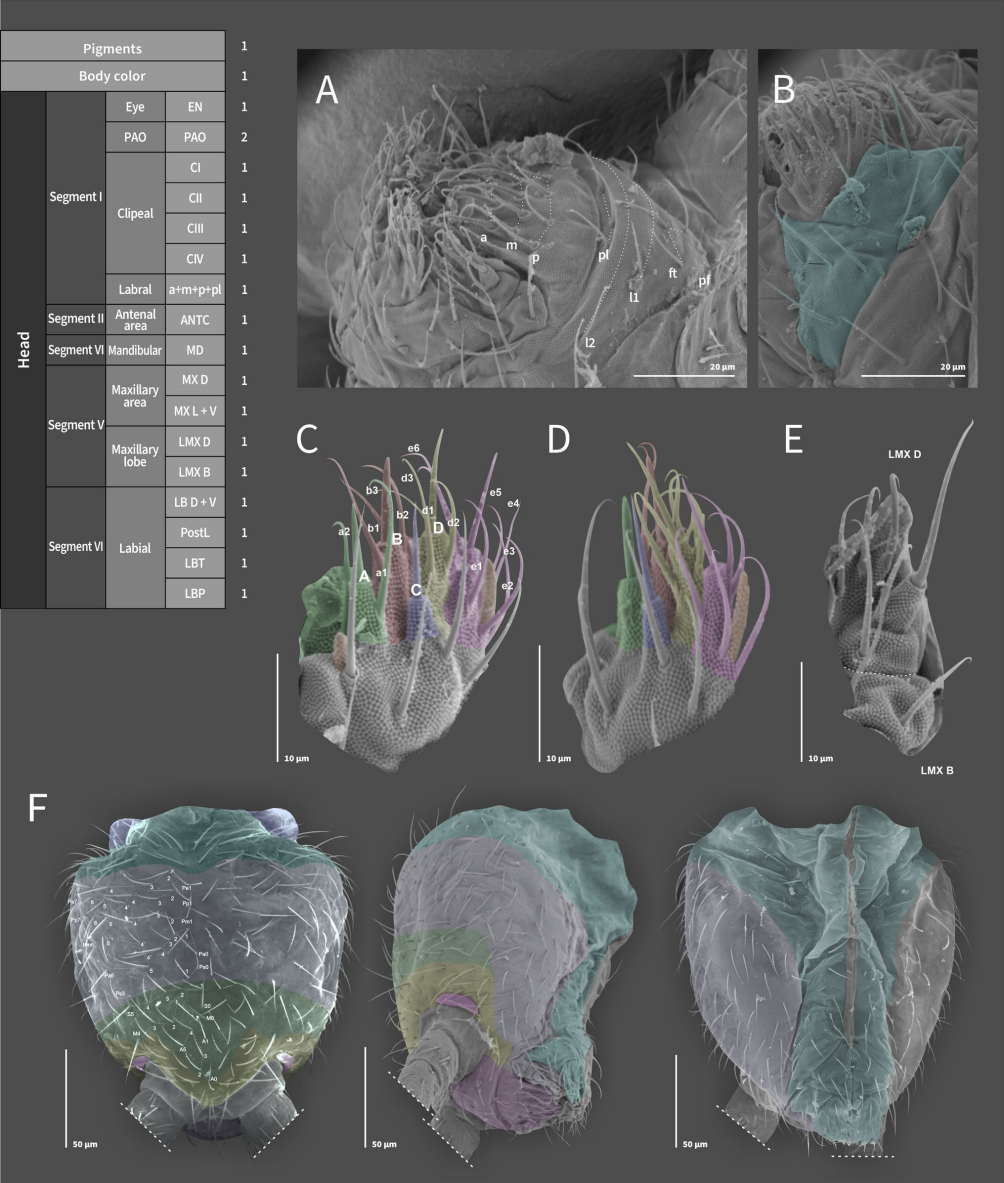
*Mucrosomia janssensi* **sp. n.** SEM images. Cephalic chaetotaxy and descriptive table. **A** Segment I: Clypeal and Labral areas. **B** Segment VI: Labial triangle. **C**, **D **Segment VI: Labial papilla and proximal chaetae. **E** Segment V: Maxillary lobe. **F** Dorsal and ventral views of the cephalic capsule

**Fig. 4:**
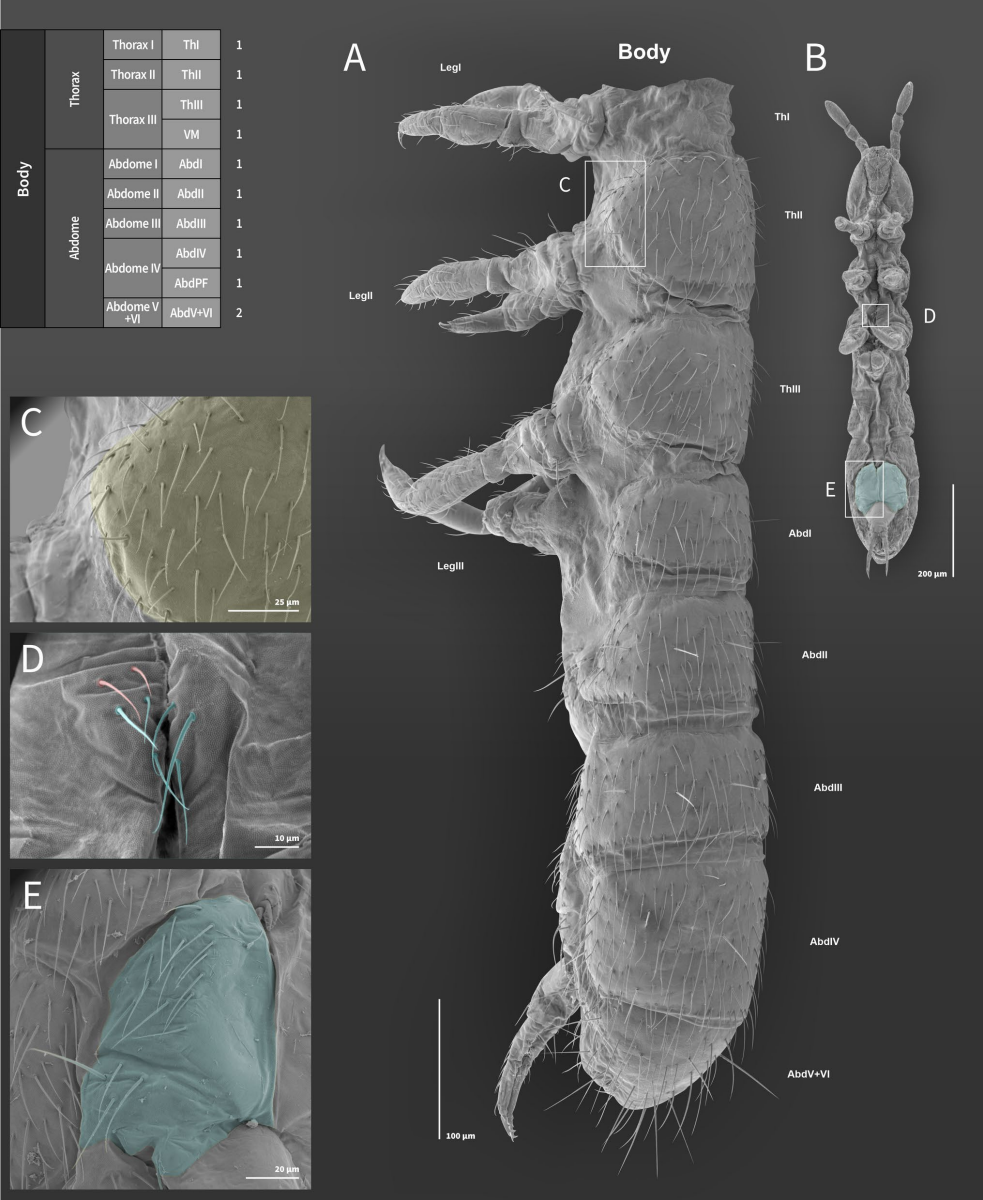
*Mucrosomia janssensi* **sp. n.** SEM images. Body chaetotaxy and descriptive table. **A** Body in lateral view. **B** Body in ventral view. **C** Thorax II. **D** Thorax III in the ventralmedial area between the meso and metacoxae. **E** Abdomen Parafurcal area (AbdPF)

**Fig. 5:**
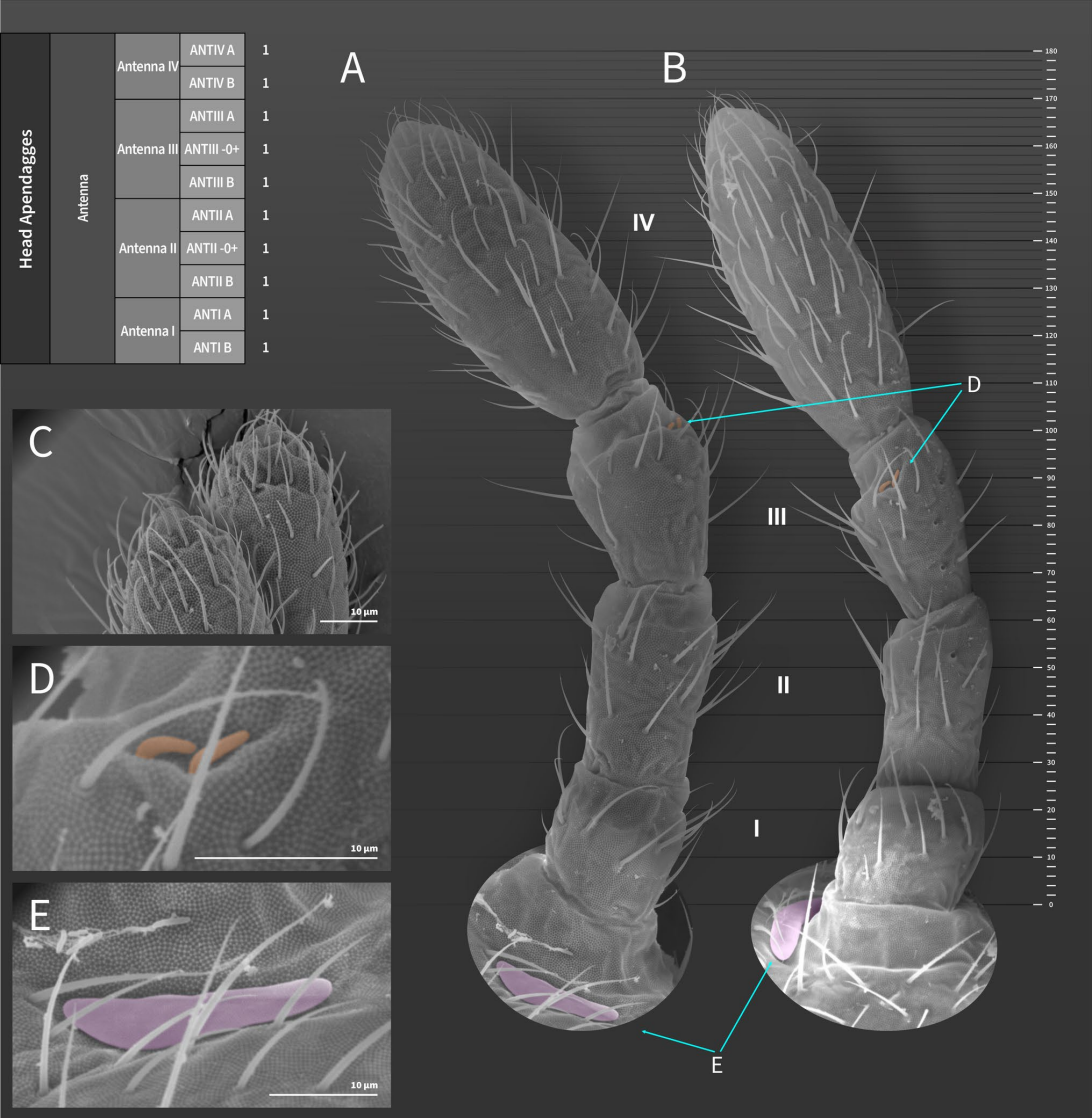
*Mucrosomia janssensi* **sp. n.** SEM images. Antennal chaetotaxy and descriptive table. **A**, **B** Entire antennal chaetotaxy. **C** Ant. IV apex ventral and dorsal views. **D** Detail of the sensilla of the Ant III apical organ. **E** Detail of the post-antennal organ

**Fig. 6:**
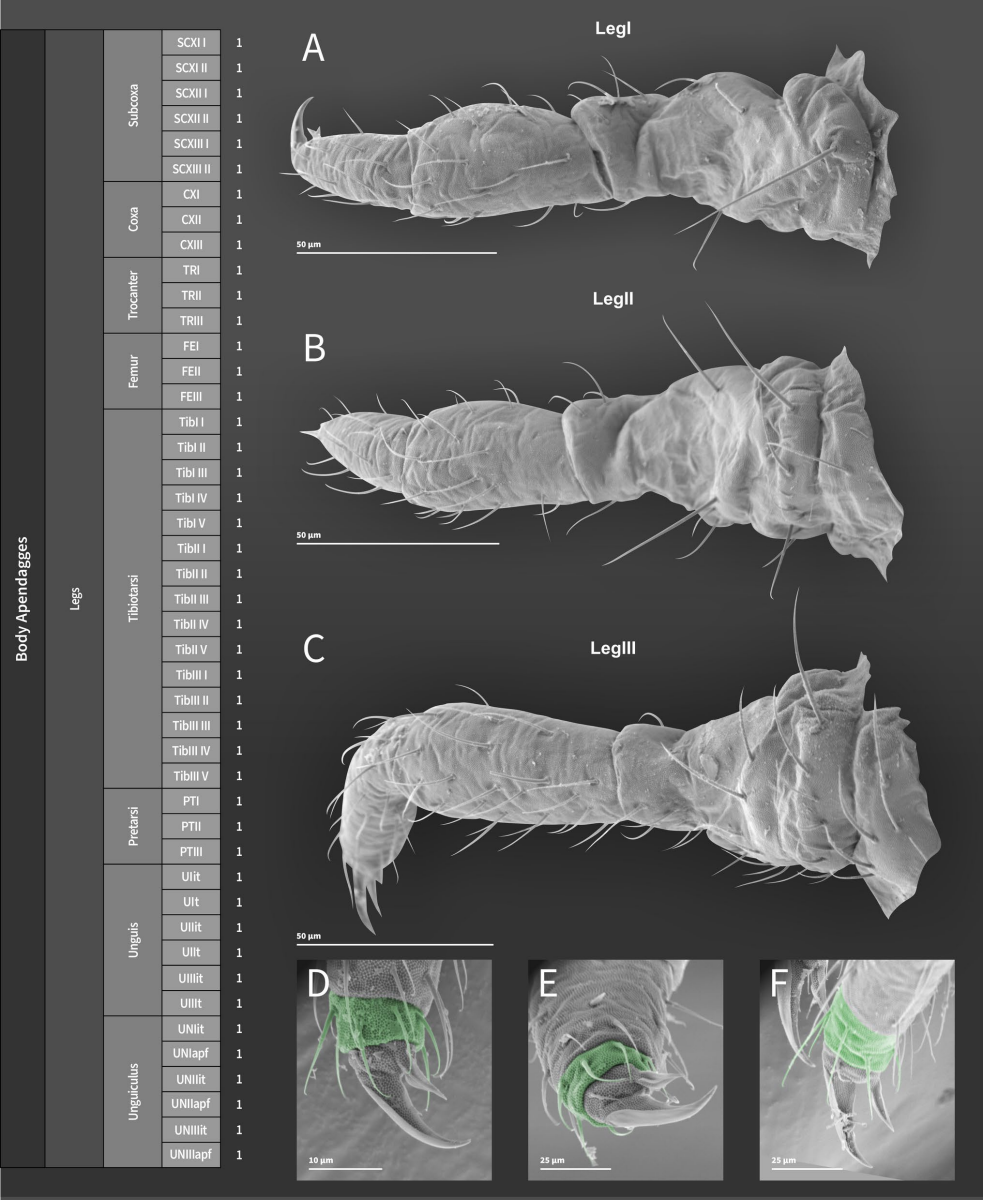
*Mucrosomia janssensi* **sp. n.** SEM images. Leg chaetotaxy and descriptive table. **A** Leg I. **B** Leg II. **C** Leg III. **D**, **E**, **F** Details of the apices of legs I–III

**Fig. 7:**
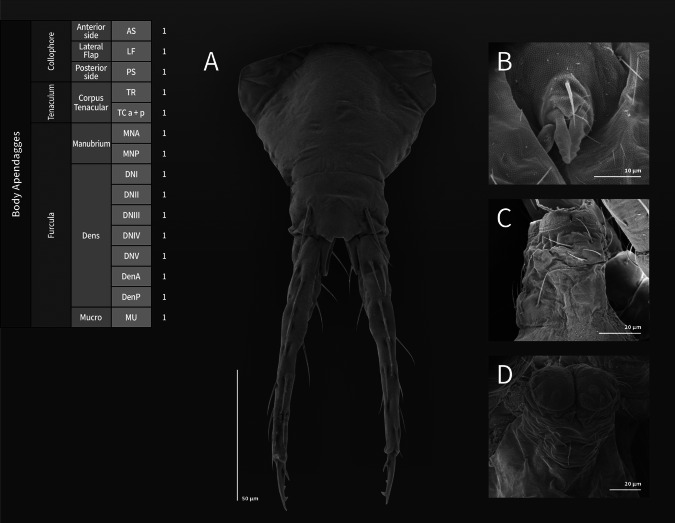
*Mucrosomia janssensi* **sp. n.** SEM images. Abdominal appendages chaetotaxy. **A** Furcula in anterior view. **B** Tenaculum in anterior view. **C** Collophore in lateral view. **D** Collophore in posterior view

**Coded description**. (Figs. 3, 4, 5, 6, and 7, Tables 1 and 2; in Supplementary Material see FreeDELTA Editor Mucrosomia database.dtz, Complete Description, Table S1 and Interactive Map.html).

**Table 2:** Comparison of main morphological characters among *Mucrosomia* species. “?” indicates unavailable data

	Species						
	*M. janssensi* **sp. nov.**	*M. potapovi* **sp. nov.**	*M. alticola*	*M. bipartita*	*M. caeca*	*M. garretti*	*M. novaezealandiae*
Type location	Brazil	Brazil	Brazil	Czech Republic	S. Georgia Island	Scotland	New Zealand
PAO	Not constricted	Not constricted	Constricted	Not constricted	Constricted	Constricted	Constricted
Prelabral and labral formula	3/554	4/554	4/554	3/554	3/554	3/554	?
Lobe maxillary distal area (LMX D)	6 + 6	7 + 7	7 + 7	6 + 6	?	?	?
Postlabial (PostL)	5 + 5	5 + 5	3 + 3–4 + 4	?	?	4–5	?
Body macrochaetae formula	1,1/3,3,3	1,1/3,3,3	1,1/1,3,2	1,1/3,3,3,4	?	1,1/3,3,3,4	?
Body macrosensillar formula	4,3/2,2,2,3,5	4,3/2,2,2,3,5	4,3/2,2,2,3,5	4,3/2,2,2,3,5	?	4,3/2,2,2,3,5	?
Body microsensillar formula	1,0/1,0,0,0,0	1,0/1,0,0,0,0	1,0/1,0,0,0,0	1,0/1,0,0,0,0	?	1,0/1,0,0,0,0	..,../0,..,..,..,.
Th. III ventral chaetae	3 + 3	3 + 3	1–3 + 1–3	0 + 0	0 + 0	0 + 0	0 + 0
Posterior furcal subcoxa chaetae	4 + 4	5 + 5	4 + 4	4 + 4	6 + 6	4 + 4	?
Tibiotarsus I chaetae	21	21	23–37	?	?	> 21	?
Tibiotarsus II chaetae	21	21	25–28	?	?	> 21	?
Tibiotarsus II chaetae	25	25	29–33	?	?	> 21 (sem T)	?
Unguis I–III inner tooth	+	+	+	-	+	+	-
Collophore lateral flaps chaetae	6 + 6	5 + 5	4 + 4–7 + 7	7 + 7	5 + 5 (4)	5–6 + 5–6	?
Dens anterior chaetae	10	10	9–11	10	10	10–12	10
Dens posterior chaetae	5	5	5	5	5	5	5

**Descriptive diagnosis**. Pigments (PI) absent. Eye Number (EN) 0+0. Post-antennal organ form (PAO) present entire. Labral formula (a+m+p+pl) 4+5+5+3 8(1)9(2). Lobe Maxillary distal area (LMX D) 6+6 2(1)8(6)2(7). Maxillary basal area (LMX B) 1+1 1(1). Labial chaetae, postlabial (PostL) 5+5 10(1). Labial triangle total chaetae (LBT) 9+9 18(1). Labial proximal chaetae and papillae chaetotaxy count (LBP) 26+26 8(1)8(3)2(4)2(5)32(6). Thorax I (ThI) 0+0. Thorax II (ThII) 6+6 2(8)8(9)2(10). Thorax III (ThIII) 4+4 2(8)6(9). Thorax III area ventralmedial (VM) 3+3 4(1)2(8). Abdomen I (AbdI) 6+6 6(8)4(9)2(10). Abdomen II (AbdII) 5+5 6(8)4(9). Abdomen III (AbdIII) 5+5 6(8)4(9). Abdomen IV (AbdIV) 7+7 8(8)6(9). Abdomen IV area Abdomen Parafurcal region (AbdPF) 19+19 36(1)2(8). Abdomen V and VI sensillar count (AbdV+VI) 5+5 10(9). Antenna I whorl A total chaetae (ANTI A) 15 12(1)2(12). Antenna I whorl B total chaetae (ANTI B) 2 2(11). Antenna II whorl A total chaetae (ANTII A) 8 7(1)1(12). Antenna II whorls −I, 0, and +I total chaetae (ANTI −0+) 12 12(1). Antenna II whorl B total chaetae (ANTII B) 5 3(1)2(11). Antenna III whorl A total chaetae (ANTIII A) 9 4(1)2(12)2(13)1(14). Antenna III whorls −I, 0, and +I total chaetae (ANTIII −0+) 12 12(1). Antenna III whorl B total chaetae (ANTIII B) 2 1(1)1(11). Antenna IV whorl A total chaetae (ANTIV A) 65 44(1)5(11)10(9)5(12)1(15). Antenna IV whorl B total chaetae (ANTIV B) 8 8(1). Tibiotarsus I whorl I total chaetae (TibI I) 7 7(1). Tibiotarsus I whorl II total chaetae (TibI II) 7 7(1). Tibiotarsus I whorl III total chaetae (TibI III) 7 7(1). Tibiotarsus I whorl IV (TibI IV) absent. Tibiotarsus I whorl V (TibI V) absent. Tibiotarsus II whorl I total chaetae (TibII I) 7 7(1). Tibiotarsus II whorl II total chaetae (TibII II) 7 7(1). Tibiotarsus II whorl III total chaetae (TibII III) 7 7(1). Tibiotarsus II whorl IV (TibII IV) absent. Tibiotarsus II whorl V (TibII V) absent. Tibiotarsus III whorl I total chaetae (TibIII I) 12 12(1). Tibiotarsus III whorl II total chaetae (TibIII II) 7 7(1). Tibiotarsus III whorl III total chaetae (TibIII III) 7 7(1). Tibiotarsus III whorl IV (TibIII IV) absent. Tibiotarsus III whorl V (TibIII V) absent. Unguis I inner tooth (UIit) present. Unguis II inner tooth (UIIit) present. Unguis III inner tooth (UIIIit) present. Anterior side total chaetae (AS) 0. Lateral flap total chaetae (LF) 6+6 12(1). Posterior side total chaetae (PS) 5+5 10(1). Tenaculum rami (TR) 4+4 teeth. Tenaculum Corpus (a+p) (TC a+p) 1 1(1). Anterior manubrium total chaetae (MNA) 1+1 2(16). Posterior manubrium total chaetae (NMP) 9+9 18(1). Dental anterior total chaetae (DenA) 10+10 20(17). Dental posterior total chaetae (DenP) 5+5 8(1) 2(17). Mucro lamellae total teeth (MU) 5.

**Habitat and distribution**. *Mucrosomia janssensi* **sp. n.** was found in the Quadrilátero Ferrífero, Minas Gerais, in areas of the Serra do Gandarela and near the Serra da Moeda, with fewer than 15 records of this species.

**Remarks**. The new species resembles *M. bipartita* and *M. garretti* by having a prelabral and labral formula of 3/554 and 4+4 chaetae on the posterior furcal subcoxa. It differs, however, in the number of chaetae on the ventral side of Th. III ventral: 3+3 in new species and 0+0 chaetae in *M. bipartita* and *M. garretti*. The three species differ in the chaetotaxy of the collophore (see Table 2 and Supplementary Material).

**Etymology**. This species is named in memory of Frans Janssens (1952–2024), in recognition of his significant contribution to the study of Collembola.

*Mucrosomia potapovi* **sp. nov.** Ferreira & Lima

zoobank.org:act:021C84D7-542C-4332-AE17-7B44EF84F7EA

**Type material**. Holotype in slide (#16082/CRFS/UEPB): Brazil, Minas Gerais state, Nova Lima municipality, 0002 MS, 20°11′09.1″S 43°58′28.0″W, 09.X.2017, Carste Team coll. Paratypes in slides (#16080, #16081/CRFS/UEPB): same data as holotype.

**Additional materials**. 10.15468/pbxmgz

**Distinctive characters.** Head with PAO not constricted; postlabial (PostL) with 5+5 chaetae; prelabral and labral formula 4/554; labial palp with 5 papillae (**A**–**E**) and hypostomal group with **H** and **h1**–**h2** very difficult to distinguish. Appendages with tibiotarsi I–III with 21, 21, 25 chaetae, respectively; collophore with lateral flaps (LF) 5+5 and posterior side (PS) with 4+4 chaetae. Posterior furcal subcoxa with 5+5 chaetae.

**Fig. 8:**
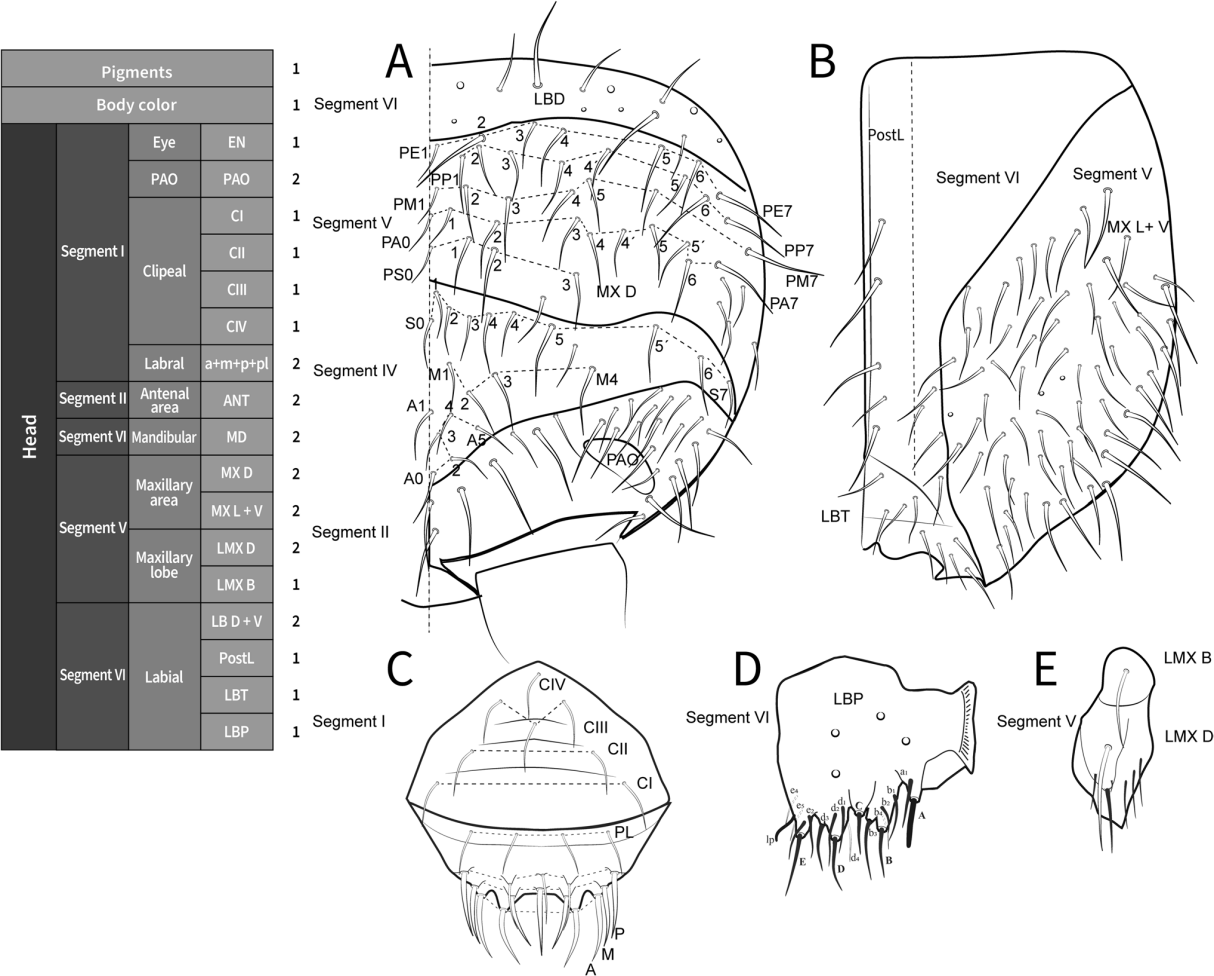
*Mucrosomia potapovi* **sp. n.** Cephalic chaetotaxy and descriptive table. **A** Dorsal cephalic schematic chaetotaxy. **B** Labial triangle, medial and distal chaetotaxy. **C** Clypeal and labral chaetotaxy. **D** Labial proximal chaetotaxy. **E** Maxillary lobe

**Fig. 9:**
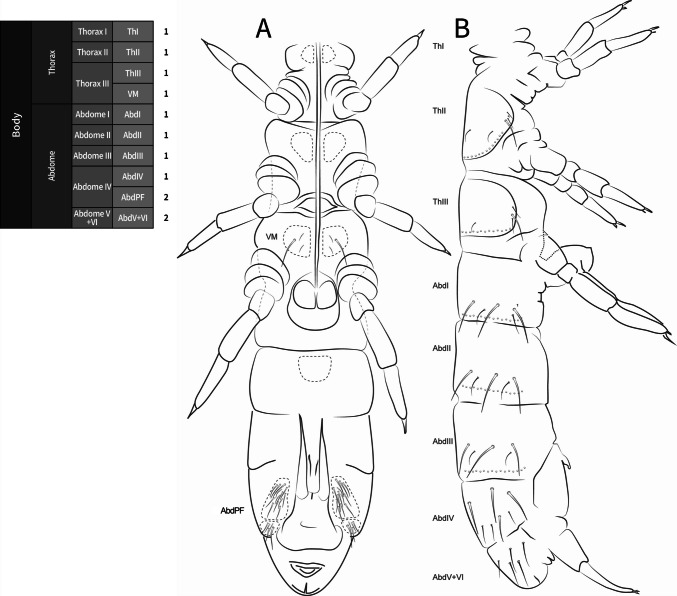
*Mucrosomia potapovi* **sp. n.** Body chaetotaxy and descriptive table. **A** Body in ventral view. **B** Body in dorsal view

**Fig. 10:**
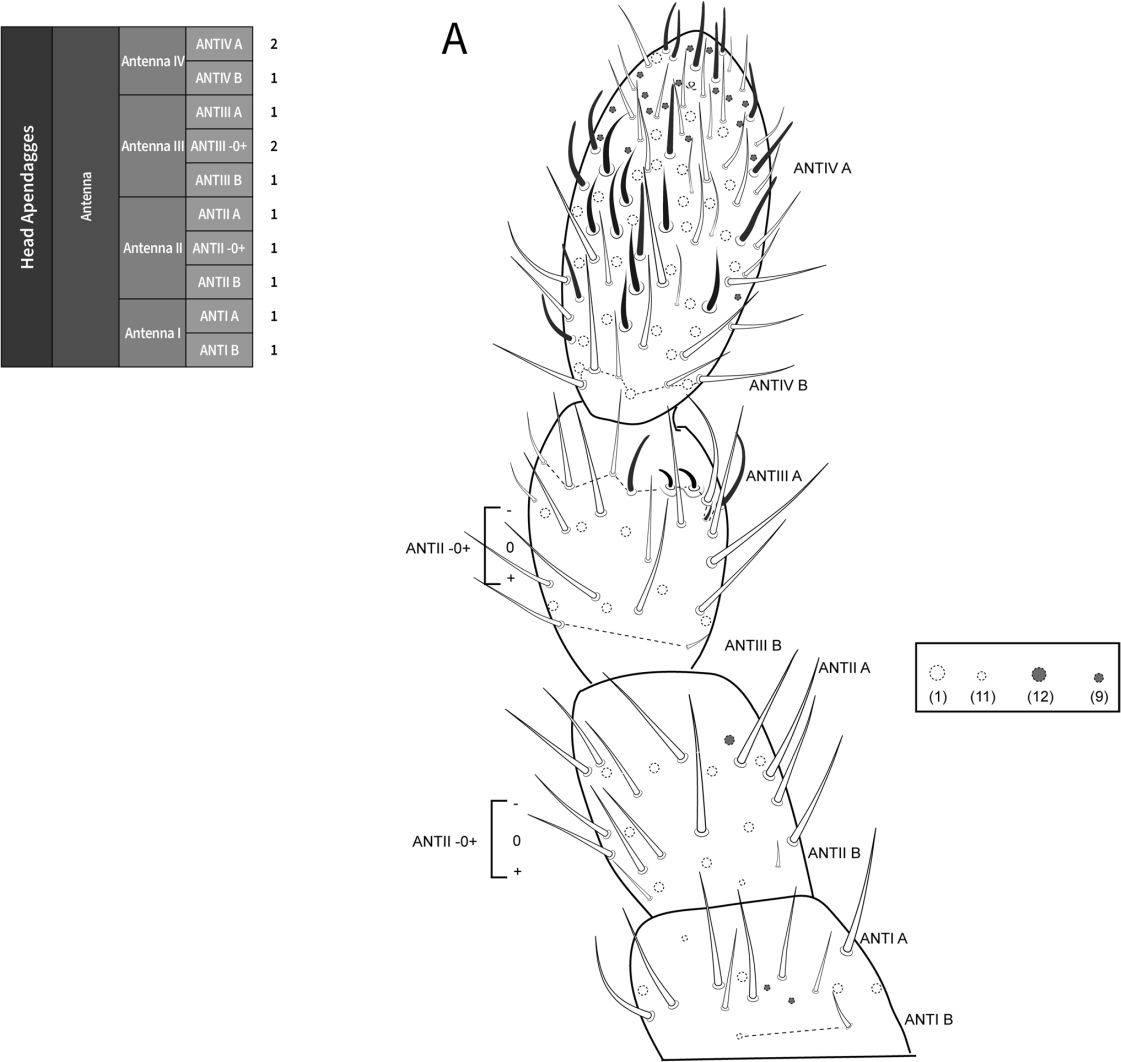
*Mucrosomia potapovi* **sp. n.** Antennal chaetotaxy and descriptive table. **A** Entire antennal chaetotaxy. Chaetal types (1), (9), (11), and (12) follow those illustrated in the chaetal bank (see Fig. 1)

**Fig. 11:**
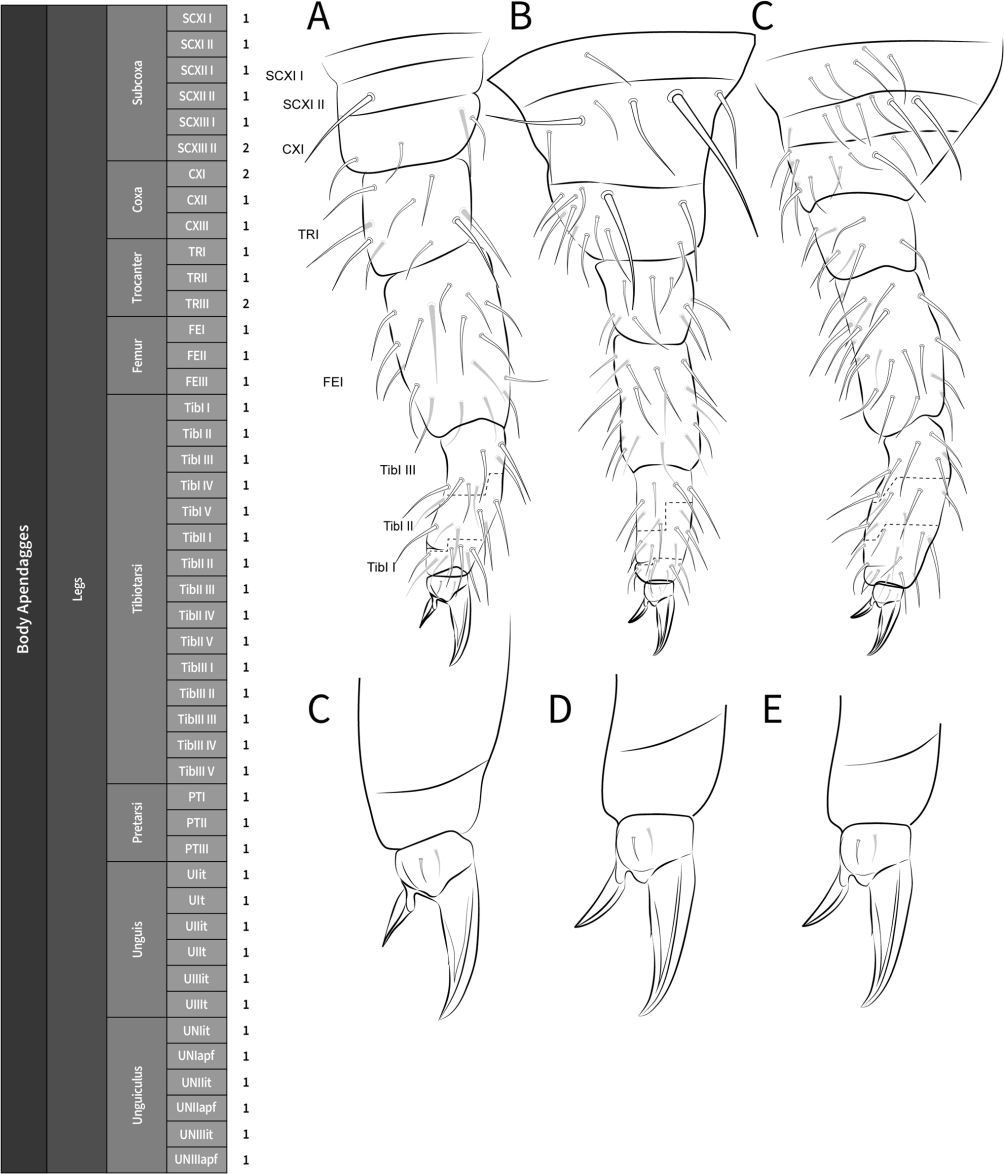
*Mucrosomia potapovi* **sp. n.** Leg chaetotaxy and descriptive table. **A **Leg I. **B** Leg II. **C** Leg III. **D**, **E**, **F** Details of the apices of legs I–III

**Fig. 12:**
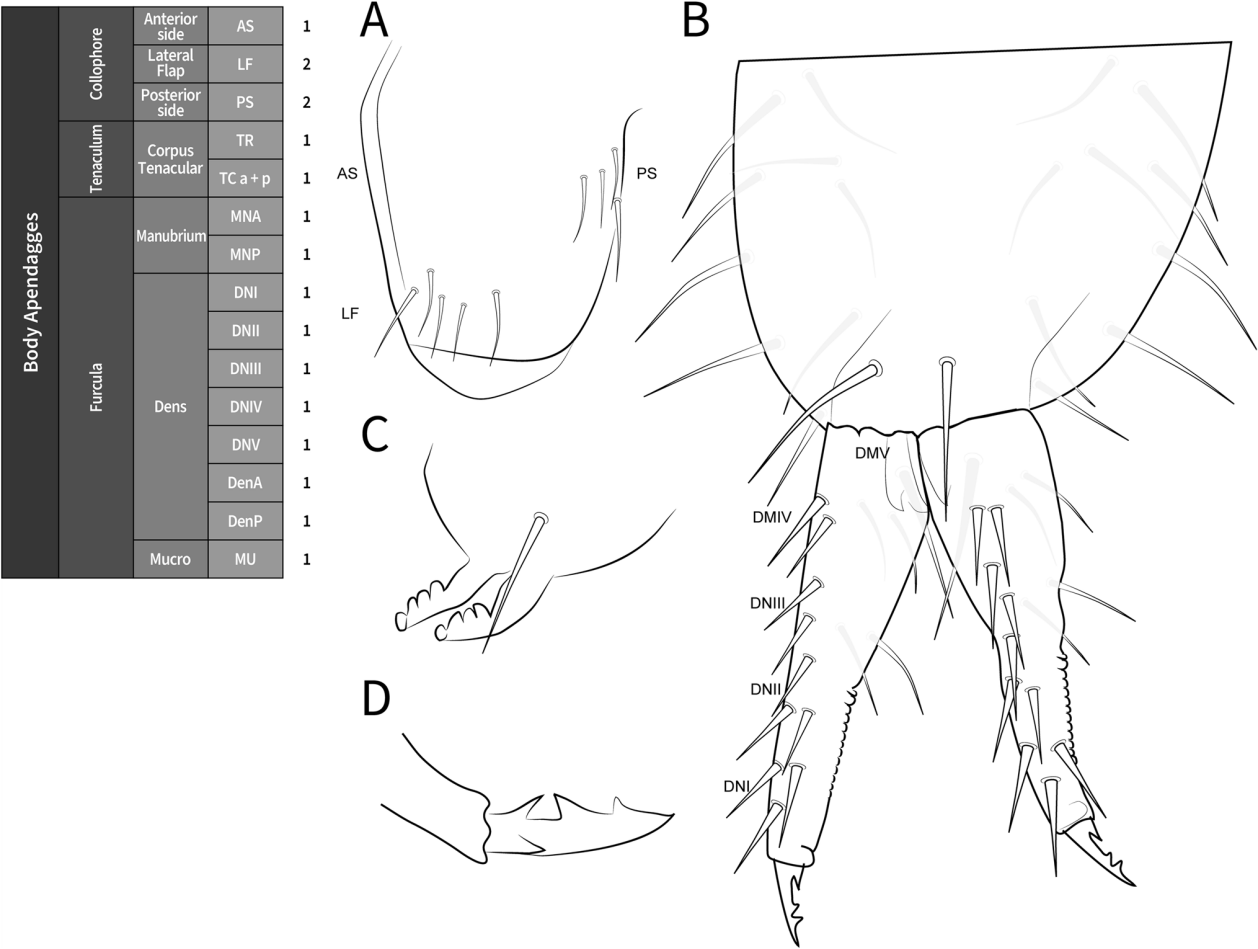
*Mucrosomia potapovi* **sp. n.** Abdominal appendages chaetotaxy. **A** Collophore: anterior side (AS), lateral flap (LF), and posterior side (PS). **B** Furcula. **C** Tenaculum in anterior view. **D** Mucro. The lighter chaetae are located on the posterior side

**Coded description**. (Figs. 8, 9, 10, 11, and 12, Tables 1 and 2; in Supplementary Material see FreeDELTA Editor Mucrosomia database.dtz, Complete Description, Table S1 and Interactive Map.html)

**Descriptive diagnosis**. Pigments (PI) absent. Eye Number (EN) 0+0. Post-antennal organ form (PAO) present entire. Labral formula (a+m+p+pl) 4+5+5+3 8(1)9(2). Lobe Maxillary distal area (LMX D) 6+6 2(1)8(6)2(7). Maxillary basal area (LMX B) 1+1 1(1). Labial chaetae, postlabial (PostL) 5+5 10(1). Labial triangle total chaetae (LBT) 9+9 18(1). Labial proximal chaetae and papillae chaetotaxy count (LBP) 26+26 8(1)8(3)2(4)2(5)32(6). Thorax I (ThI) 0+0. Thorax II (ThII) 6+6 2(8)8(9)2(10). Thorax III (ThIII) 4+4 2(8)6(9). Thorax III area ventralmedial (VM) 3+3 4(1)2(8). Abdomen I (AbdI) 6 6 6(8)4(9)2(10). Abdomen II (AbdII) 5+5 6(8)4(9). Abdomen III (AbdIII) 5+5 6(8)4(9). Abdomen IV (AbdIV) 7+7 8(8)6(9). Abdomen Parafurcal region (AbdPF) 19+19 36(1)2(8). Abdomen V and VI sensillar count (AbdV+VI) 5+5 10(9). Antenna I whorl A total chaetae (ANTI A) 15 12(1)2(12). Antenna I whorl B total chaetae (ANTI B) 2 2(11). Antenna II whorl A total chaetae (ANTII A) 8 7(1)1(12). Antenna II whorls −I, 0, and +I total chaetae (ANTI −0+) 12 12(1). Antenna II whorl B total chaetae (ANTII B) 5 3(1)2(11). Antenna III whorl A total chaetae (ANTIII A) 9 4(1)2(12)2(13)1(14). Antenna III whorls −I, 0, and +I total chaetae (ANTIII −0+) 12 12(1). Antenna III whorl B total chaetae (ANTIII B) 2 1(1)1(11). Antenna IV whorl A total chaetae (ANTIV A) 65 44(1)5(11)10(9)5(12)1(15). Antenna IV whorl B total chaetae (ANTIV B) 8 8(1). Tibiotarsus I whorl I total chaetae (TibI I) 7 7(1). Tibiotarsus I whorl II total chaetae (TibI II) 7 7(1). Tibiotarsus I whorl III total chaetae (TibI III) 7 7(1). Tibiotarsus I whorl IV (TibI IV) absent. Tibiotarsus I whorl V (TibI V) absent. Tibiotarsus II whorl I total chaetae (TibII I) 7 7(1). Tibiotarsus II whorl II total chaetae (TibII II) 7 7(1). Tibiotarsus II whorl III total chaetae (TibII III) 7 7(1). Tibiotarsus II whorl IV (TibII IV) absent. Tibiotarsus II whorl V (TibII V) absent. Tibiotarsus III whorl I total chaetae (TibIII I) 12 12(1). Tibiotarsus III whorl II total chaetae (TibIII II) 7 7(1). Tibiotarsus III whorl III total chaetae (TibIII III) 7 7(1). Tibiotarsus III whorl IV (TibIII IV) absent. Tibiotarsus III whorl V (TibIII V) absent. Unguis I inner tooth (UIit) present. Unguis II inner tooth (UIIit) present. Unguis III inner tooth (UIIIit) present. Anterior side total chaetae (AS) 0. Lateral flap total chaetae (LF) 6+6 12(1). Posterior side total chaetae (PS) 5+5 10(1). Tenaculum rami (TR) 4+4 teeth. Tenaculum Corpus (a+p) (TC a+p) 1 1(1). Anterior manubrium total chaetae (MNA) 1+1 2(16). Posterior manubrium total chaetae (NMP) 9+9 18(1). Dental anterior total chaetae (DenA) 10+10 20(17). Dental posterior total chaetae (DenP) 5+5 8(1) 2(17). Mucro lamellae total teeth (MU) 5.

**Habitat and distribution**. *Mucrosomia potapovi* **sp. n.** was found in the Quadrilátero Ferrífero, Minas Gerais, in the area of the Serra da Moeda, with very low abundance, there are only five records of this species.

**Remarks**. The new species resembles *M. alticola* by having a labral formula 4/554 and 3+3 chaetae on the ventral side of Th. III. It differs in the postlabial (PostL), with 5+5 in *M. potapovi *sp. nov. and 3+3 in *M. alticola;* in the posterior furcal subcoxa with 5+5 chaetae in the new species and 4+4 in *M. alticola*; and by having fewer chaetae on the tibiotarsi I–III (23–37, 25–28, 29–33 in *M. alticola*, 21, 21, 25 in *M. potapovi* **sp. n.**; Table 2). The three Brazilian species, *M. janssensi* **sp. n.**, *M. potapovi* **sp. n.**, and *M. alticola* differ from one another in the chaetotaxy of the collophore, which is distinct in each species (see Table 2 and Supplementary Material).

**Etymology**. This species is named in recognition of the outstanding contributions of Dr. Mikhail Potapov to the study of Collembola, with particular emphasis on the family Isotomidae.

*Mucrosomia alticola* Mendonça & Queiroz 2013 (Tables 1 and 2; in Supplementary Material see FreeDELTA Editor Mucrosomia database.dtz, Figs. S1–S5, Complete Description, Coded Description, Table S1 and Interactive Map.html)

**Habitat and distribution**. The type locality is Brazil, Rio de Janeiro State, Teresópolis: Parque Nacional da Serra dos Órgãos (PARNASO), and Minas Gerais State: Parque Nacional do Caparaó (PNC), at altitudes of approximately 1400 m and 2750 m, respectively (Mendonça & Queiroz 2013).

*Mucrosomia bipartita* (Rusek, 1996) (Tables 1 and 2; in Supplementary Material see FreeDELTA Editor Mucrosomia database.dtz, Figs. S6–S10, Complete Description, Coded Description, Table S1 and Interactive Map.html)

**Habitat and distribution**. The type locality is Czech Republic, Western Bohemia, Sokolov: Groundwater, deep interstitial spaces, hydraulic drill core at 8.2 m depth (Rusek, 1996).

*Mucrosomia caeca* (Wahlgren 1906) (Tables 1 and 2; in Supplementary Material see FreeDELTA Editor Mucrosomia database.dtz, Figs. S11–S15, Complete Description, Coded Description, Table S1 and Interactive Map.html)

**Habitat and distribution**. The type locality is in South Georgia Island, near the Antarctic continent. South Georgia, Cumberland Bay (Jason Harbor): coastal environment.

(= *Folsomia fimetaroides* Womersley, 1934)—Australia, Victoria, Sherbrook, Sassafras: leaf litter in forest habitats.

(= *Folsomia lunata* Salmon, 1943)—New Zealand, Wellington, Johnston’s Hill, Karori: leaf litter. Widely distributed, circumpolar: South Georgia Islands, South Shetland Islands, Tristan da Cunha, Kerguelen, Auckland, Campbell, Argentina, Chile, Bolivia, Peru, South Africa. Habitats include coastal environments, leaf litter, under stones, and penguin colonies.

(= *Parafolsomia litorea* Salmon, 1949)—Campbell Island, southern coast below Mount Dumas: leaf mold beneath Dracophyllum, under stones in penguin colonies.

(= *Folsomia pusilla* Salmon, 1944)—New Zealand, Bold Peak, at an altitude of approximately 3000 m, Lake Wakatipu: in leaf mold in beech forest.

*Mucrosomia garretti* (Bagnall 1939) (Tables 1 and 2; in Supplementary Material see FreeDELTA Editor Mucrosomia database.dtz, Figs. S16–S20, Complete Description, Coded Description, Table S1 and Interactive Map.html)

**Habitat and distribution**. The type locality is in Scotland and England, including Northumberland, Durham, and Yorkshire (Alnmouth, Ryhope Dene, Ferriby, etc.), where the species occurs in estuarine mud under encrusted trunks, decayed bark, and along the margins of the Humber River (Bagnall 1939).

(= *Isotomina caeca* Gisin, 1960) Corby, Northants (Lincolnshire, Central England), in prairie soil near iron mines; Bahia, Palmer Peninsula (Antarctica).

(= *Cryptopygus coecus* Massoud & Rapoport, 1968) several localities in Argentinian Patagonia, such as Lake Victoria (Nahuel Huapi), Lake Frías, Puerto Blest, Lake Currhué, and Lake Lácar.

*Mucrosomia novaezealandiae* (Salmon, 1943) (Tables 1 and 2; in Supplementary Material see FreeDELTA Editor Mucrosomia database.dtz, Figs. S21–S25, Complete Description, Coded Description, Table S1 and Interactive Map.html)

**Habitat and distribution**. (= *Folsomia novaezealandiae* Salmon, 1943; = *Spinurosomia novaezealandiae* (Salmon, 1943); = *Parafolsomia novaezealandiae* (Salmon, 1943); = *Cryptopygus novaezealandiae* (Salmon, 1943)). The type locality is in New Zealand, Buller's Bush, Levin, in leaf Mould (Potapov and Janion-Scheepers 2017).

## Key to species of the genus *Mucrosomia*

1 Abd I ms absent..............................................................*M. novaezealandiae*

1 Abd I ms present..............................................................2

2 (1) Labral formula 3/554..............................................................3

2 Labral formula 4/554..............................................................6

3(2)Posterior furcal subcoxa chaetae 6+6..............................................................*M. caeca*

3 Posterior furcal subcoxa chaetae with fewer than6+6..............................................................4

4 (3) Chaetae along ventral line of Th. III absent..............................................................5

4 Chaetae along ventral line of Th. III present (3+3); Collophore with lateral flaps 6+6 and posterior side with 5+5 chaetae..............................................................*M. janssensi* **sp. nov.**

5(4) Labial triangle with 9+9 chaetae (basomedian 4+4 and basolateral 5+5); Unguis I–III without inner tooth; Collophore with lateral flaps 7+7 chaetae..............................................................*M. bipartita*

5 Labial triangle with 10+10 chaetae (basomedian 5+5 and basolateral 5+5); Unguis I–III with inner tooth; Collophore with lateral flaps 5–6+5–6 chaetae..............................................................*M. garretti*

6(2) Labial chaetae along the ventral groove 4+4; Chaetae on tibiotarsi I–III: 23–37, 25–28, 29–33; Posterior furcal subcoxa with 4+4 chaetae..............................................................*M. alticola*

6 Labial chaetae along the ventral groove 5+5; Chaetae on tibiotarsi I–III: 21, 21, 25; Posterior furcal subcoxa with 5+5 chaetae..............................................................*M. potapovi* **sp. nov**.

## Discussion

The taxonomy of Isotomidae has long been hindered by extensive morphological convergence and character reduction, particularly among blind and edaphic lineages that frequently exhibit highly conservative external traits (Arbea and Kahrarian 2015). Subtle differences in chaetotaxy, trichobothrial patterns, and furcal morphology are often the only reliable traits for species delimitation, requiring specialized expertise and detailed microscopic analyses (Khormandichali et al. 2024). These limitations are exemplified by *Mucrosomia*, which, as noted by Potapov and Janion-Scheepers (2017), has changed its generic position several times throughout its taxonomic history, reflecting the persistent uncertainty caused by overlapping and ambiguously defined morphological characters. Our results reinforce these challenges, revealing that several previously described species displays overlapping and inconsistently reported diagnostic features.

By applying Coded Taxonomy (Zeppelini et al. 2024), we systematically reviewed all valid species of *Mucrosomia* based on published descriptions and available literature, and established standardized character codifications without direct examination of type material. This approach reduces subjectivity and enables direct comparability across taxa, facilitating the detection of subtle but consistent diagnostic differences. Variations in chaetotaxy of the postlabial (PostL), collophore, and posterior furcal subcoxa—characters often overlooked or inconsistently reported—proved decisive for separating closely related taxa such as *M. janssensi* sp. nov., *M. potapovi* sp. nov., and *M. alticola*. Moreover, the CT framework generates a digital-ready dataset that can be incorporated into interactive keys and machine learning applications, expanding the potential for semi-automated species identification.

Both *Mucrosomia janssensi* sp. nov. and *M. potapovi* sp. nov. exhibit highly restricted distributions within the ferruginous montane ecosystems of the Quadrilátero Ferrífero, Minas Gerais, contrasting sharply with the broader or even circumpolar ranges of congeners such as *M. caeca*, *M. garretti*, and *M. novaezealandiae*. In comparison, *M. alticola* shows an intermediate Neotropical montane pattern suggesting orographic isolation and climatic filtering rather than dependence on ferruginous substrates. While the new Brazilian species appear microendemic to ironstone outcrops and associated subterranean microhabitats, *M. alticola* is epigeic in leaf litter, and the widespread congeners inhabit coastal, estuarine, and sub-Antarctic environments. This gradient of range sizes and habitat affiliations within *Mucrosomia* likely reflects distinct geomorphological drivers and ecological specializations, which future molecular analyses may help clarify, particularly regarding species delimitation and phylogeographic patterns.

## Supplementary Information

Below is the link to the electronic supplementary material.

ESM 1 (DOCX 6.92 MB)

## Data Availability

All data generated or analyzed during this study are included in this published article and its Supplementary Information files, including the complete coded dataset and species descriptions generated from the FreeDELTA database.
