# Supplementary material for: Coded Taxonomy Applied to Old and New Descriptions of Mucrosomia (Collembola: Isotomidae): A Bibliographic Revision and New Species of the Genus
Source: Neotrop Entomol. 2026 May 28;55(1):55. doi: 10.1007/s13744-026-01397-4 (PMC13219218; doi:10.1007/s13744-026-01397-4)
Supplement: Supplementary file 1 — (DOCX 6.92 MB) [file 13744_2026_1397_MOESM1_ESM.docx]

**Supplementary Material**

**Coded Taxonomy Applied to Old and New Descriptions of *Mucrosomia* (Collembola: Isotomidae): a Bibliographic Revision and New Species of the Genus**

zoobank.org:pub:A547F653-9CA5-4EF3-8063-267F66422442

Estevam Cipriano Araujo de Lima^1*^ Aila Soares Ferreira^1^ Misael Augusto de Oliveira-Neto^1,3,4^ Bruna Carolline Honório Lopes^1^ Nathan Paiva Brito^1,2^ Roniere Andrade de Brito^1^ & Douglas Zeppelini^1-2^

¹ Laboratório de Sistemática de Collembola e Conservação, Instituto de Biologia do Solo, Universidade Estadual da Paraíba, João Pessoa, PB, Brasil

² Programa de Pós-Graduação em Ciências Biológicas – Zoologia, Universidade Federal da Paraíba, João Pessoa, PB, Brazil

³ Laboratório de Estudos Subterrâneos, Departamento de Ecologia e Biologia Evolutiva, Universidade Federal de São Carlos, São Carlos, SP, Brasil

⁴ Programa de Pós-Graduação em Ecologia e Recursos Naturais, Universidade Federal de São Carlos, São Carlos, SP, Brasil

**FreeDELTA Editor.**

To access this dataset, first download and install the FreeDELTA Editor from the DELTA website https://www.delta-intkey.com/www/programs.htm. Once the software is installed, the *Mucrosomia* database can be accessed by double-clicking the button below.

Click here

***Mucrosomia janssensi* sp. nov. Lima & Ferreira**

**Complete Description:** Pigments (PI) absent. Body color (BC) White. Eye Number (EN) 0+0. Post-antennal organ form (PAO) present entire. Clypeal area (CI) 1+1 2(1). Clypeal area (CII) 1+1 2(1). Clypeal area (CIII) 1 1(1). Clypeal area (CIV) 1+1+1 3(1). Labral formula (a+m+p+pl) 4+5+5+3 8(1)9(2). Anntenal area chaetotaxy (ANT) 17+2+17 36(1). Mandibular area chaetotaxy (MD) 15+3+15 33(1). Maxilar area chaetotaxy dorsal (MX D) 29+2+29 60(1). Maxilar area chaetotaxy dorsal lateral and ventral (MX L+V) 52+52 100(1). Lobe Maxillary distal area (LMX D) 6+6 2(1)8(6)2(7). Lobe Maxillary basal area (LMX B) 1+1 1(1). Labial area chaetotaxy dorsal and ventral (LB D+V) 20+20 40(1). Postlabial (PostL) 5+5 10(1). Labial triangle total chaetae (LBT) 9+9 18(1). Labial proximal chaetae and papillae chaetotaxy count (LBP) 26+26 8(1)8(3)2(4)2(5)32(6). Thorax I (ThI) 0+0. Thorax II (ThII) 6+6 2(8)8(9)2(10). Thorax III (ThIII) 4+4 2(8)6(9). Thorax III area ventralmedial (VM) 3+3 4(1)2(8). Abdomen I (AbdI) 6+6 6(8)4(9)2(10). Abdomen II (AbdII) 5+5 6(8)4(9). Abdomen III (AbdIII) 5+5 6(8)4(9). Abdomen IV (AbdIV) 7+7 8(8)6(9). Abdomen IV area BPIV L + M total chaetae (BPIV L + M) 19+19 36(1)2(8). Abdomen V and VI sensillar count (AbdV+VI) 5+5 10(9). Antenna I whorl A total chaetae (ANTI A) 15 12(1)2(12). Antenna I whorl B total chaetae (ANTI B) 2 2(11). Antenna II whorl A total chaetae (ANTII A) 8 7(1)1(12). Antenna II whorls -I, 0 and +I total chaetae (ANTI -0+) 12 12(1). Antenna II whorl B total chaetae (ANTII B) 5 3(1)2(11). Antenna III whorl A total chaetae (ANTIII A) 9 4(1)2(12)2(13)1(14). Antenna III whorls -I, 0 and +I total chaetae (ANTIII -0+) 12 12(1). Antenna III whorl B total chaetae (ANTIII B) 2 1(1)1(11). Antenna IV A total chaetae (ANTIV A) 65 44(1)5(11)10(9)5(12)1(15). Antenna IV B total chaetae (ANTIV B) 8 8(1). Subcoxa I area I total chaetae (SCXI I) 0. Subcoxa I area II total chaetae (SCXI II) 1 1(1). Subcoxa II area I total chaetae (SCXII I) 1 1(1). Subcoxa II area II total chaetae (SCXII II) 6 6(1). Subcoxa III area I total chaetae (SCXIII I) 5 5(1). Subcoxa III area II total chaetae (SCXIII II) 8 8(1). Coxa I total chaetae (CXI) 3 3(1). Coxa II total chaetae (CXII) 12 12(1). Coxa III total chaetae (CXIII) 11 11(1). Trochanter I total chaetae (TRI) 9 9(1). Trochanter II total chaetae (TRII) 9 9(1). Trochanter III total chaetae (TRIII) 10 10(1). Femur I total chaetae (FEI) 17 17(1). Femur II total chaetae (FEII) 18 18(1). Femur III total chaetae (FEIII) 21 21(1). Tibiotarsus I whorl I total chaetae (TibI I) 7 7(1). Tibiotarsus I whorl II total chaetae (TibI II) 7 7(1). Tibiotarsus I whorl III total chaetae (TibI III) 7 7(1). Tibiotarsus I whorl IV (TibI IV) absent. Tibiotarsus I whorl V (TibI V) absent. Tibiotarsus II whorl I total chaetae (TibII I) 7 7(1). Tibiotarsus II whorl II total chaetae (TibII II) 7 7(1). Tibiotarsus II whorl III total chaetae (TibII III) 7 7(1). Tibiotarsus II whorl IV (TibII IV) absent. Tibiotarsus II whorl V (TibII V) absent. Tibiotarsus III whorl I total chaetae (TibIII I) 12 12(1). Tibiotarsus III whorl II total chaetae (TibIII II) 7 7(1). Tibiotarsus III whorl III total chaetae (TibIII III) 7 7(1). Tibiotarsus III whorl IV (TibIII IV) absent.Tibiotarsus III whorl V (TibIII V) absent. Pretarsus I pretarsal chaetae (PTI) 2 2(11). Pretarsus II pretarsal chaetae (PTII) 2 2(11). Pretarsus III pretarsal chaetae (PTIII) 2 2(11). Unguis I inner tooth (UIit) present. Unguis I tunica (UIt) absent. Unguis II inner tooth (UIIit) present. Unguis II tunica (UIIt) absent. Unguis III inner tooth (UIIIit) present. Unguis III tunica (UIIIt) absent. Unguiculus I inner tooth (UNIit) absent. Unguiculus I apical filament (UNIapf) absent. Unguiculus II inner tooth (UNIIit) absent. Unguiculus II apical filament (UNIIapf) absent. Unguiculus III inner tooth (UNIIIit) absent. Unguiculus III apical filament (UNIIIapf) absent. Anterior side total chaetae (AS) 0. Lateral flap total chaetae (LF) 6+6 12(1). Posterior side total chaetae (PS) 5+5 10(1). Tenaculum rami (TR) 4+4 teeth. Tenaculum Corpus (a+p) (TC a+p) 1 1(1). Anterior manubrium total chaetae (MNA) 1+1 2(16). Posterior manubrium total chaetae (NMP) 9+9 18(1). Dental whorl I total chaetae (DNI) 3 3(17). Dental whorl II total chaetae (DNII) 3 3(17). Dental whorl III total chaetae (DNIII) 4 2(1)2(17). Dental whorl IV total chaetae (DNIV) 4 2(1)2(17). Dental whorl V total chaetae (DNV) 1 1(17). Dental anterior total chaetae (DenA) 10+10 20(17). Dental posterior total chaetae (DenP) 5+5 8(1) 2(17). Mucro lamellae total teeth (MU) 5.

***Mucrosomia potapovi* sp. nov. Ferreira & Lima**

**Complete Description:** Pigments (PI) absent. Body color (BC) White. Eye Number (EN) 0+0. Post-antennal organ form (PAO) present entire. Clypeal area (CI) 1+1 2(1). Clypeal area (CII) 1+1 2(1). Clypeal area (CIII) 1 1(1). Clypeal area (CIV) 1+1+1 3(1). Labral formula (a+m+p+pl) 4+5+5+4 7(1)11(2). Anntenal area chaetotaxy (ANT) 24+2+24 50(1). Mandibular area chaetotaxy (MD) 19+2+19 40(1). Maxilar area chaetotaxy dorsal (MX D) 39+2+39 80(1). Maxilar area chaetotaxy dorsal lateral and ventral (MX L+V) 68+68 136(1). Lobe Maxillary distal area (LMX D) 7+7 2(1)8(6)2(7)2(11). Lobe Maxillary basal area (LMX B) 1+1 1(1). Labial area chaetotaxy dorsal and ventral (LB D+V) 17+17 31(1). Postlabial (PostL) 5+5 10(1). Labial triangle total chaetae (LBT) 9+9 18(1). Labial proximal chaetae and papillae chaetotaxy count (LBP) 26+26 8(1)8(3)2(4)2(5)32(6). Thorax I (ThI) 0+0. Thorax II (ThII) 6+6 2(8)8(9)2(10). Thorax III (ThIII) 4+4 2(8)6(9). Thorax III area ventralmedial (VM) 3+3 4(1)2(8). Abdomen I (AbdI) 6+6 6(8)4(9)2(10). Abdomen II (AbdII) 5+5 6(8)4(9). Abdomen III (AbdIII) 5+5 6(8)4(9). Abdomen IV (AbdIV) 7+7 8(8)6(9). Abdomen IV area BPIV L + M total chaetae (BPIV L + M) 18+18 34(1)2(8). Abdomen V and VI sensillar count (AbdV+VI) 5+5 10(9). Antenna I whorl A total chaetae (ANTI A) 15 12(1)2(12). Antenna I whorl B total chaetae (ANTI B) 2 2(11). Antenna II whorl A total chaetae (ANTII A) 8 7(1)1(12). Antenna II whorls -I, 0 and +I total chaetae (ANTI -0+) 12 12(1). Antenna II whorl B total chaetae (ANTII B) 5 3(1)2(11). Antenna III whorl A total chaetae (ANTIII A) 9 4(1)2(12)2(13)1(14). Antenna III whorls -I, 0 and +I total chaetae (ANTIII -0+) 18 18(1). Antenna III whorl B total chaetae (ANTIII B) 2 1(1)1(11). Antenna IV A total chaetae (ANTIV A) 82 52(1)14(11)8(9)7(12)1(15). Antenna IV B total chaetae (ANTIV B) 8 8(1). Subcoxa I area I total chaetae (SCXI I) 0. Subcoxa I area II total chaetae (SCXI II) 1 1(1). Subcoxa II area I total chaetae (SCXII I) 1 1(1). Subcoxa II area II total chaetae (SCXII II) 6 6(1). Subcoxa III area I total chaetae (SCXIII I) 5 5(1). Subcoxa III area II total chaetae (SCXIII II) 7 7(1). Coxa I total chaetae (CXI) 4 4(1). Coxa II total chaetae (CXII) 12 12(1). Coxa III total chaetae (CXIII) 11 11(1). Trochanter I total chaetae (TRI) 9 9(1). Trochanter II total chaetae (TRII) 9 9(1). Trochanter III total chaetae (TRIII) 8 8(1). Femur I total chaetae (FEI) 17 17(1). Femur II total chaetae (FEII) 18 18(1). Femur III total chaetae (FEIII) 21 21(1). Tibiotarsus I whorl I total chaetae (TibI I) 7 7(1). Tibiotarsus I whorl II total chaetae (TibI II) 7 7(1). Tibiotarsus I whorl III total chaetae (TibI III) 7 7(1). Tibiotarsus I whorl IV (TibI IV) absent. Tibiotarsus I whorl V (TibI V) absent. Tibiotarsus II whorl I total chaetae (TibII I) 7 7(1). Tibiotarsus II whorl II total chaetae (TibII II) 7 7(1). Tibiotarsus II whorl III total chaetae (TibII III) 7 7(1). Tibiotarsus II whorl IV (TibII IV) absent. Tibiotarsus II whorl V (TibII V) absent. Tibiotarsus III whorl I total chaetae (TibIII I) 12 12(1). Tibiotarsus III whorl II total chaetae (TibIII II) 7 7(1). Tibiotarsus III whorl III total chaetae (TibIII III) 7 7(1). Tibiotarsus III whorl IV (TibIII IV) absent. Tibiotarsus III whorl V (TibIII V) absent. Pretarsus I pretarsal chaetae (PTI) 2 2(11). Pretarsus II pretarsal chaetae (PTII) 2 2(11). Pretarsus III pretarsal chaetae (PTIII) 2 2(11). Unguis I inner tooth (UIit) present. Unguis I tunica (UIt) absent. Unguis II inner tooth (UIIit) present. Unguis II tunica (UIIt) absent. Unguis III inner tooth (UIIIit) present. Unguis III tunica (UIIIt) absent. Unguiculus I inner tooth (UNIit) absent. Unguiculus I apical filament (UNIapf) absent. Unguiculus II inner tooth (UNIIit) absent. Unguiculus II apical filament (UNIIapf) absent. Unguiculus III inner tooth (UNIIIit) absent. Unguiculus III apical filament (UNIIIapf) absent. Anterior side total chaetae (AS) 0. Lateral flap total chaetae (LF) 5+5 10(1). Posterior side total chaetae (PS) 4+4 8(1). Tenaculum rami (TR) 4+4 teeth. Tenaculum Corpus (a+p) (TC a+p) 1 1(1). Anterior manubrium total chaetae (MNA) 1+1 2(16). Posterior manubrium total chaetae (NMP) 9+9 18(1). Dental whorl I total chaetae (DNI) 3 3(17). Dental whorl II total chaetae (DNII) 3 3(17). Dental whorl III total chaetae (DNIII) 4 2(1)2(17). Dental whorl IV total chaetae (DNIV) 4 2(1)2(17). Dental whorl V total chaetae (DNV) 1 1(17). Dental anterior total chaetae (DenA) 10+10 20(17). Dental posterior total chaetae (DenP) 5+5 8(1) 2(17). Mucro lamellae total teeth (MU) 5.

**Table S1 - Comparative Table of *Mucrosomia* Species and Their Respective Character States**

|  | *M. janssensi sp.nov* | *M.potapowi sp.nov* | *M. alticola* | *M. bipartita* | *M. caeca* | *M. garretti* | *M. novaezealandiae* |
| --- | --- | --- | --- | --- | --- | --- | --- |
| Pigments (PI) | 1 | 1 | 1 | 1 | 1 | 1 | 1 |
| Body color (BC) | 1 | 1 | 1 | 1 | 1 | 1 | 1 |
| Eye Number (EN) | 1 | 1 | 1 | 1 | 1 | 1 | 1 |
| Post-antennal organ form (PAO) | 2 | 2 | 3 | 2 | 3 | 3 | 3 |
| Clypeal area (CI) | 1 | 1 | U | U | U | U | U |
| Clypeal area (CII) | 1 | 1 | U | U | U | U | U |
| Clypeal area (CIII) | 1 | 1 | U | U | U | U | U |
| Clypeal area (CIV) | 1 | 1 | U | U | U | U | U |
| Labral formula (a+m+p+pl) | 1 | 2 | 2 | 1 | 1 | 1 | U |
| Anntenal area chaetotaxy (ANT) | 1 | 2 | U | U | U | U | U |
| Mandibular area chaetotaxy (MD) | 1 | 2 | U | U | U | U | U |
| Maxilar area chaetotaxy dorsal (MX D) | 1 | 2 | U | U | U | U | U |
| Maxilar area chaetotaxy dorsal lateral and ventral (MX L+V) | 1 | 2 | U | U | U | U | U |
| Lobe Maxillary distal area (LMX D) | 1 | 2 | 3 | 1 | U | U | U |
| Lobe Maxillary basal area (LMX B) | 1 | 1 | 1 | 1 | U | U | U |
| Labial area chaetotaxy dorsal and ventral (LB D+V) | 1 | 2 | U | U | U | U | U |
| Postlabial (PostL) | 1 | 1 | 1 | U | U | 1 | U |
| Labial triangle total chaetae (LBT) | 1 | 1 | U | U | U | 2 | U |
| Labial proximal chaetae and papillae chaetotaxy count (LBP) | 1 | 1 | 1 | U | U | 1 | U |
| Thorax I (ThI) | 1 | 1 | 1 | 1 | U | 1 | U |
| Thorax II (ThII) | 1 | 1 | 1 | 1 | U | 1 | U |
| Thorax III (ThIII) | 1 | 1 | 1 | 1 | U | 1 | U |
| Thorax III area ventralmedial (VM) | 1 | 1 | 1 | 2 | 2 | 2 | 2 |
| Abdomen I (AbdI) | 1 | 1 | 2 | 1 | U | 1 | U |
| Abdomen II (AbdII) | 1 | 1 | 1 | 1 | U | 1 | U |
| Abdomen III (AbdIII) | 1 | 1 | 1 | 1 | U | 1 | U |
| Abdomen IV (AbdIV) | 1 | 1 | 1 | 1 | U | 1 | U |
| Abdomen IV area BPIV L + M total chaetae (BPIV L + M) | 1 | 2 | U | 3 | U | U | U |
| Abdomen V and VI sensillar count (AbdV+VI) | 2 | 2 | 2 | 2 | U | 2 | U |
| Antenna I whorl A total chaetae (ANTI A) | 1 | 1 | 2 | 3 | U | U | U |
| Antenna I whorl B total chaetae (ANTI B) | 1 | 1 | 1 | 1 | U | U | U |
| Antenna II whorl A total chaetae (ANTII A) | 1 | 1 | 1 | 1 | U | U | U |
| Antenna II whorls -I, 0 and +I total chaetae (ANTI -0+) | 1 | 1 | 2 | 3 | U | U | U |
| Antenna II whorl B total chaetae (ANTII B) | 1 | 1 | 1 | 2 | U | U | U |
| Antenna III whorl A total chaetae (ANTIII A) | 1 | 1 | 1 | 1 | U | U | U |
| Antenna III whorls -I, 0 and +I total chaetae (ANTIII -0+) | 1 | 2 | 2 | 3 | U | U | U |
| Antenna III whorl B total chaetae (ANTIII B) | 1 | 1 | 2 | U | U | U | U |
| Antenna IV A total chaetae (ANTIV A) | 1 | 2 | U | U | U | U | U |
| Antenna IV B total chaetae (ANTIV B) | 1 | 1 | 1 | U | U | U | U |
| Subcoxa I area I total chaetae (SCXI I) | 1 | 1 | U | U | U | U | U |
| Subcoxa I area II total chaetae (SCXI II) | 1 | 1 | U | U | U | U | U |
| Subcoxa II area I total chaetae (SCXII I) | 1 | 1 | U | U | U | U | U |
| Subcoxa II area II total chaetae (SCXII II) | 1 | 1 | U | U | U | U | U |
| Subcoxa III area I total chaetae (SCXIII I) | 1 | 1 | 1 | U | U | U | U |
| Subcoxa III area II total chaetae (SCXIII II) | 1 | 2 | 2 | U | U | U | U |
| Coxa I total chaetae (CXI) | 1 | 2 | U | U | U | U | U |
| Coxa II total chaetae (CXII) | 1 | 1 | U | U | U | U | U |
| Coxa III total chaetae (CXIII) | 1 | 1 | U | U | U | U | U |
| Trochanter I total chaetae (TRI) | 1 | 1 | U | U | U | U | U |
| Trochanter II total chaetae (TRII) | 1 | 1 | U | U | U | U | U |
| Trochanter III total chaetae (TRIII) | 1 | 2 | 2 | U | U | U | U |
| Femur I total chaetae (FEI) | 1 | 1 | U | U | U | U | U |
| Femur II total chaetae (FEII) | 1 | 1 | U | U | U | U | U |
| Femur III total chaetae (FEIII) | 1 | 1 | 1 | U | U | U | U |
| Tibiotarsus I whorl I total chaetae (TibI I) | 1 | 1 | U | U | U | U | U |
| Tibiotarsus I whorl II total chaetae (TibI II) | 1 | 1 | U | U | U | U | U |
| Tibiotarsus I whorl III total chaetae (TibI III) | 1 | 1 | U | U | U | U | U |
| Tibiotarsus I whorl IV (TibI IV) | 1 | 1 | U | U | U | U | U |
| Tibiotarsus I whorl V (TibI V) | 1 | 1 | U | U | U | U | U |
| Tibiotarsus II whorl I total chaetae (TibII I) | 1 | 1 | U | U | U | U | U |
| Tibiotarsus II whorl II total chaetae (TibII II) | 1 | 1 | U | U | U | U | U |
| Tibiotarsus II whorl III total chaetae (TibII III) | 1 | 1 | U | U | U | U | U |
| Tibiotarsus II whorl IV (TibII IV) | 1 | 1 | U | U | U | U | U |
| Tibiotarsus II whorl V (TibII V) | 1 | 1 | U | U | U | U | U |
| Tibiotarsus III whorl I total chaetae (TibIII I) | 1 | 1 | U | U | U | U | U |
| Tibiotarsus III whorl II total chaetae (TibIII II) | 1 | 1 | U | U | U | U | U |
| Tibiotarsus III whorl III total chaetae (TibIII III) | 1 | 1 | U | U | U | U | U |
| Tibiotarsus III whorl IV (TibIII IV) | 1 | 1 | U | U | U | U | U |
| Tibiotarsus III whorl V (TibIII V) | 1 | 1 | U | U | U | U | U |
| Pretarsus I pretarsal chaetae (PTI) | 1 | 1 | 1 | 1 | U | U | 1 |
| Pretarsus II pretarsal chaetae(PTII) | 1 | 1 | 1 | 1 | U | U | 1 |
| Pretarsus III pretarsal chaetae (PTIII) | 1 | 1 | 1 | 1 | U | U | 1 |
| Unguis I inner tooth (UIit) | 1 | 1 | 1 | 2 | 1 | 1 | 2 |
| Unguis I tunica (UIt) | 1 | 1 | 1 | 1 | U | U | U |
| Unguis II inner tooth (UIIit) | 1 | 1 | 1 | 2 | 1 | 1 | 2 |
| Unguis II tunica (UIIt) | 1 | 1 | 1 | 1 | U | U | U |
| Unguis III inner tooth (UIIIit) | 1 | 1 | 1 | 2 | 1 | 1 | 2 |
| Unguis III tunica (UIIIt) | 1 | 1 | 1 | 1 | U | U | U |
| Unguiculus I inner tooth (UNIit) | 1 | 1 | 1 | U | U | U | U |
| Unguiculus I apical filament (UNIapf) | 1 | 1 | 1 | 1 | U | 1 | U |
| Unguiculus II inner tooth (UNIIit) | 1 | 1 | 1 | U | U | U | U |
| Unguiculus II apical filament (UNIIapf) | 1 | 1 | 1 | 1 | U | 1 | U |
| Unguiculus III inner tooth (UNIIIit) | 1 | 1 | 1 | U | U | U | U |
| Unguiculus III apical filament (UNIIIapf) | 1 | 1 | 1 | 1 | U | 1 | U |
| Anterior side total chaetae (AS) | 1 | 1 | 1 | 1 | U | 1 | U |
| Lateral flap total chaetae (LF) | 1 | 2 | 3 | 4 | 2 | 5 | U |
| Posterior side total chaetae (PS) | 1 | 2 | 2 | 3 | U | 3 | U |
| Tenaculum rami (TR) | 1 | 1 | 1 | 1 | U | 1 | U |
| Tenaculum Corpus (a+p) (TC a+p) | 1 | 1 | 1 | 1 | U | 2 | U |
| Anterior manubrium total chaetae (MNA) | 1 | 1 | 1 | 1 | 1 | 1 | U |
| Posterior manubrium total chaetae (NMP) | 1 | 1 | 2 | U | U | U | U |
| Dental whorl I total chaetae (DNI) | 1 | 1 | 1 | 1 | 1 | 1 | U |
| Dental whorl II total chaetae (DNII) | 1 | 1 | 1 | 1 | 1 | 1 | U |
| Dental whorl III total chaetae (DNIII) | 1 | 1 | 1 | 1 | 1 | 1 | U |
| Dental whorl IV total chaetae (DNIV) | 1 | 1 | 1 | 1 | 1 | 1 | U |
| Dental whorl V total chaetae (DNV) | 1 | 1 | 1 | 1 | 1 | 1 | U |
| Dental anterior total chaetae (DenA) | 1 | 1 | 1 | 1 | 1 | 1 | U |
| Dental posterior total chaetae (DenP) | 1 | 1 | 1 | 1 | 1 | 1 | U |
| Mucro lamellae total teeth (MU) | 1 | 1 | 1 | 1 | 1 | 1 | U |

***Mucrosomia alticola* Mendonça & Queiroz, 2013** (Figs. S1–S5)

**Complete Description:** Pigments (PI) absent. Body color (BC) White. Eye Number (EN) 0+0. Post-antennal organ form (PAO) present with constriction in the middle. Labral formula (a+m+p+pl) 4+5+5+4 7(1)11(2). Lobe Maxillary distal area (LMX D) 7+7 2(1)10(6)2(7). Lobe Maxillary basal area (LMX B) 1+1 1(1). Postlabial (PostL) 5+5 10(1). Labial proximal chaetae and papillae chaetotaxy count (LBP) 26+26 8(1)8(3)2(4)2(5)32(6). Thorax I (ThI) 0+0. Thorax II (ThII) 6+6 2(8)8(9)2(10). Thorax III (ThIII) 4+4 2(8)6(9). Thorax III area ventralmedial (VM) 3+3 4(1)2(8). Abdomen I (AbdI) 4+4 2(8)4(9)2(10). Abdomen II (AbdII) 5+5 6(8)4(9). Abdomen III (AbdIII) 5+5 6(8)4(9). Abdomen IV (AbdIV) 7+7 8(8)6(9). Abdomen V and VI sensillar count (AbdV+VI) 5+5 10(9). Antenna I whorl A total chaetae (ANTI A) 12 10(1)2(12). Antenna I whorl B total chaetae (ANTI B) 2 2(11). Antenna II whorl A total chaetae (ANTII A) 8 7(1)1(12). Antenna II whorls -I, 0 and +I total chaetae (ANTI -0+) 15 15(1). Antenna II whorl B total chaetae (ANTII B) 5 3(1)2(11). Antenna III whorl A total chaetae (ANTIII A) 9 4(1)2(12)2(13)1(14). Antenna III whorls -I, 0 and +I total chaetae (ANTIII -0+) 18 18(1). Antenna III whorl B total chaetae (ANTIII B) 1 1(11). Antenna IV B total chaetae (ANTIV B) 8 8(1). Subcoxa III area I total chaetae (SCXIII I) 5 5(1). Subcoxa III area II total chaetae (SCXIII II) 7 7(1). Trochanter III total chaetae (TRIII) 8 8(1). Femur III total chaetae (FEIII) 21 21(1). Pretarsus I pretarsal chaetae (PTI) 2 2(11). Pretarsus II pretarsal chaetae(PTII) 2 2(11). Pretarsus III pretarsal chaetae (PTIII) 2 2(11). Unguis I inner tooth (UIit) present. Unguis I tunica (UIt) absent. Unguis II inner tooth (UIIit) present. Unguis II tunica (UIIt) absent. Unguis III inner tooth (UIIIit) present. Unguis III tunica (UIIIt) absent. Unguiculus I inner tooth (UNIit) absent. Unguiculus I apical filament (UNIapf) absent. Unguiculus II inner tooth (UNIIit) absent. Unguiculus II apical filament (UNIIapf) absent. Unguiculus III inner tooth (UNIIIit) absent. Unguiculus III apical filament (UNIIIapf) absent. Anterior side total chaetae (AS) 0. Lateral flap total chaetae (LF) 4-7+4-7 8-14(1). Posterior side total chaetae (PS) 4+4 8(1). Tenaculum rami (TR) 4+4 teeth. Tenaculum Corpus (a+p) (TC a+p) 1 1(1). Anterior manubrium total chaetae (MNA) 1+1 2(16). Posterior manubrium total chaetae (NMP) 10+10 20(1). Dental whorl I total chaetae (DNI) 3 3(17). Dental whorl II total chaetae (DNII) 3 3(17). Dental whorl III total chaetae (DNIII) 4 2(1)2(17). Dental whorl IV total chaetae (DNIV) 4 2(1)2(17). Dental whorl V total chaetae (DNV) 1 1(17). Dental anterior total chaetae (DenA) 10+10 20(17). Dental posterior total chaetae (DenP) 5+5 8(1) 2(17). Mucro lamellae total teeth (MU) 5.

**Coded Description:**


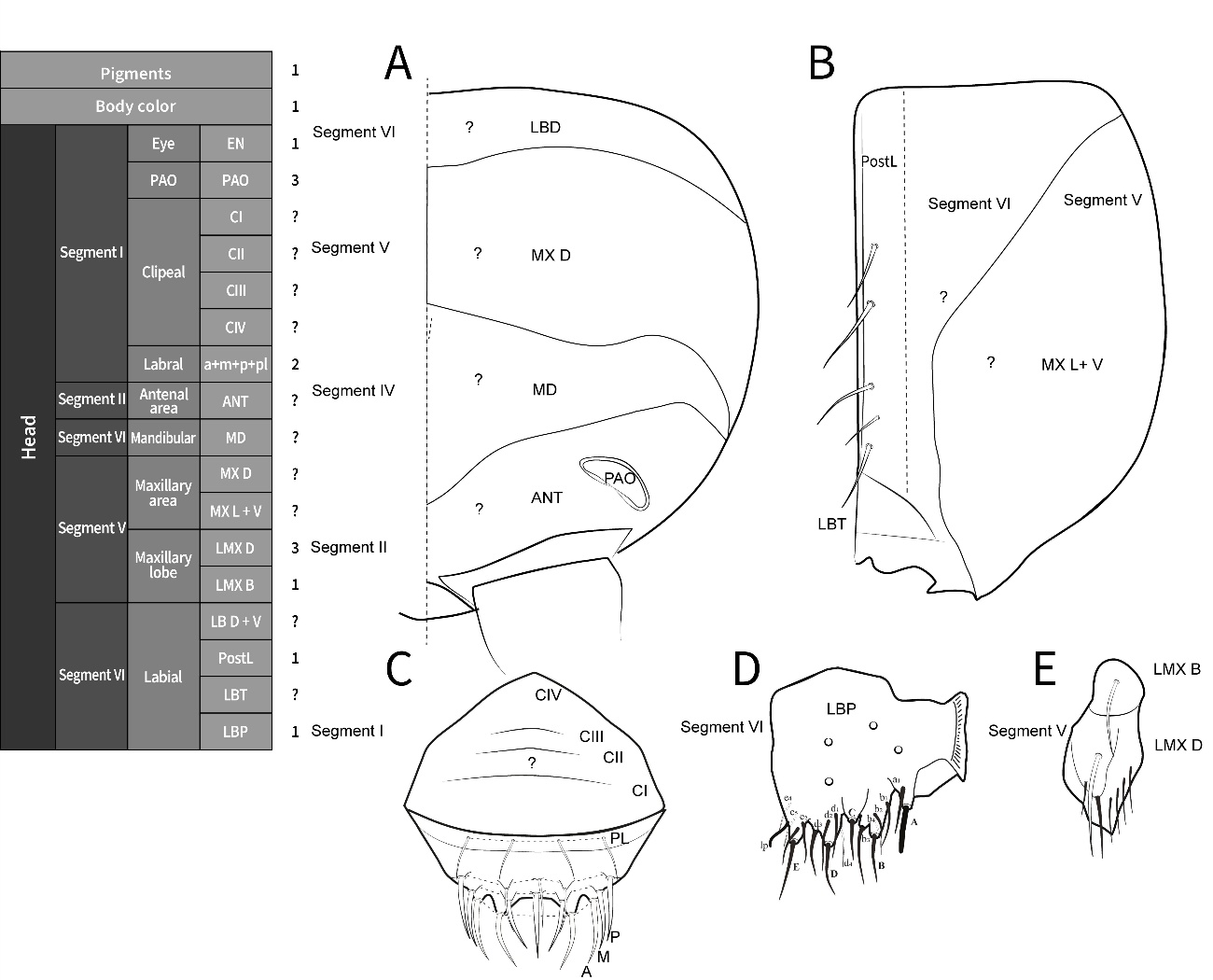


**Fig. S1** *Mucrosomia alticola.* Cephalic chaetotaxy and descriptive table. (**A**) Dorsal cephalic schematic chaetotaxy; (**B**) Labial triangle, medial and distal chaetotaxy; (**C**) Clypeal and labral chaetotaxy; (**D**) Labial proximal chaetotaxy; (**E**) Maxillary lobe. “?” indicates unavailable data


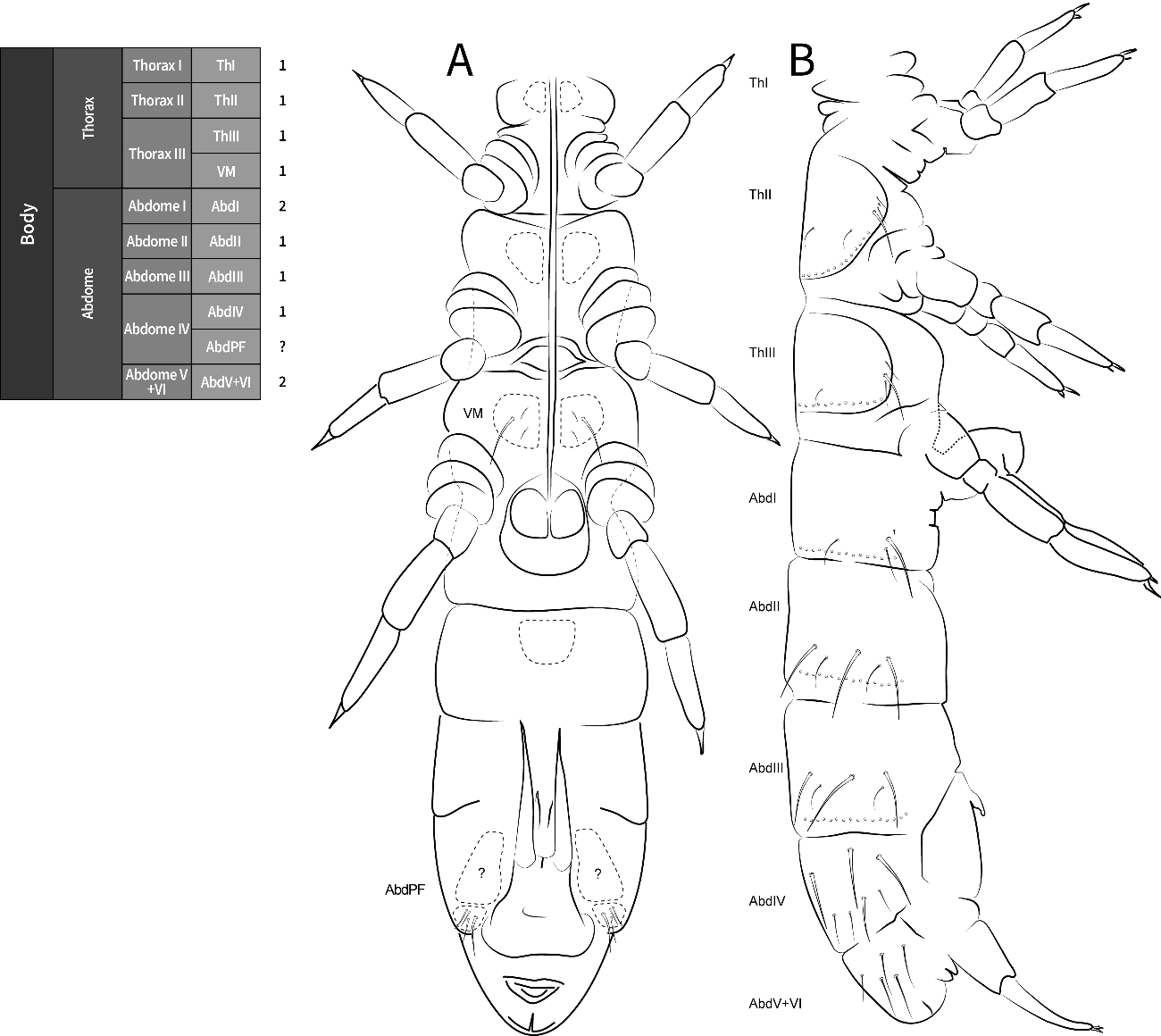


**Fig. S2** *Mucrosomia alticola.* Body chaetotaxy and descriptive table. (**A**) Body in ventral view; (**B**) Body in dorsal view. “?” indicates unavailable data


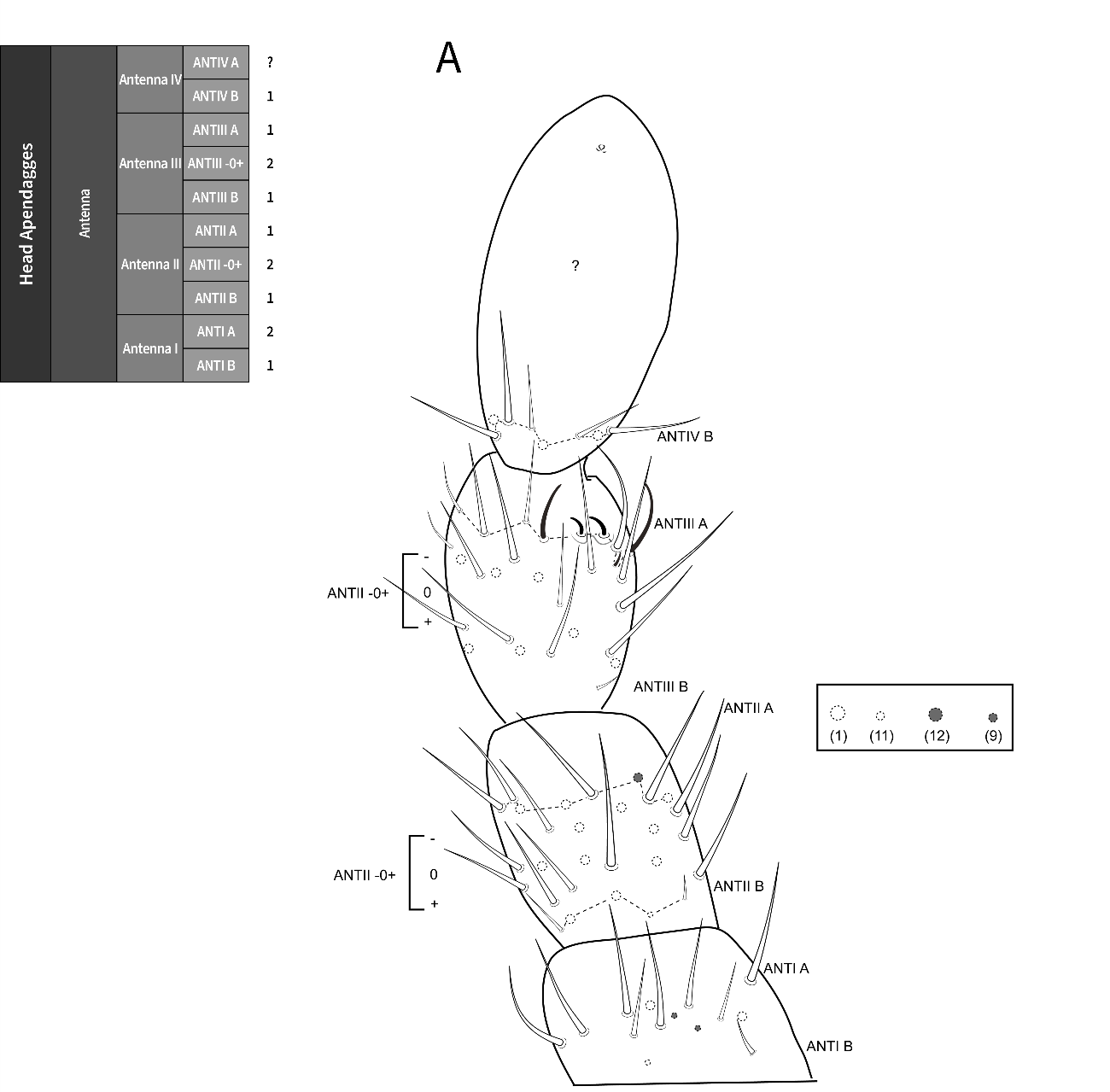


**Fig. S3** *Mucrosomia alticola.* Antennal chaetotaxy and descriptive table. (**A**) Entire antennal chaetotaxy. Chaetal types (1), (9), (11) and (12) follow those illustrated in the chaetal bank (see Fig.1)


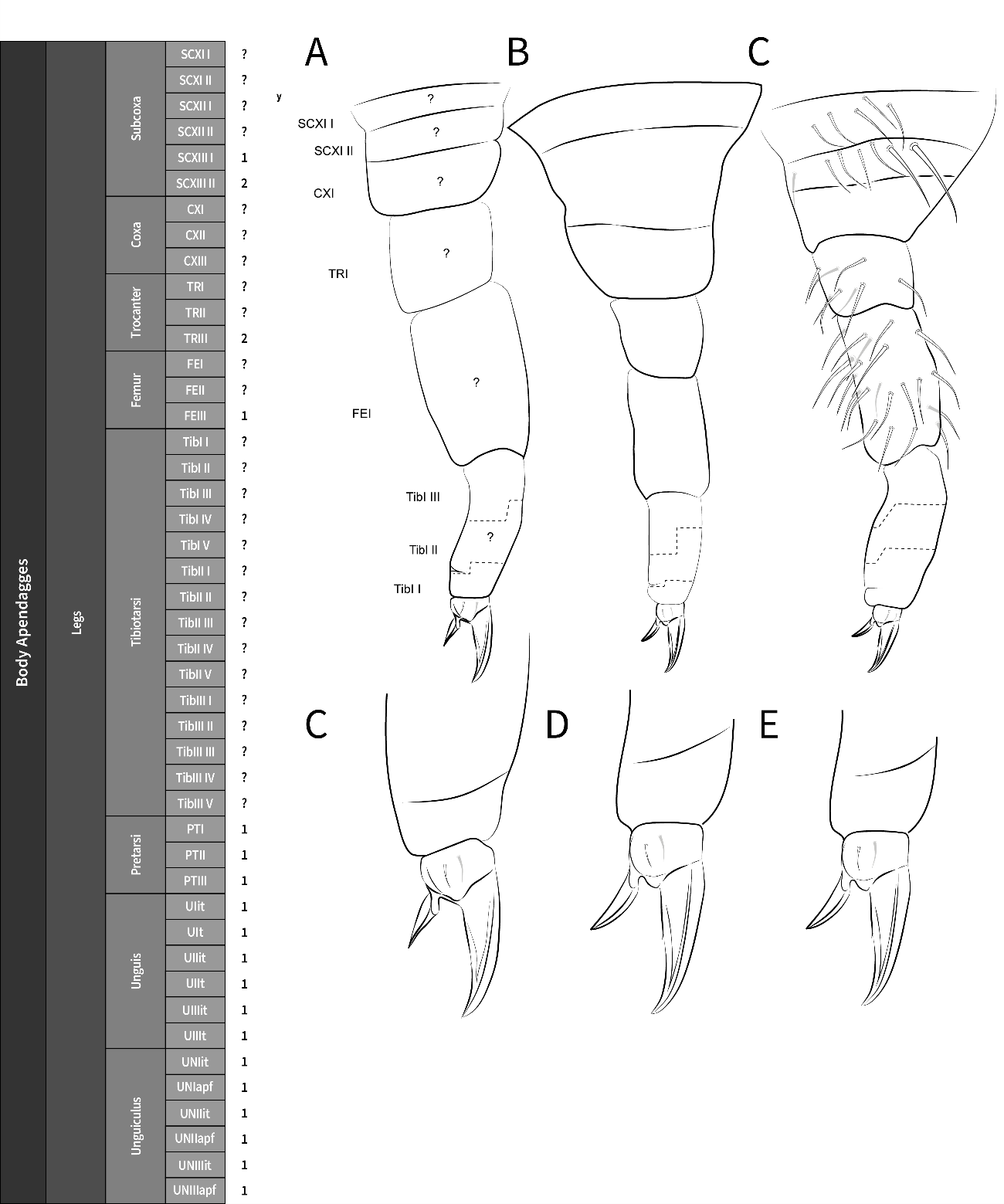


**Fig. S4** *Mucrosomia alticola.* Leg chaetotaxy and descriptive table. (**A**) Leg I; (**B**) Leg II; (**C**) Leg III; (**D**, **E**, **F**) Details of the apices of legs I–III. “?” indicates unavailable data. “?” indicates unavailable data


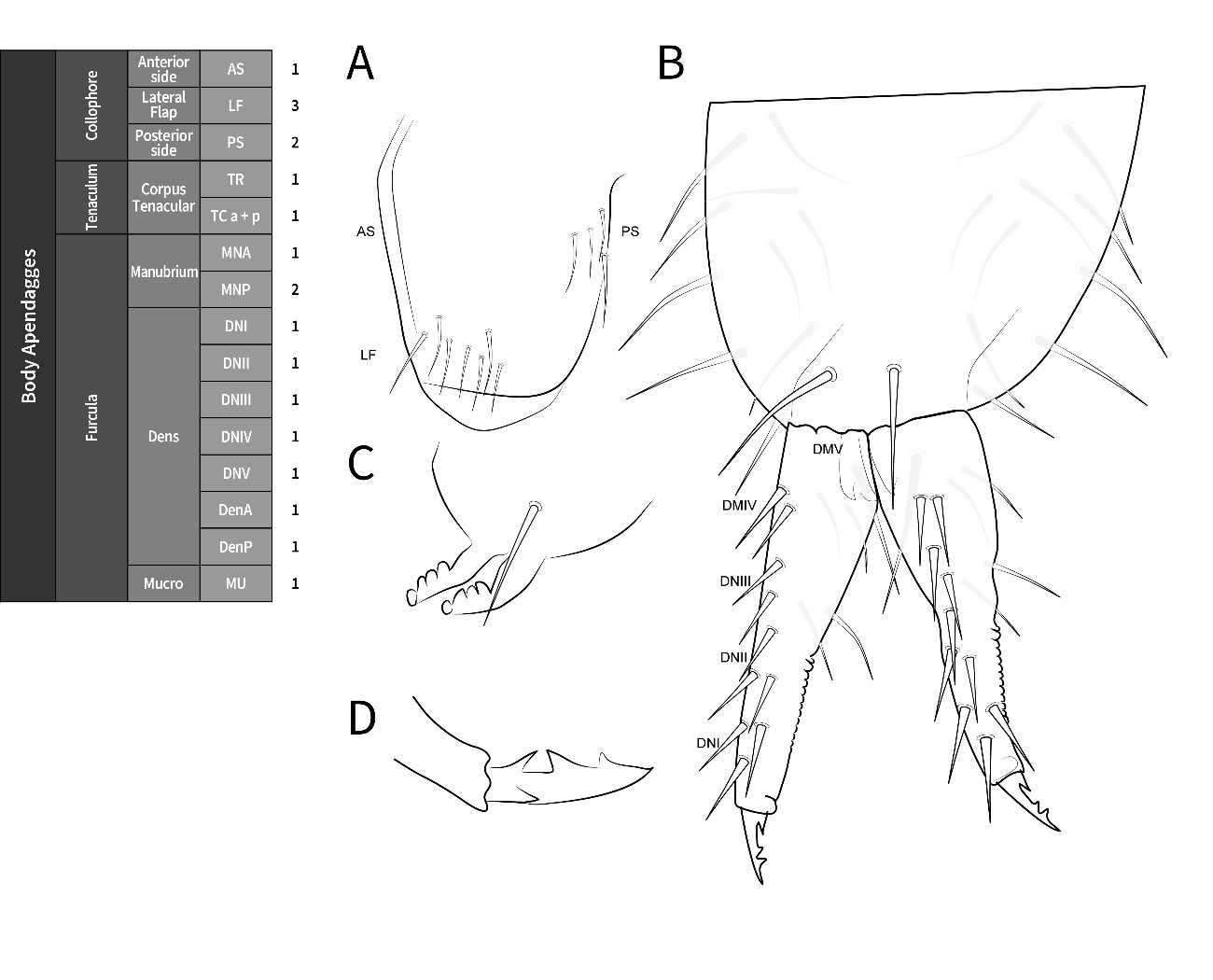


**Fig. S5** *Mucrosomia alticola.* Abdominal appendages chaetotaxy. (**A**) Collophore: anterior side (AS), lateral flap (LF), and posterior side (PS); (**B**) Furcula; (**C**) Tenaculum in anterior view; (**D**) Mucro. The lighter chaetae are located on the posterior side. “?” indicates unavailable data

***Mucrosomia bipartita* (Rusek, 1996)** (Figs. S6–S10)

**Complete Description.** Pigments (PI) absent. Body color (BC) White. Eye Number (EN) 0+0. Post-antennal organ form (PAO) present entire. Labral formula (a+m+p+pl) 4+5+5+3 8(1)9(2). Lobe Maxillary distal area (LMX D) 6+6 2(1)8(6)2(7). Lobe Maxillary basal area (LMX B) 1+1 1(1). Thorax I (ThI) 0+0. Thorax II (ThII) 6+6 2(8)8(9)2(10). Thorax III (ThIII) 4+4 2(8)6(9). Thorax III area ventralmedial (VM) 0+0. Abdomen I (AbdI) 6+6 6(8)4(9)2(10). Abdomen II (AbdII) 5+5 6(8)4(9). Abdomen III (AbdIII) 5+5 6(8)4(9). Abdomen IV (AbdIV) 7+7 8(8)6(9). Abdomen IV area BPIV L + M total chaetae (BPIV L + M) 17+17 32(1)2(8). Abdomen V and VI sensillar count (AbdV+VI) 5+5 10(9). Antenna I whorl A total chaetae (ANTI A) 13 10(1)3(12). Antenna I whorl B total chaetae (ANTI B) 2 2(11). Antenna II whorl A total chaetae (ANTII A) 8 7(1)1(12). Antenna II whorls -I, 0 and +I total chaetae (ANTI -0+) 10 10(1). Antenna II whorl B total chaetae (ANTII B) 5 4(1)1(11). Antenna III whorl A total chaetae (ANTIII A) 9 4(1)2(12)2(13)1(14). Antenna III whorls -I, 0 and +I total chaetae (ANTIII -0+) 16 16(1). Pretarsus I pretarsal chaetae (PTI) 2 2(11). Pretarsus II pretarsal chaetae(PTII) 2 2(11). Pretarsus III pretarsal chaetae (PTIII) 2 2(11). Unguis I inner tooth (UIit) absent. Unguis I tunica (UIt) absent. Unguis II inner tooth (UIIit) absent. Unguis II tunica (UIIt) absent. Unguis III inner tooth (UIIIit) absent. UnguisIII tunica (UIIIt) absent. Unguiculus I apical filament (UNIapf) absent. Unguiculus II apical filament (UNIIapf) absent. Unguiculus III apical filament (UNIIIapf) absent. Anterior side total chaetae (AS) 0. Lateral flap total chaetae (LF) 7+7 14(1). Posterior side total chaetae (PS) 3+3 6(1). Tenaculum rami (TR) 4+4 teeth. Tenaculum Corpus (a+p) (TC a+p) 1 1(1). Anterior manubrium total chaetae (MNA) 1+1 2(16). Dental whorl I total chaetae (DNI) 3 3(17). Dental whorl II total chaetae (DNII) 3 3(17). Dental whorl III total chaetae (DNIII) 4 2(1)2(17). Dental whorl IV total chaetae (DNIV) 4 2(1)2(17). Dental whorl V total chaetae (DNV) 1 1(17). Dental anterior total chaetae (DenA) 10+10 20(17). Dental posterior total chaetae (DenP) 5+5 8(1) 2(17). Mucro lamellae total teeth (MU) 5.

**Coded description:**


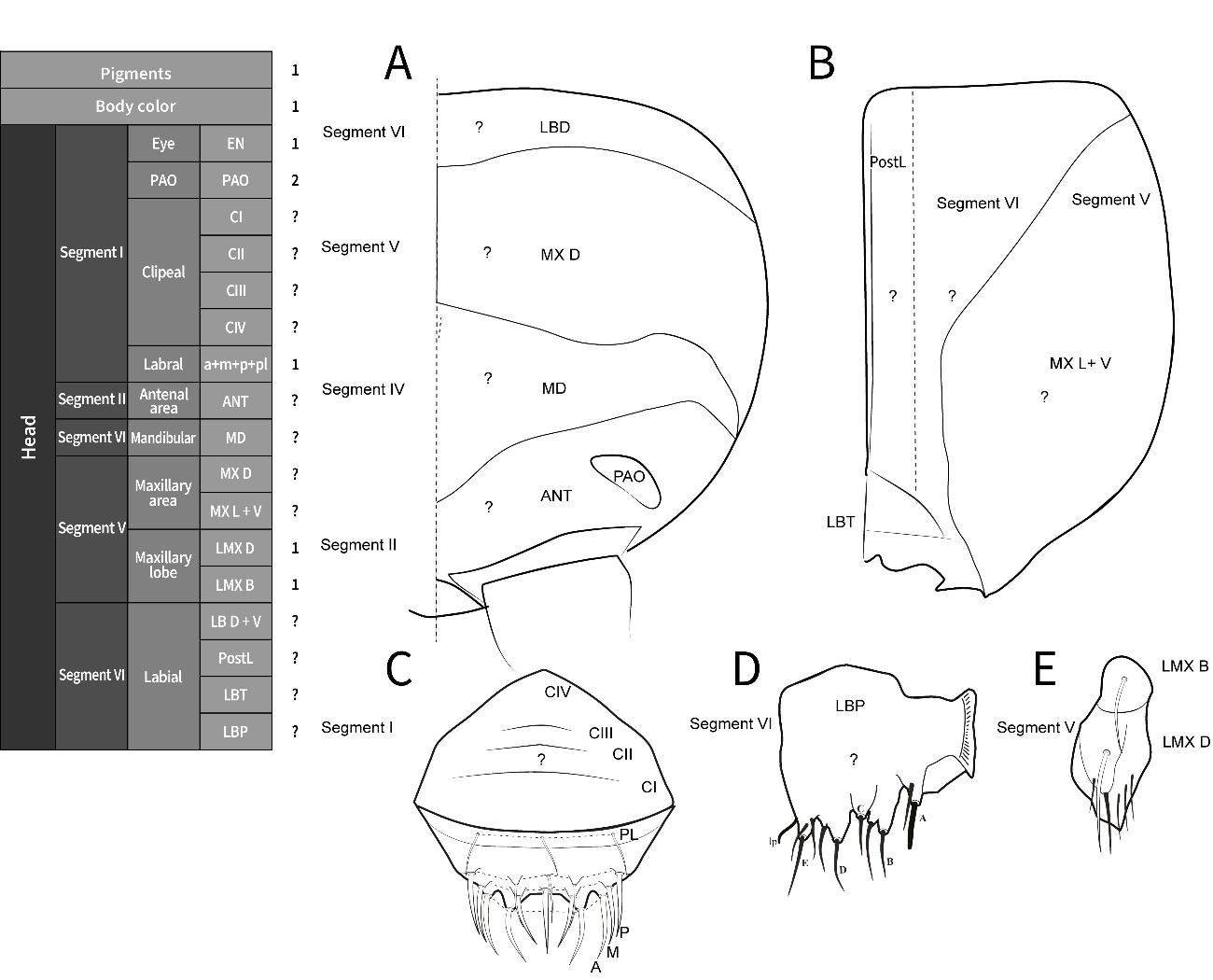


**Fig. S6** *Mucrosomia bipartita.* Cephalic chaetotaxy and descriptive table. (**A**) Dorsal cephalic schematic chaetotaxy; (**B**) Labial triangle, medial and distal chaetotaxy; (**C**) Clypeal and labral chaetotaxy; (**D**) Labial proximal chaetotaxy; (**E**) Maxillary lobe. “?” indicates unavailable data


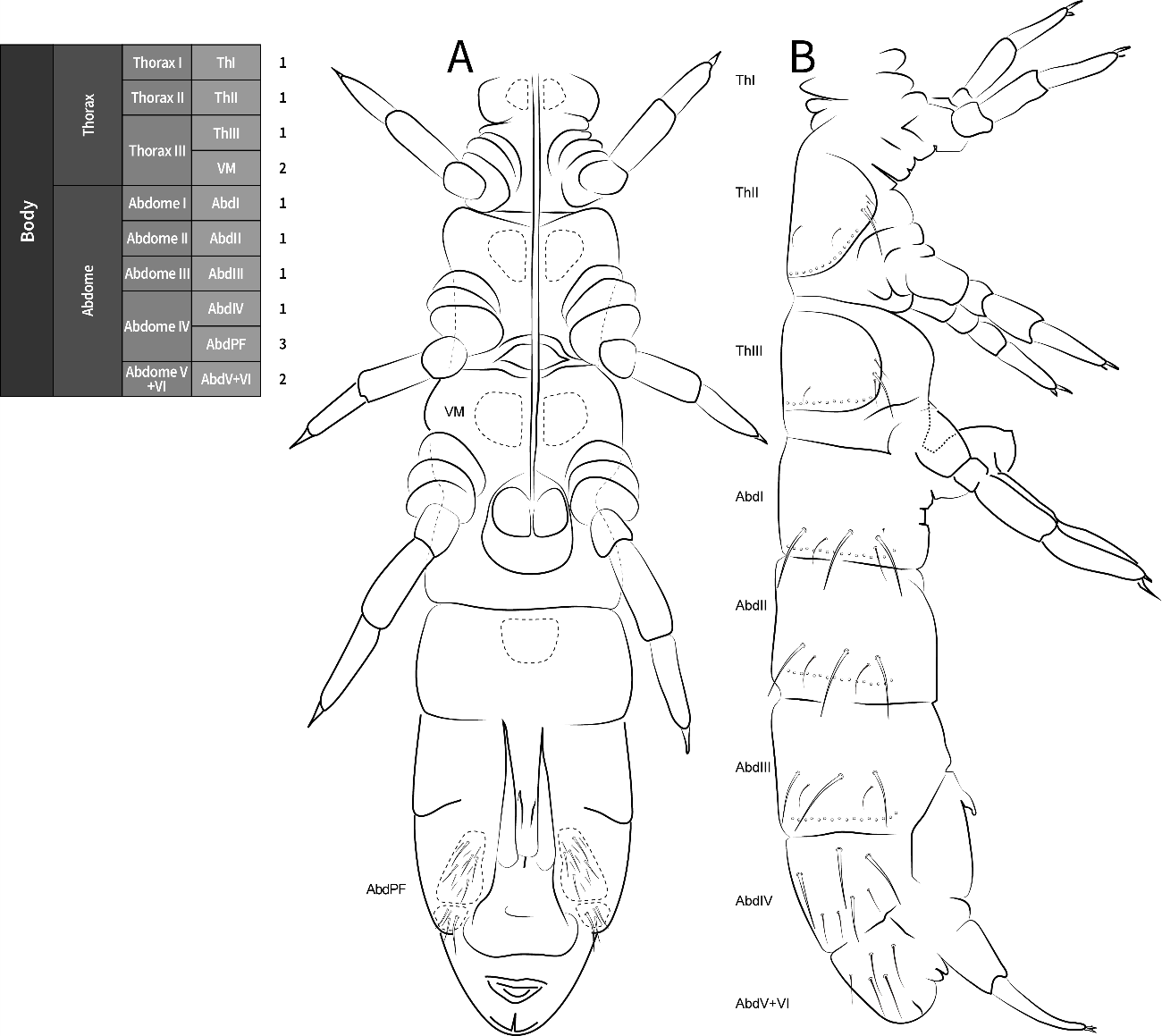


**Fig. S7** *Mucrosomia bipartita.* Body chaetotaxy and descriptive table. (**A**) Body in ventral view; (**B**) Body in dorsal view. “?” indicates unavailable data


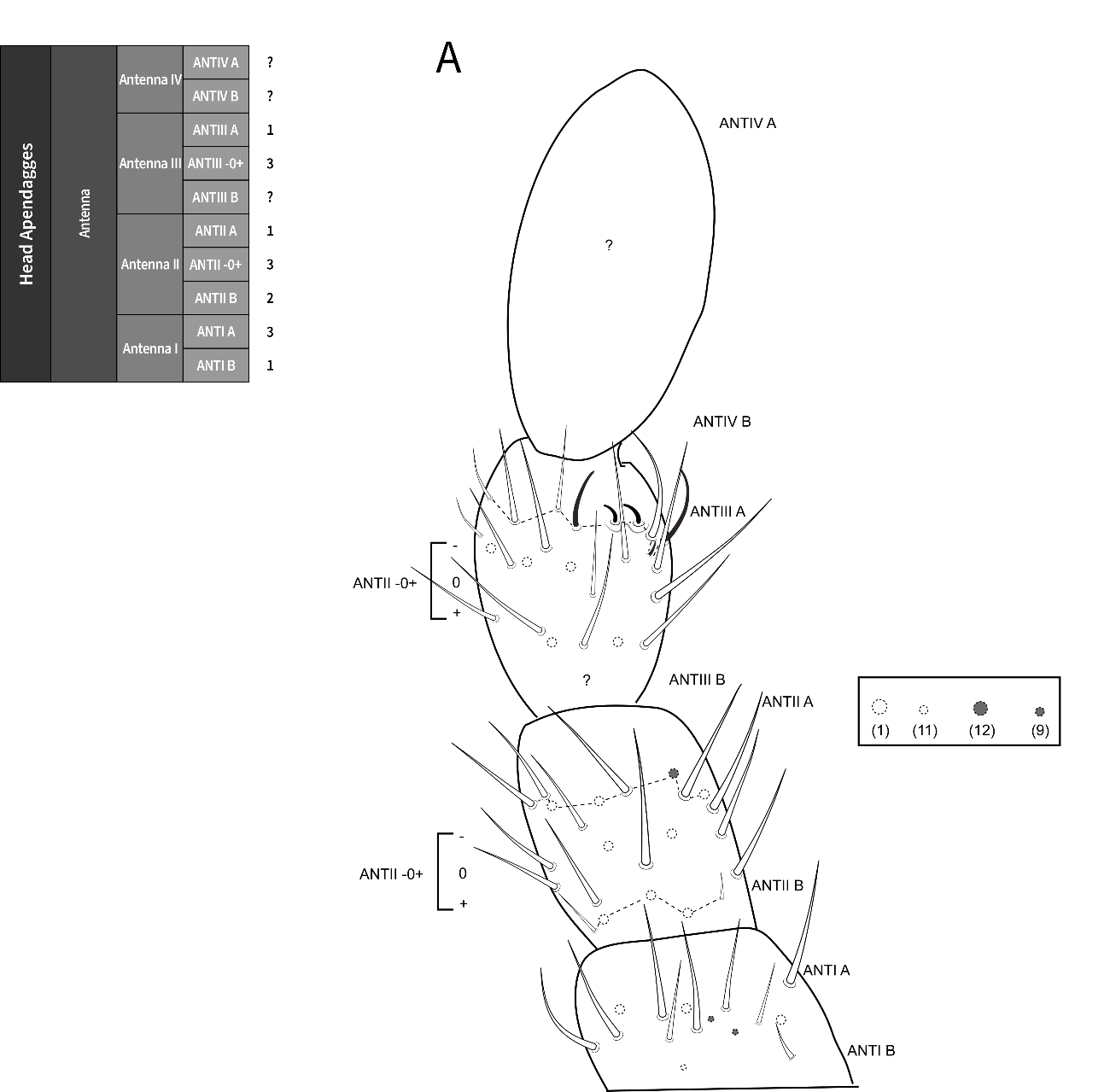


**Fig. S8** *Mucrosomia bipartita.* Antennal chaetotaxy and descriptive table. (**A**) Entire antennal chaetotaxy. Chaetal types (1), (9), (11) and (12) follow those illustrated in the chaetal bank (see Fig. 1)


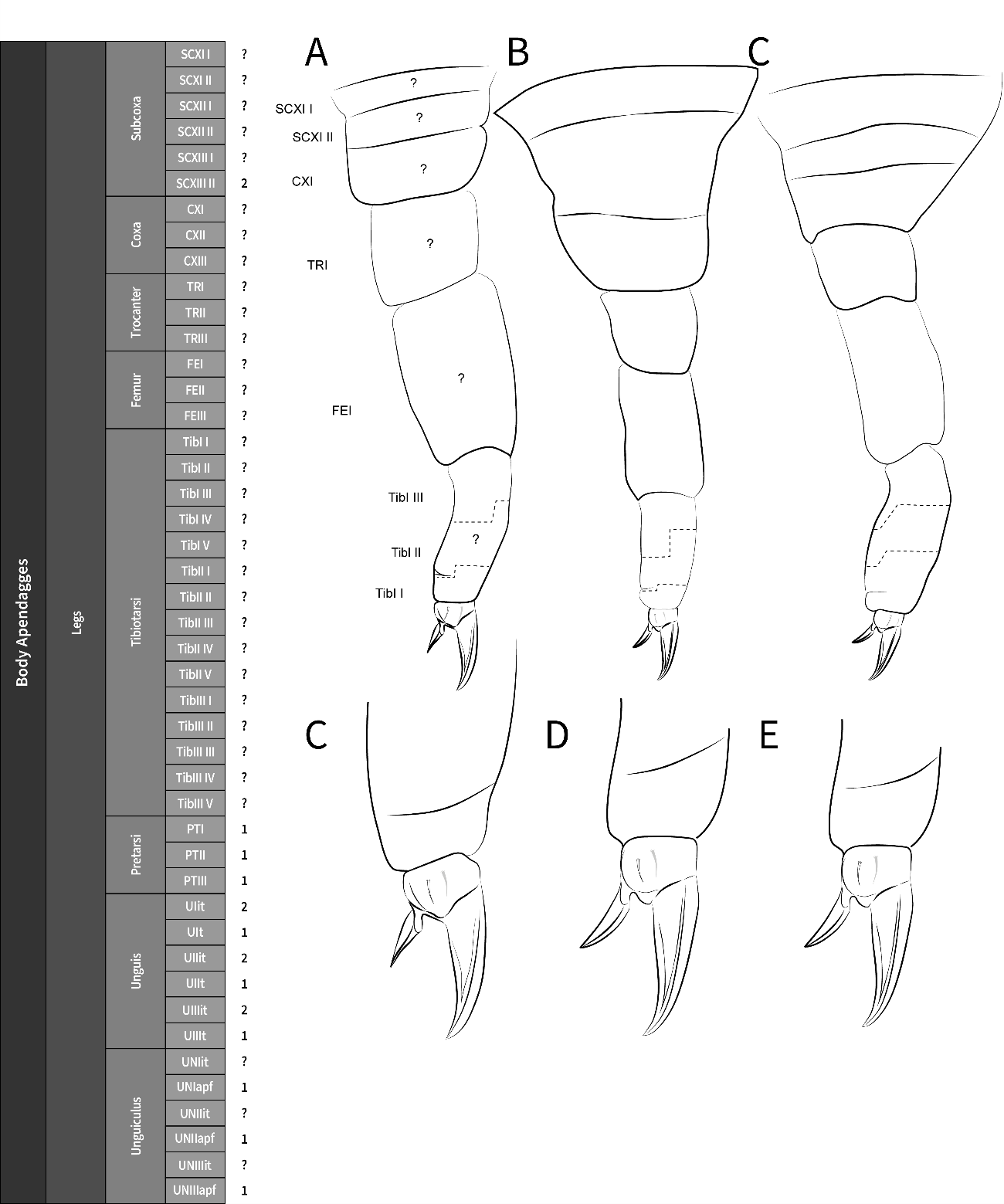


**Fig. S9** *Mucrosomia bipartita.* Leg chaetotaxy and descriptive table. (**A**) Leg I; (**B**) Leg II; (**C**) Leg III; (**D**, **E**, **F**) Details of the apices of legs I–III. “?” indicates unavailable data


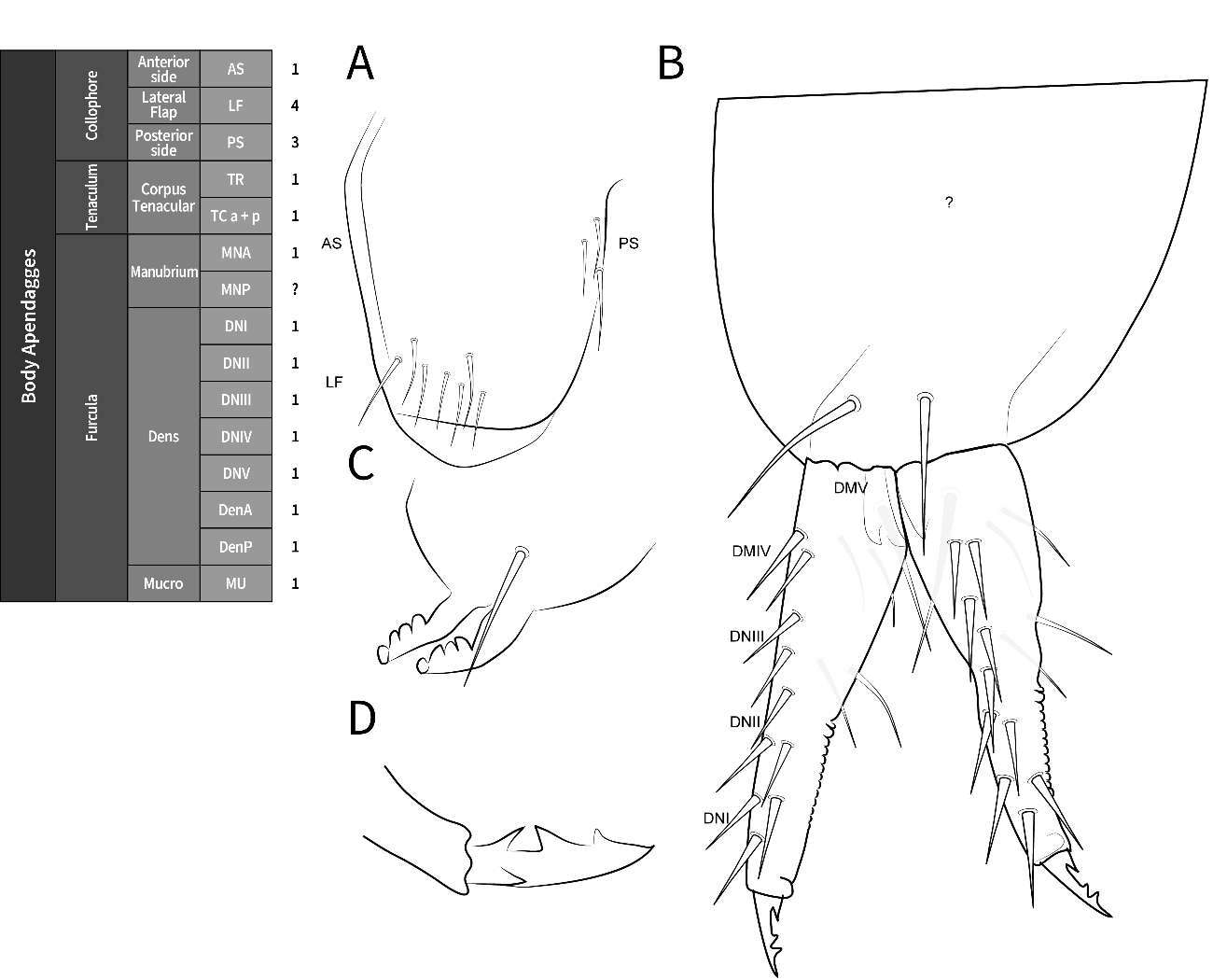


**Fig. S10** *Mucrosomia bipartita.* Abdominal appendages chaetotaxy. (**A**) Collophore: anterior side (AS), lateral flap (LF), and posterior side (PS); (**B**) Furcula; (**C**) Tenaculum in anterior view; (**D**) Mucro. The lighter chaetae are located on the posterior side. “?” indicates unavailable data

***Mucrosomia caeca* (Wahlgren, 1906)** (Figs. S11–S15)

**Complete Description.** Pigments (PI) absent. Body color (BC) White. Eye Number (EN) 0+0. Post-antennal organ form (PAO) present with constriction in the middle. Labral formula (a+m+p+pl) 4+5+5+3 8(1)9(2). Thorax III area ventralmedial (VM) 0+0. Unguis I inner tooth (UIit) present. Unguis II inner tooth (UIIit) present. Unguis III inner tooth (UIIIit) present. Lateral flap total chaetae (LF) 5+5 10(1). Anterior manubrium total chaetae (MNA) 1+1 2(16). Dental whorl I total chaetae (DNI) 3 3(17). Dental whorl II total chaetae (DNII) 3 3(17). Dental whorl III total chaetae (DNIII) 4 2(1)2(17). Dental whorl IV total chaetae (DNIV) 4 2(1)2(17). Dental whorl V total chaetae (DNV) 1 1(17). Dental anterior total chaetae (DenA) 10+10 20(17). Dental posterior total chaetae (DenP) 5+5 8(1) 2(17). Mucro lamellae total teeth (MU) 5.

**Coded description:**


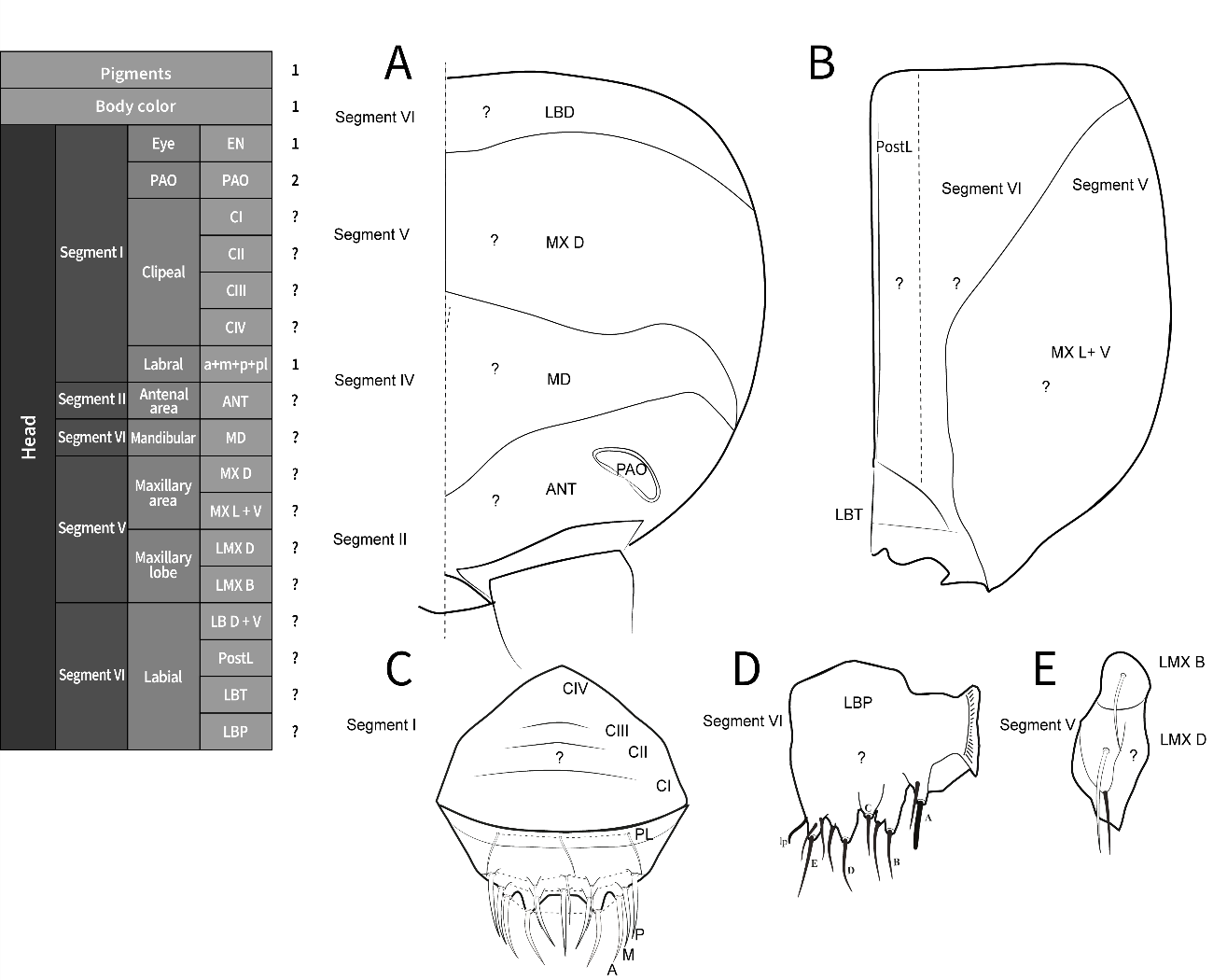


**Fig. S11** *Mucrosomia caeca.* Cephalic chaetotaxy and descriptive table. (**A**) Dorsal cephalic schematic chaetotaxy; (**B**) Labial triangle, medial and distal chaetotaxy; (**C**) Clypeal and labral chaetotaxy; (**D**) Labial proximal chaetotaxy; (**E**) Maxillary lobe. “?” indicates unavailable data


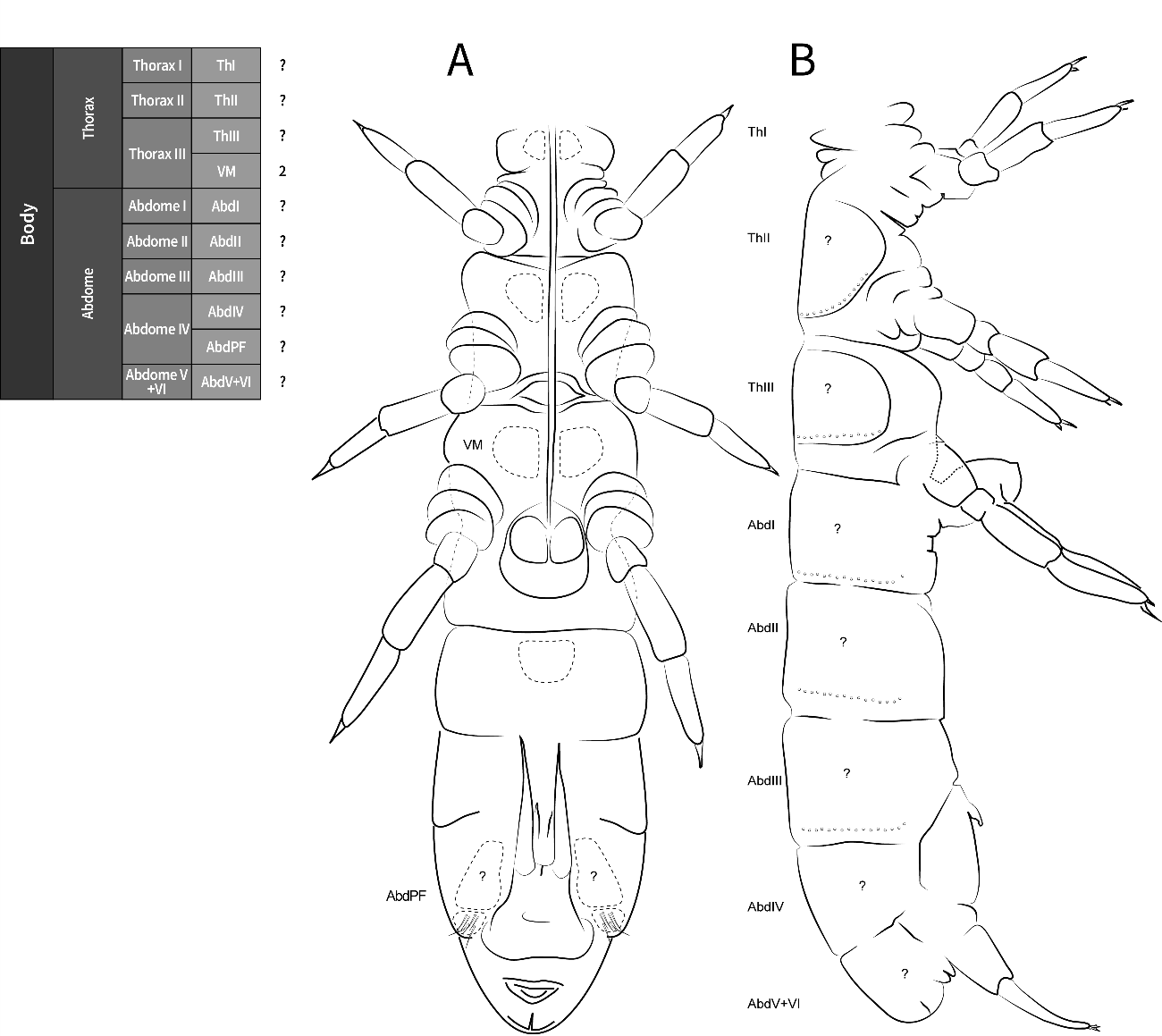


**Fig. S12** *Mucrosomia caeca.* Body chaetotaxy and descriptive table. (**A**) Body in ventral view; (**B**) Body in dorsal view. “?” indicates unavailable data


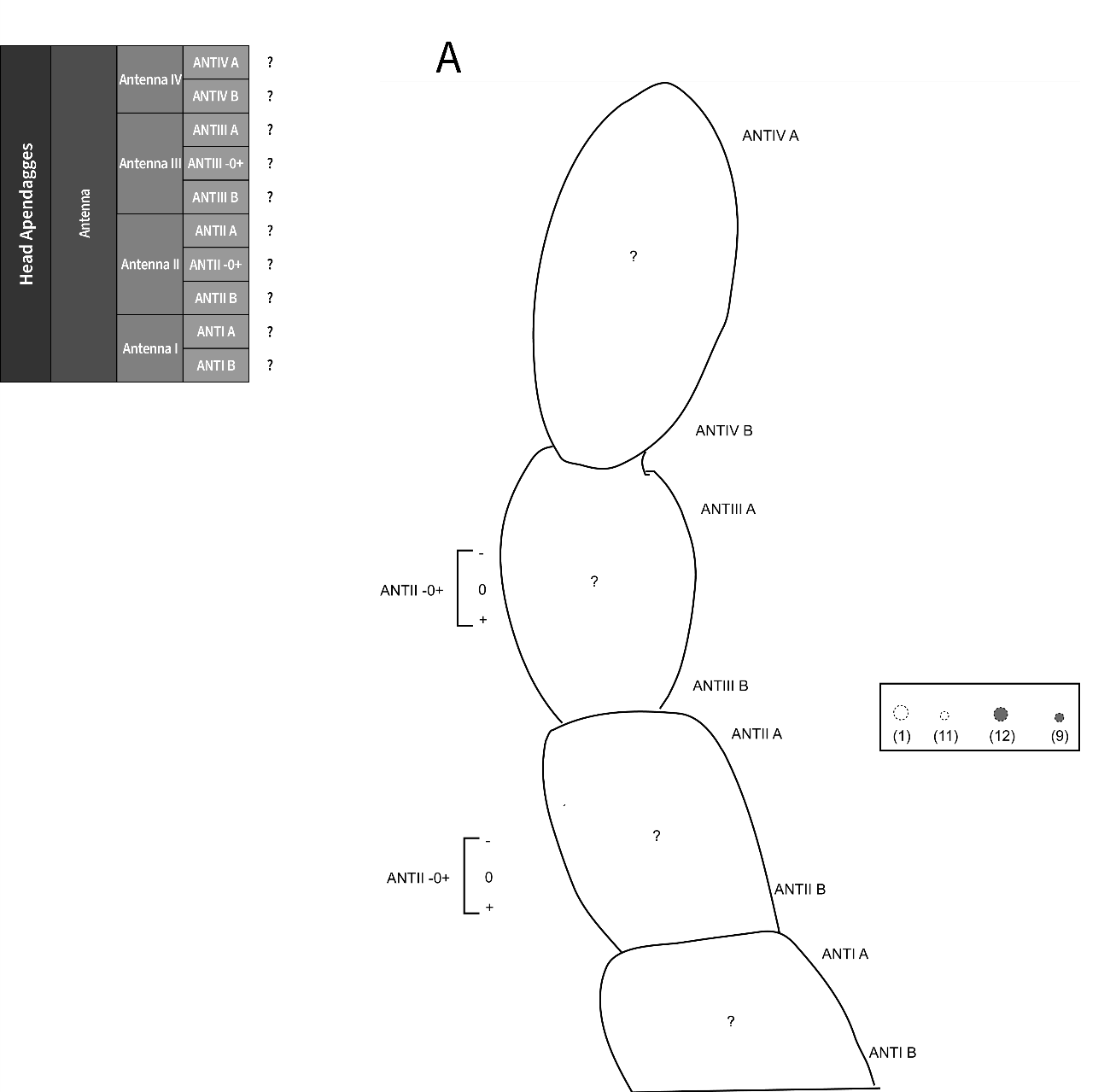


**Fig. S13** *Mucrosomia caeca.* Antennal chaetotaxy and descriptive table. (**A**) Entire antennal chaetotaxy. Chaetal types (1), (9), (11) and (12) follow those illustrated in the chaetal bank (see Fig.1)


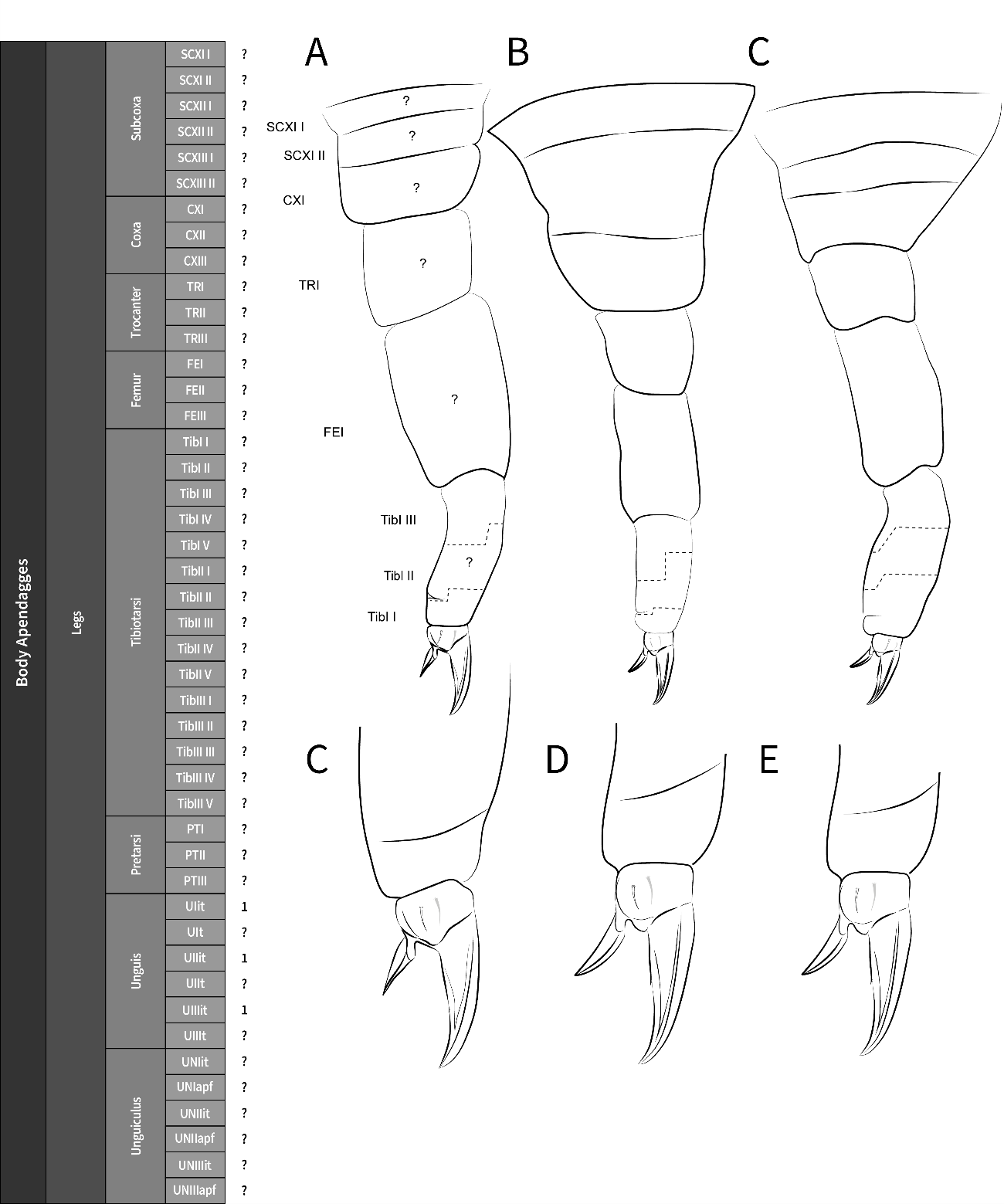


**Fig. S14** *Mucrosomia caeca.* Leg chaetotaxy and descriptive table. (**A**) Leg I; (**B**) Leg II; (**C**) Leg III; (**D**, **E**, **F**) Details of the apices of legs I–III. “?” indicates unavailable data


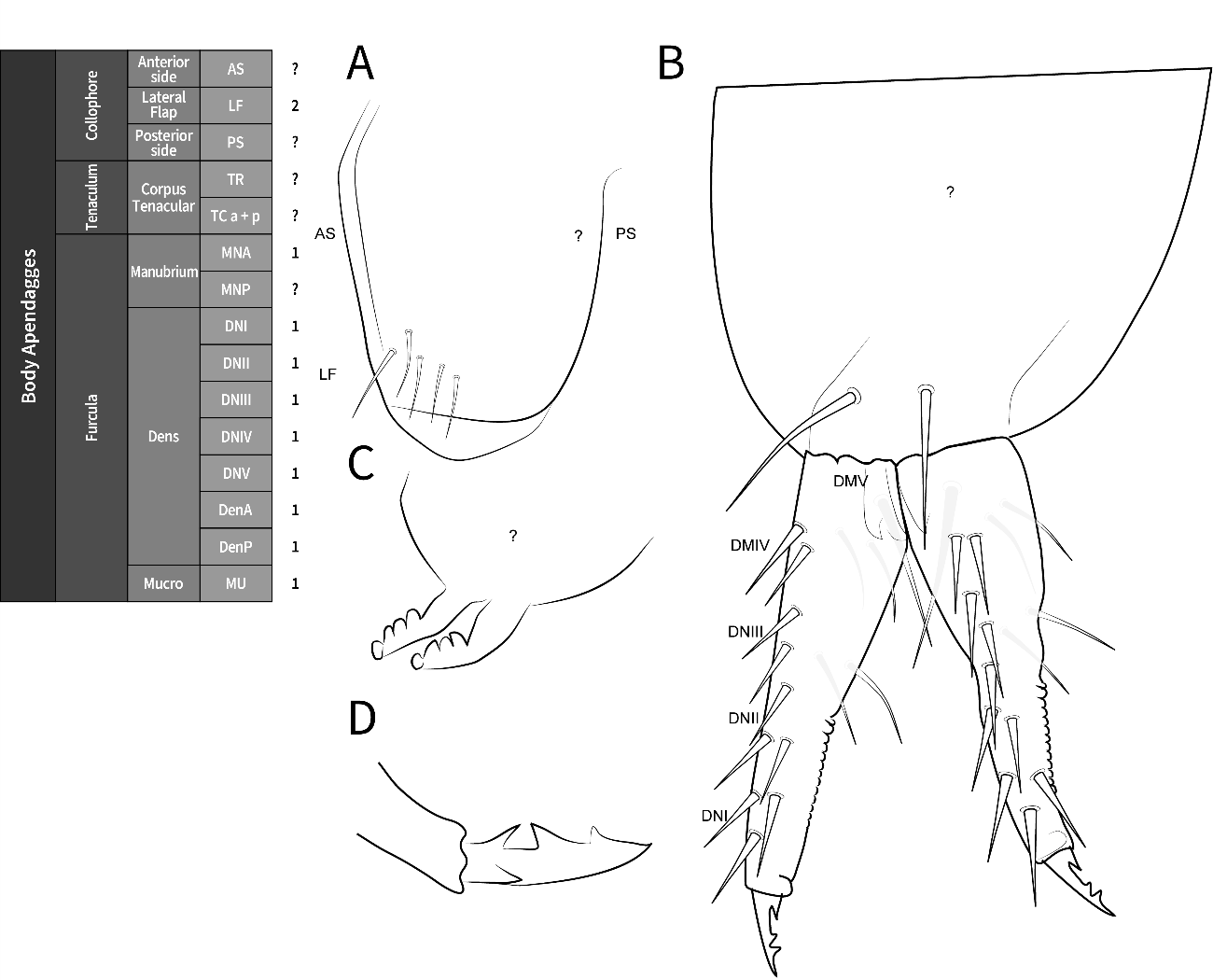


**Fig. S15** *Mucrosomia caeca.* Abdominal appendages chaetotaxy. (**A**) Collophore: anterior side (AS), lateral flap (LF), and posterior side (PS); (**B**) Furcula; (**C**) Tenaculum in anterior view; (**D**) Mucro. The lighter chaetae are located on the posterior side. “?” indicates unavailable data

***Mucrosomia garretti* (Bagnall, 1939)** (Figs. S16–S20)

**Complete Description:** Pigments (PI) absent. Body color (BC) White. Eye Number (EN) 0+0. Post-antennal organ form (PAO) present with constriction in the middle. Labral formula (a+m+p+pl) 4+5+5+3 8(1)9(2). Postlabial (PostL) 5+5 10(1). Labial triangle total chaetae (LBT) 10+10 20(1). Labial proximal chaetae and papillae chaetotaxy count (LBP) 26+26 8(1)8(3)2(4)2(5)32(6). Thorax I (ThI) 0+0. Thorax II (ThII) 6+6 2(8)8(9)2(10). Thorax III (ThIII) 4+4 2(8)6(9). Thorax III area ventralmedial (VM) 0+0. Abdomen I (AbdI) 6+6 6(8)4(9)2(10). Abdomen II (AbdII) 5+5 6(8)4(9). Abdomen III (AbdIII) 5+5 6(8)4(9). Abdomen IV (AbdIV) 7+7 8(8)6(9). Abdomen V and VI sensillar count (AbdV+VI) 5+5 10(9). Unguis I inner tooth (UIit) present. Unguis II inner tooth (UIIit) present. Unguis III inner tooth (UIIIit) present. Unguiculus I apical filament (UNIapf) absent. Unguiculus II apical filament (UNIIapf) absent. Unguiculus III apical filament (UNIIIapf) absent. Anterior side total chaetae (AS) 0. Lateral flap total chaetae (LF) 5-6+5-6 10-12(1). Posterior side total chaetae (PS) 3+3 6(1). Tenaculum rami (TR) 4+4 teeth. Tenaculum Corpus (a+p) (TC a+p) 2 2(1). Anterior manubrium total chaetae (MNA) 1+1 2(16). Dental whorl I total chaetae (DNI) 3 3(17). Dental whorl II total chaetae (DNII) 3 3(17). Dental whorl III total chaetae (DNIII) 4 2(1)2(17). Dental whorl IV total chaetae (DNIV) 4 2(1)2(17). Dental whorl V total chaetae (DNV) 1 1(17). Dental anterior total chaetae (DenA) 10+10 20(17). Dental posterior total chaetae (DenP) 5+5 8(1) 2(17). Mucro lamellae total teeth (MU) 5.

**Coded description:**


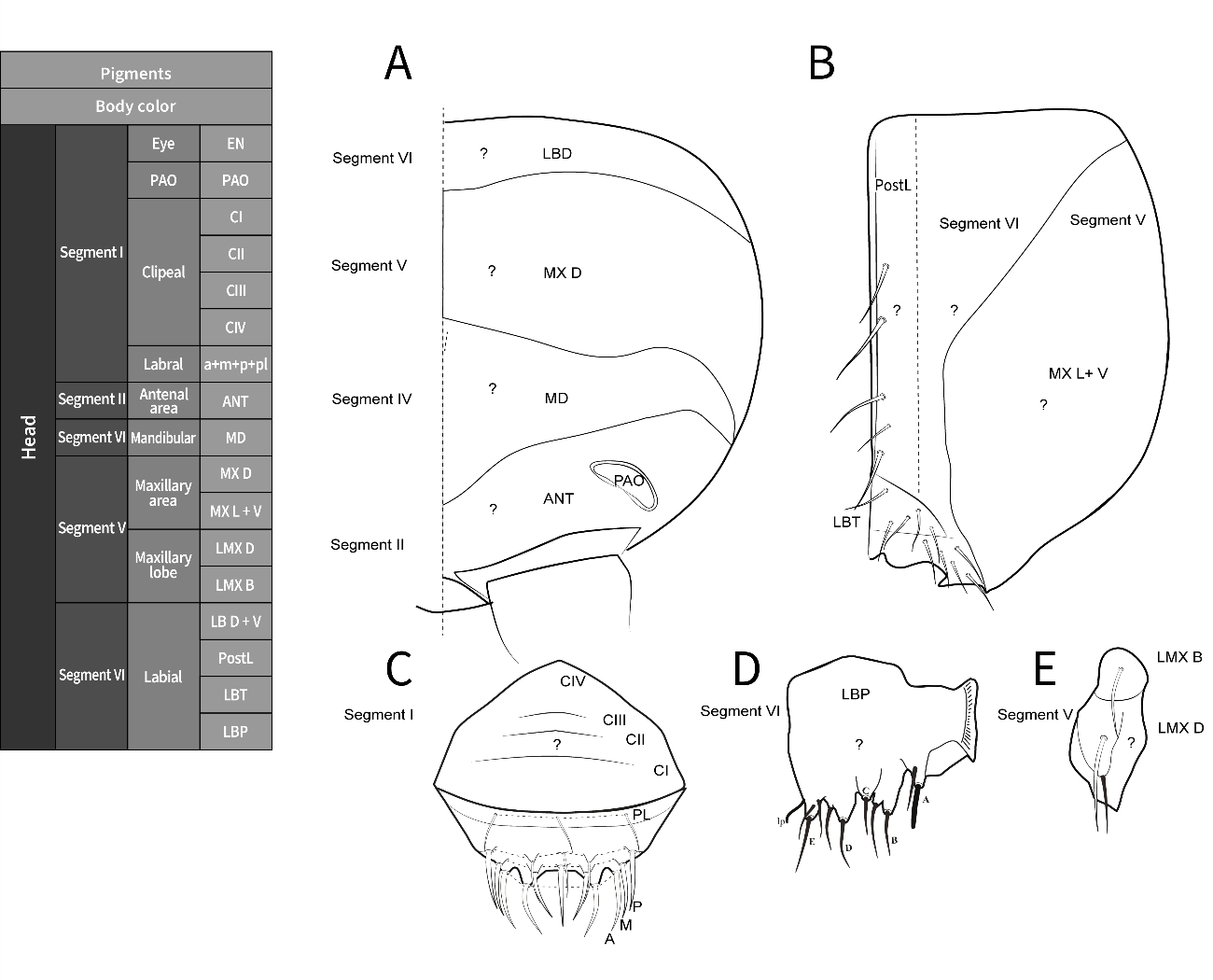


**Fig. S16** *Mucrosomia garretti.* Cephalic chaetotaxy and descriptive table. (**A**) Dorsal cephalic schematic chaetotaxy; (**B**) Labial triangle, medial and distal chaetotaxy; (**C**) Clypeal and labral chaetotaxy; (**D**) Labial proximal chaetotaxy; (**E**) Maxillary lobe. “?” indicates unavailable data


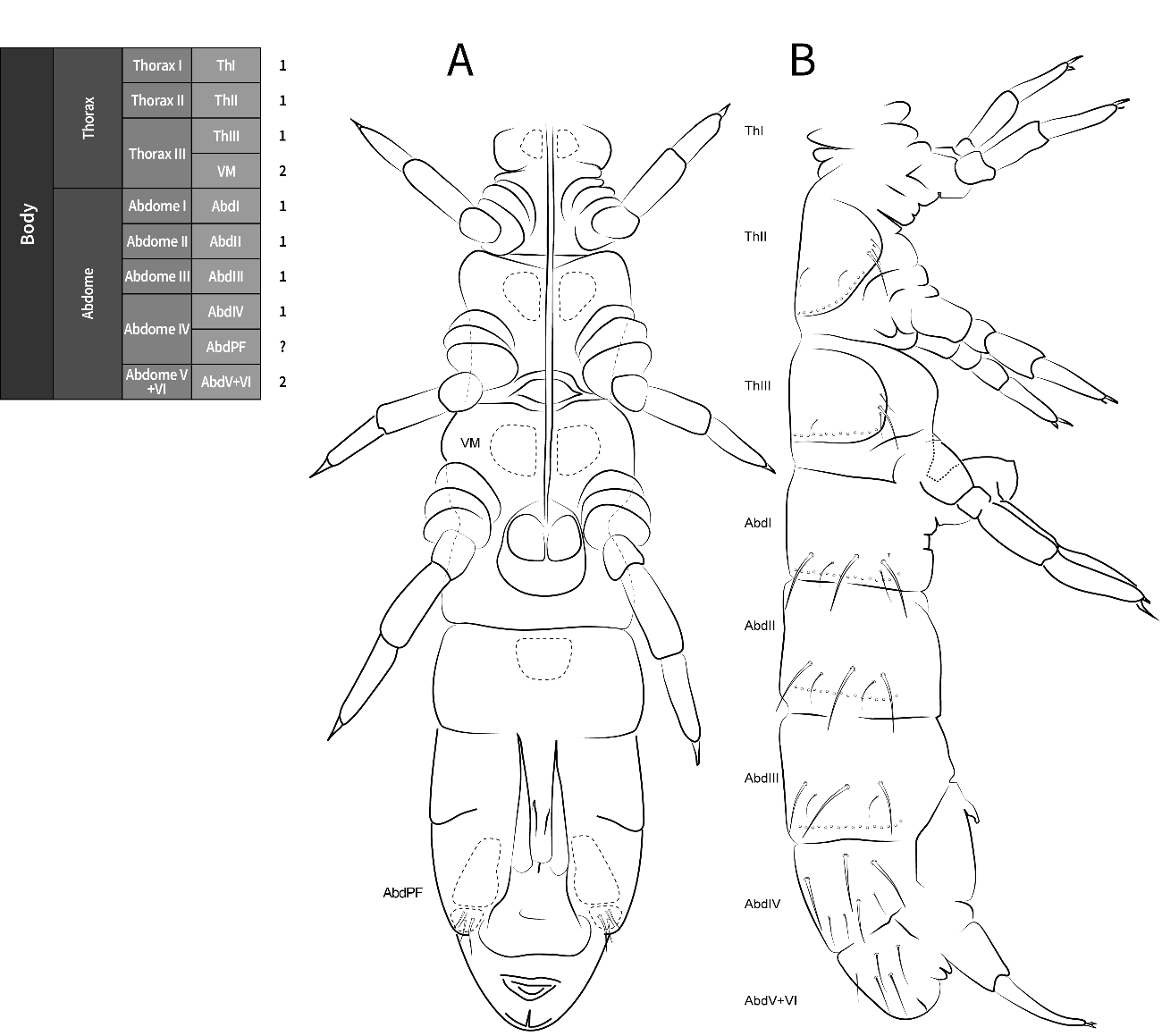


**Fig. S17** *Mucrosomia garretti.* Body chaetotaxy and descriptive table. (**A**) Body in ventral view; (**B**) Body in dorsal view. “?” indicates unavailable data


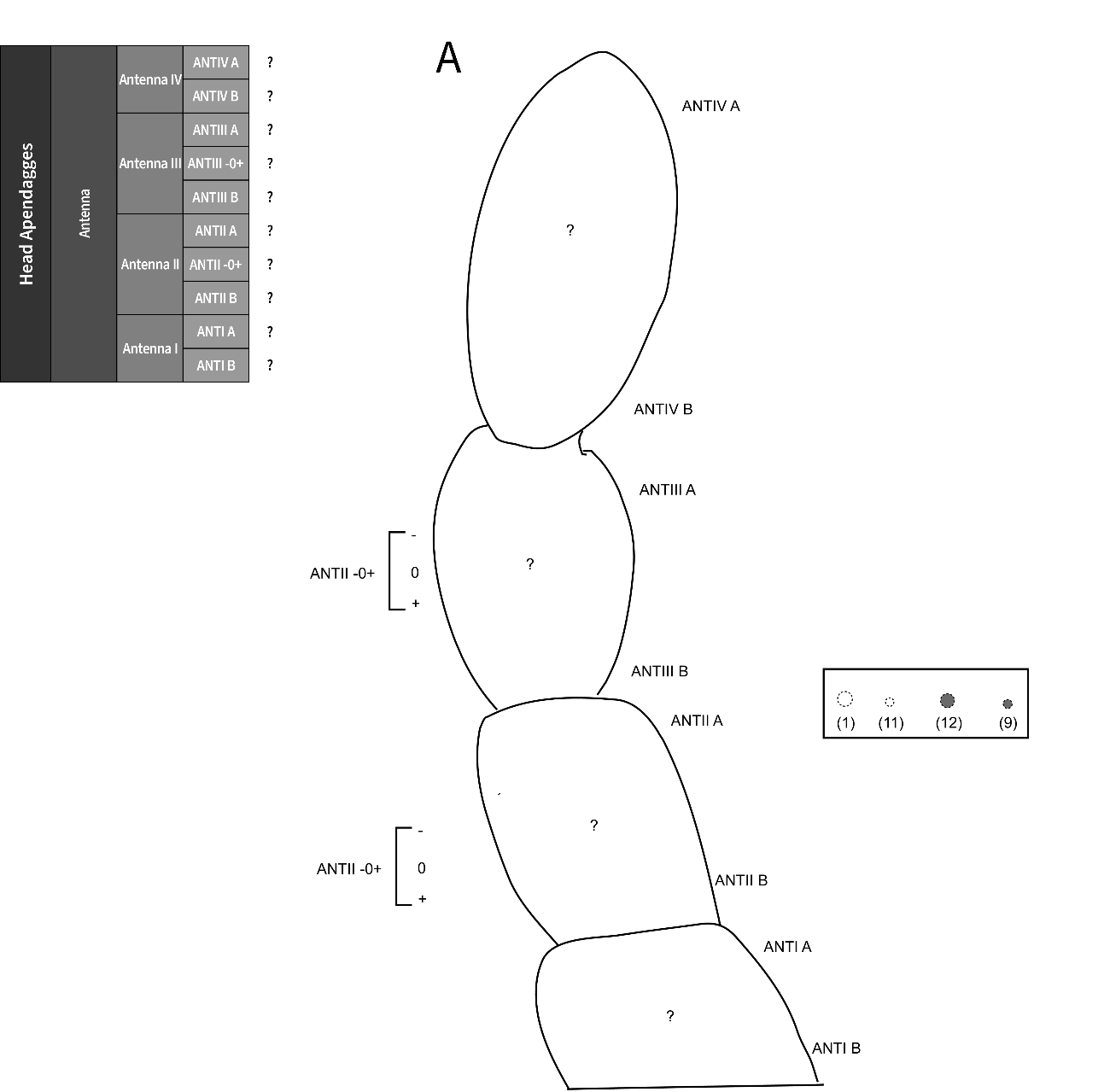


**Fig. S18** *Mucrosomia garretti.*Antennal chaetotaxy and descriptive table. (**A**) Entire antennal chaetotaxy. Chaetal types (1), (9), (11) and (12) follow those illustrated in the chaetal bank (see Fig. 1)


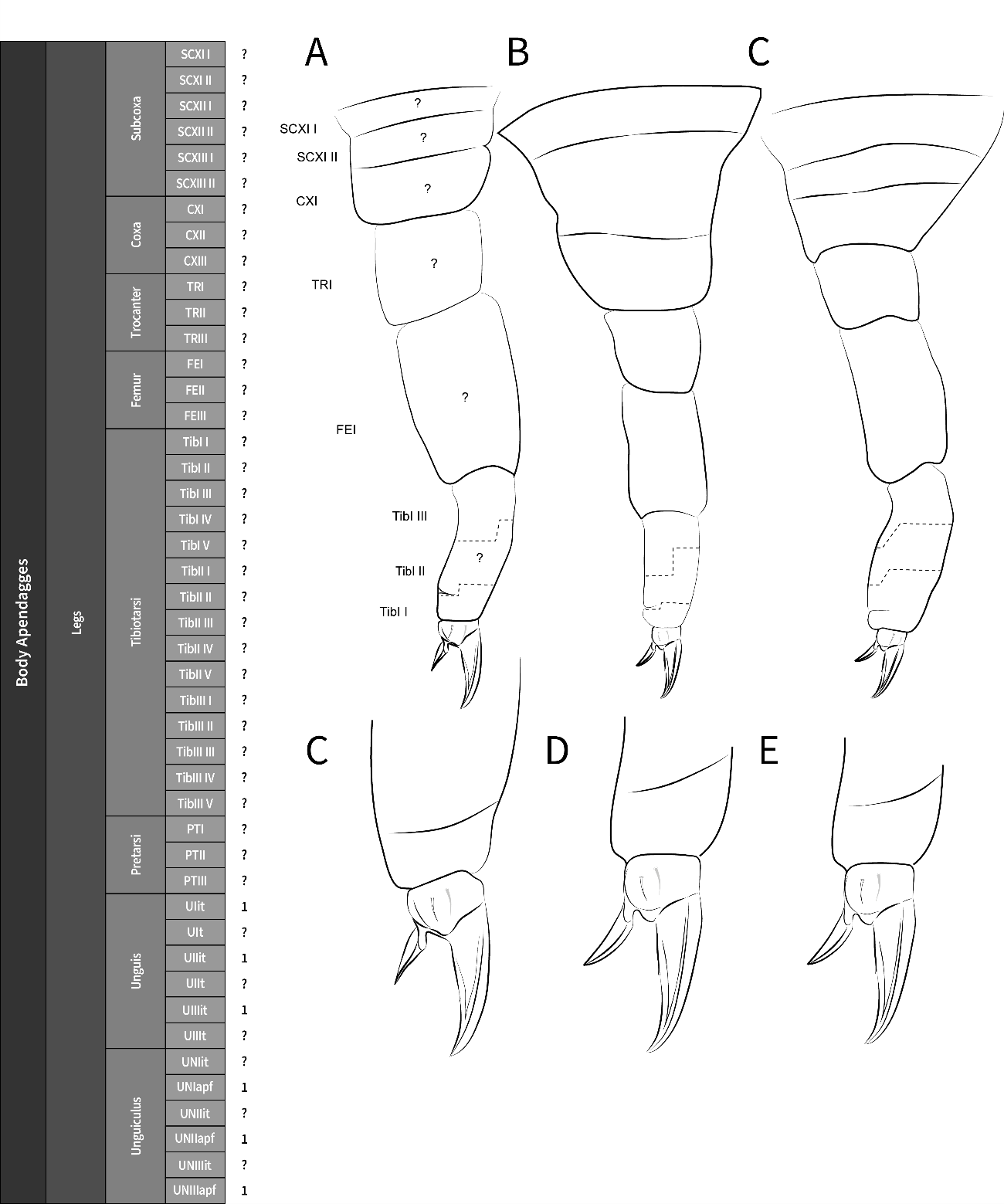


**Fig. S19** *Mucrosomia garretti.* Leg chaetotaxy and descriptive table. (**A**) Leg I; (**B**) Leg II; (**C**) Leg III; (**D**, **E**, **F**) Details of the apices of legs I–III. “?” indicates unavailable data


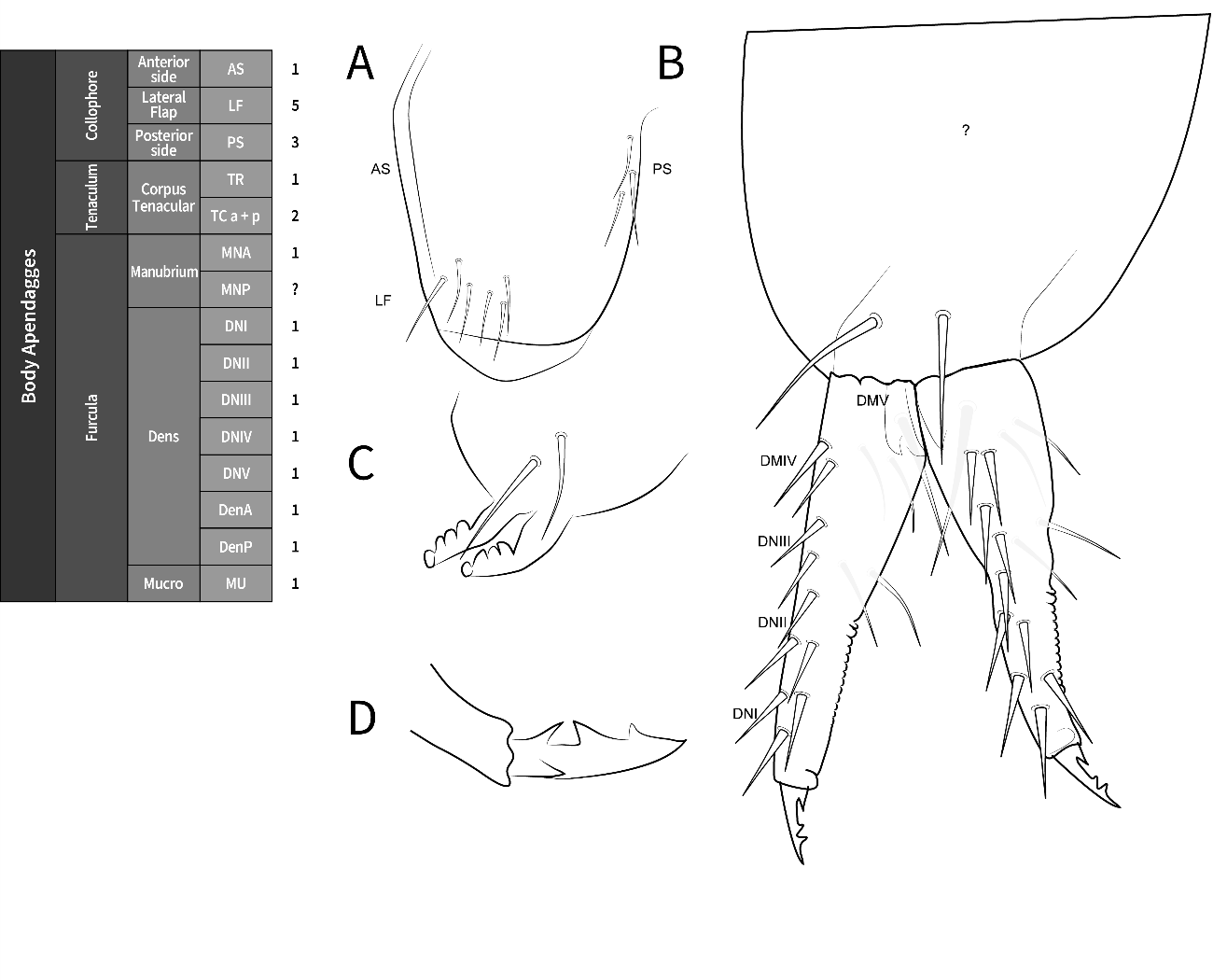


**Fig. S20** *Mucrosomia garretti.* Abdominal appendages chaetotaxy. (**A**) Collophore: anterior side (AS), lateral flap (LF), and posterior side (PS); (**B**) Furcula; (**C**) Tenaculum in anterior view; (**D**) Mucro. The lighter chaetae are located on the posterior side. “?” indicates unavailable data

***Mucrosomia novaezealandiae* (Salmon, 1943)** (Figs. S21–S25)

Pigments (PI) absent. Body color (BC) White. Eye Number (EN) 0+0. Post-antennal organ form (PAO) present with constriction in the middle. Thorax III area ventralmedial (VM) 0+0. Pretarsus I pretarsal chaetae (PTI) 2 2(11). Pretarsus II pretarsal chaetae (PTII) 2 2(11). Pretarsus III pretarsal chaetae (PTIII) 2 2(11). Unguis I inner tooth (UIit) absent. Unguis II inner tooth (UIIit) absent. Unguis III inner tooth (UIIIit) absent.

**Coded description:**


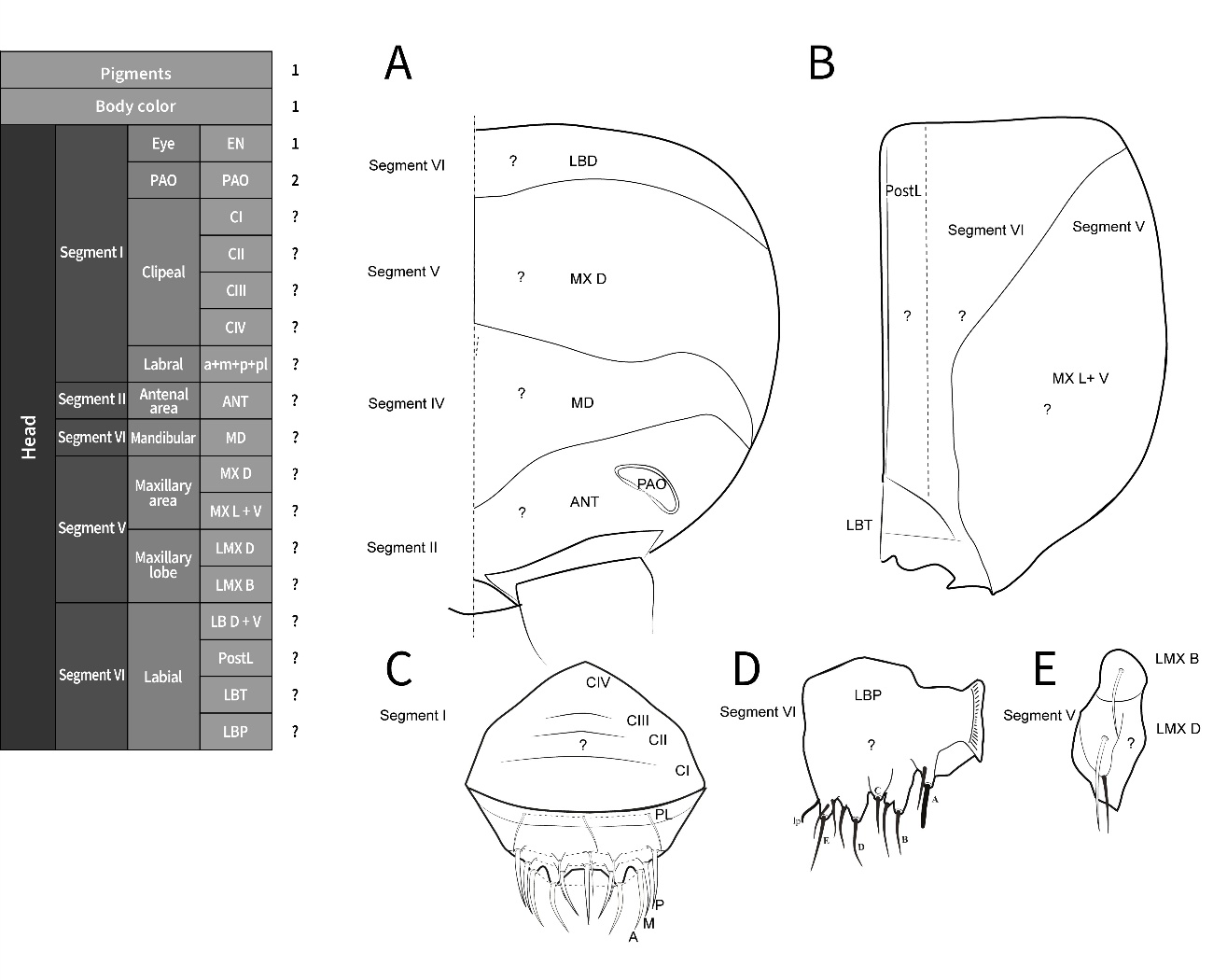


**Fig. S21** *Mucrosomia novaezealandiae.* Cephalic chaetotaxy and descriptive table. (**A**) Dorsal cephalic schematic chaetotaxy; (**B**) Labial triangle, medial and distal chaetotaxy; (**C**) Clypeal and labral chaetotaxy; (**D**) Labial proximal chaetotaxy; (**E**) Maxillary lobe. “?” indicates unavailable data


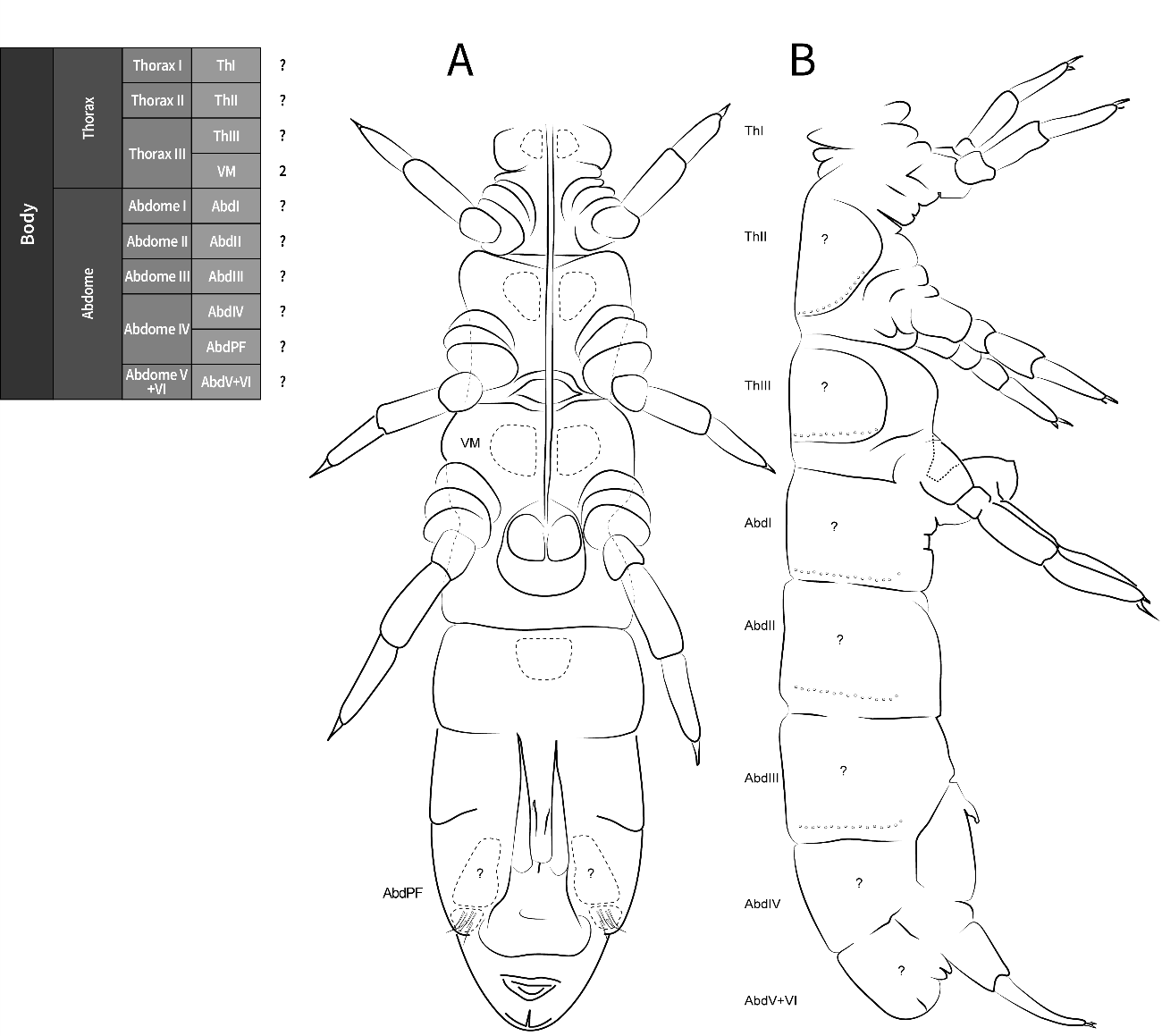


**Fig. S22** *Mucrosomia novaezealandiae.* Body chaetotaxy and descriptive table. (**A**) Body in ventral view; (**B**) Body in dorsal view. “?” indicates unavailable data


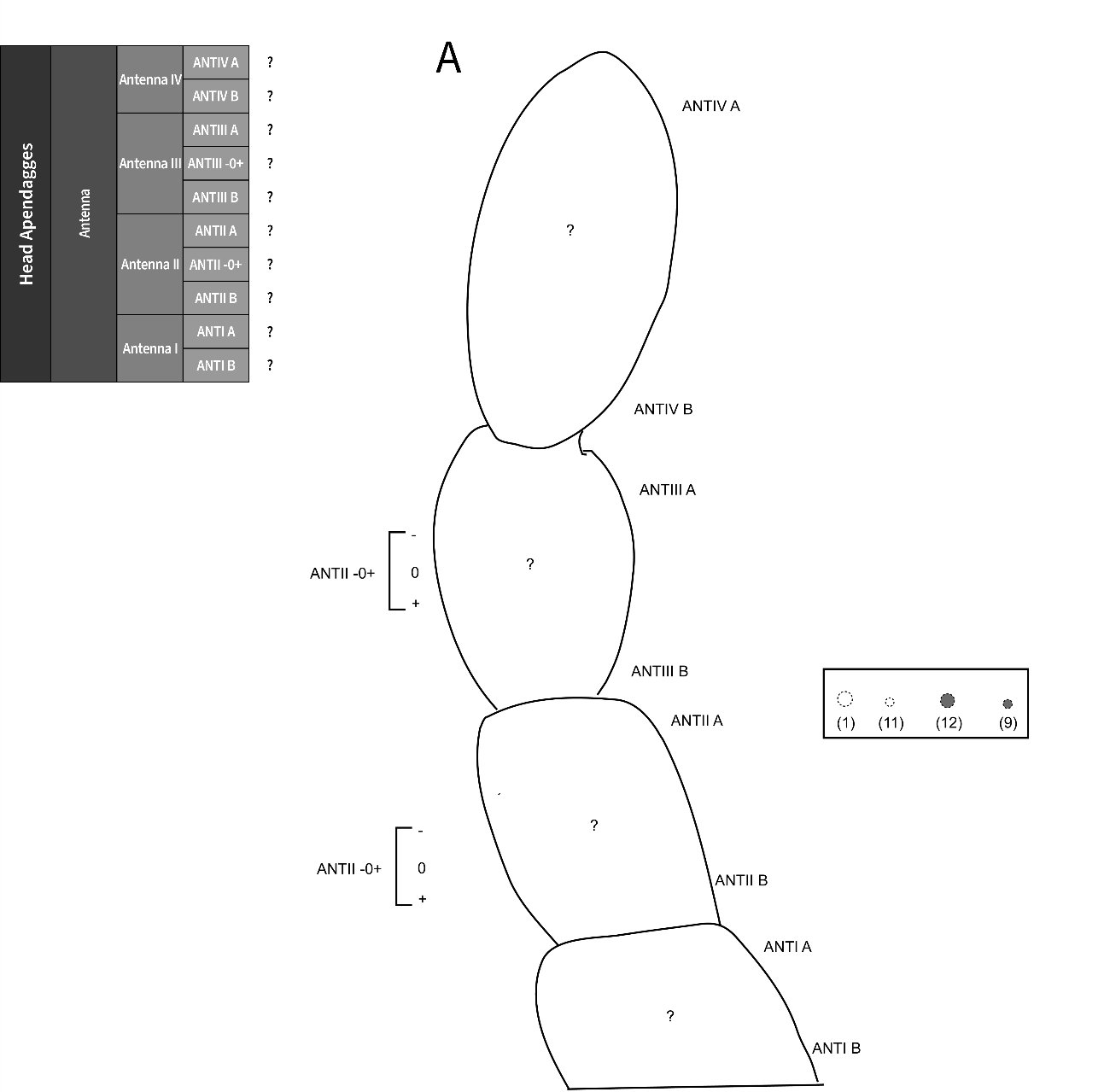


**Fig. S23** *Mucrosomia novaezealandiae.* Antennal chaetotaxy and descriptive table. (**A**) Entire antennal chaetotaxy. Chaetal types (1), (9), (11) and (12) follow those illustrated in the chaetal bank (see Fig. 1)


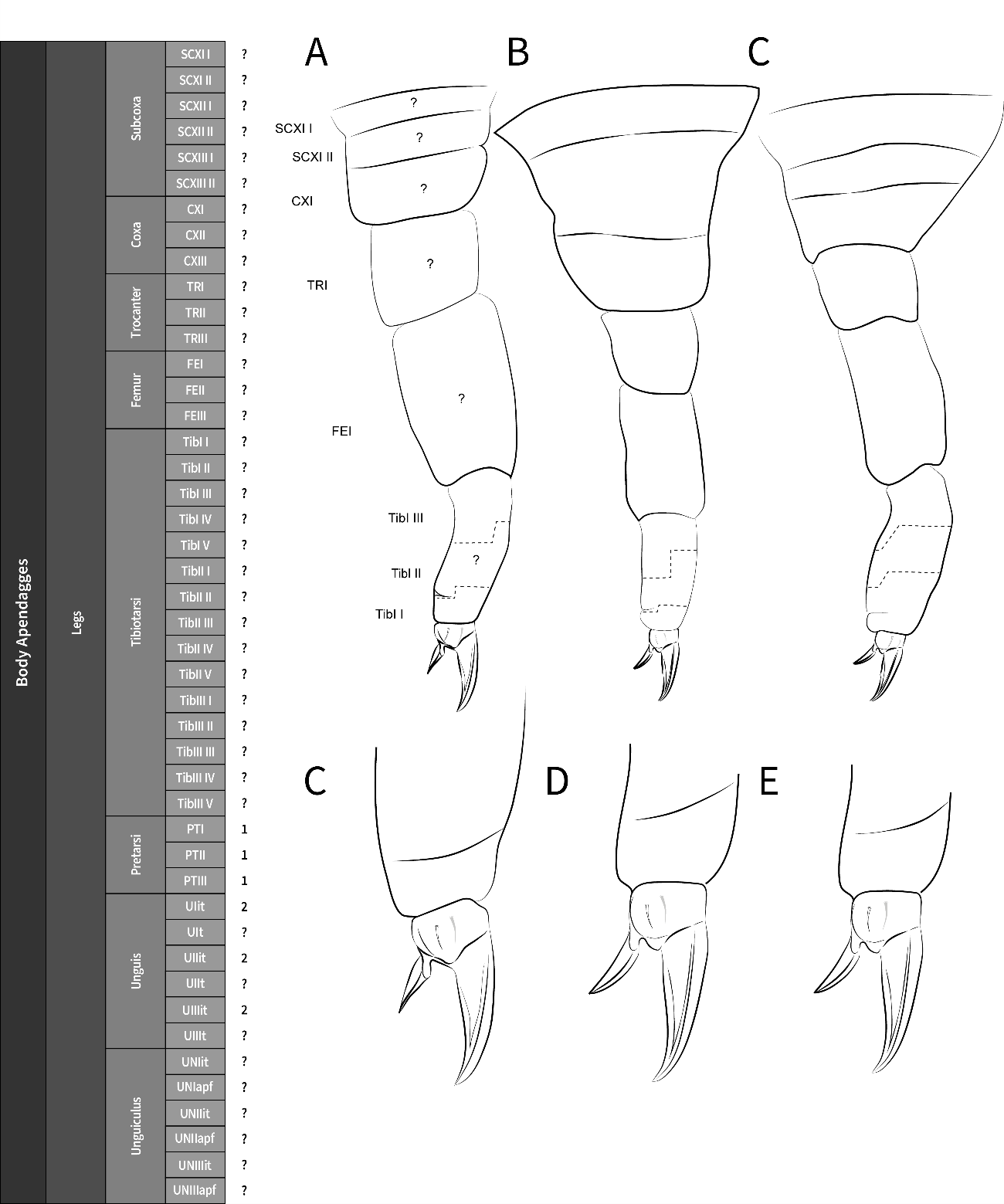


**Fig. S24** *Mucrosomia novaezealandiae.* Leg chaetotaxy and descriptive table. (**A**) Leg I; (**B**) Leg II; (**C**) Leg III; (**D**, **E**, **F**) Details of the apices of legs I–III. “?” indicates unavailable data


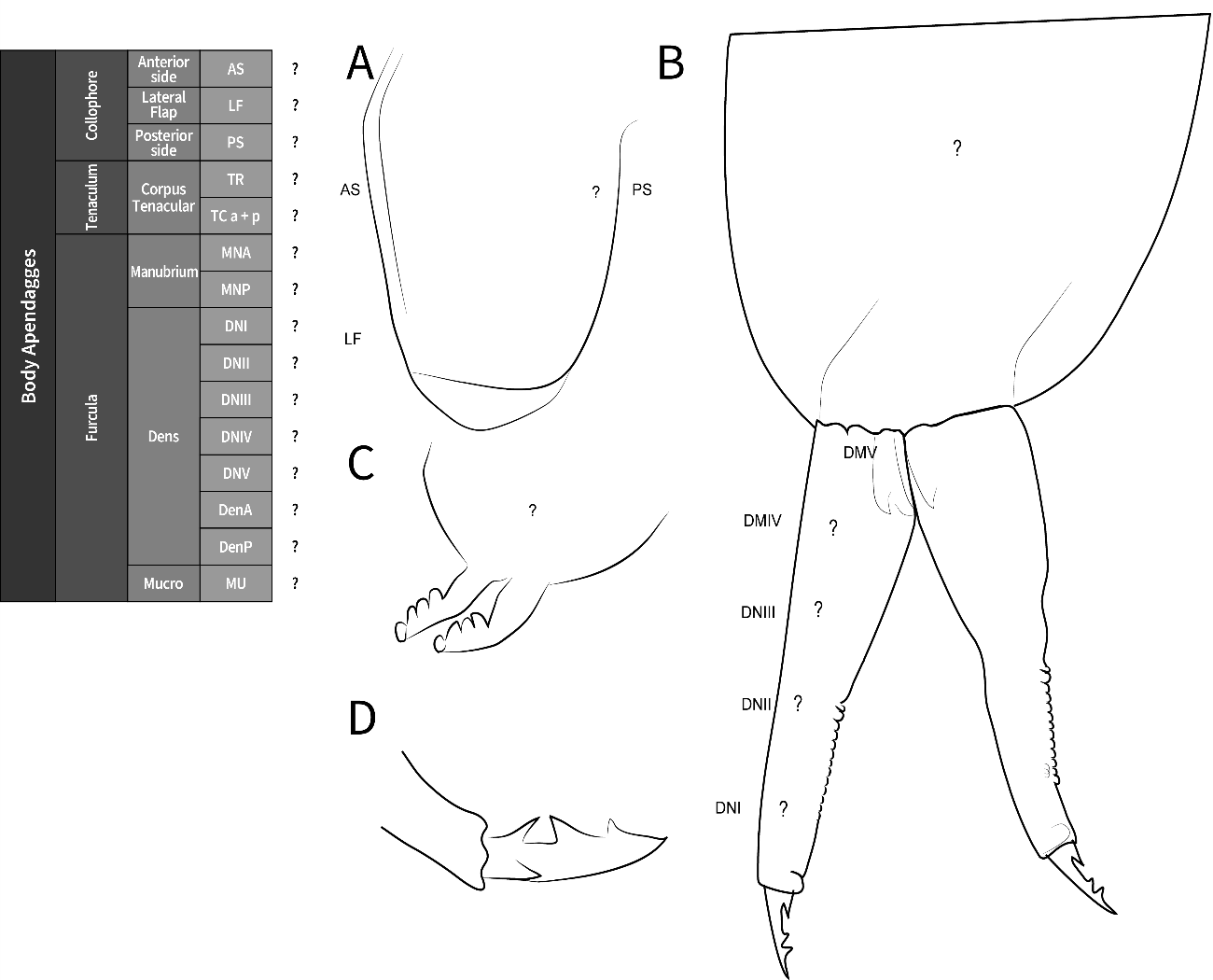


**Fig. S25** *Mucrosomia novaezealandiae.* Abdominal appendages chaetotaxy. (**A**) Collophore: anterior side (AS), lateral flap (LF), and posterior side (PS); (**B**) Furcula; (**C**) Tenaculum in anterior view; (**D**) Mucro. The lighter chaetae are located on the posterior side. “?” indicates unavailable data

**Interactive Map:** Map showing the global distribution of the genus *Mucrosomia*, with exact coordinates for the newly described species and approximate localities for previously described species based on the available literature. Interactive map can be accessed by double-clicking the button below

Click here
